# Photoinduced Reductive C–O Couplings from Unsymmetrical Bis-Cyclometalated Pt(IV) Dicarboxylato Complexes

**DOI:** 10.1021/acs.inorgchem.4c03667

**Published:** 2024-12-27

**Authors:** Juan Carlos López-López, Delia Bautista, Pablo González-Herrero

**Affiliations:** † Departamento de Química Inorgánica, Facultad de Química, Universidad de Murcia, Campus de Espinardo 19, Murcia 30100, Spain; ‡ Área Científica y Técnica de Investigación, Universidad de Murcia, Campus de Espinardo 21, Murcia 30100, Spain

## Abstract

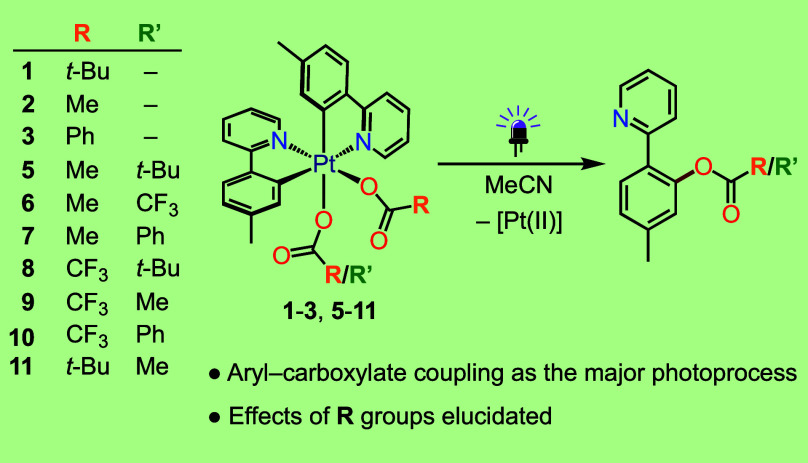

Unsymmetrical bis-cyclometalated dicarboxylato complexes (*OC*-6-32)-[Pt(tpy)_2_(O_2_CR)_2_] [tpy = cyclometalated 2-(*p*-tolyl)pyridine, R = *t*-Bu (**1**), Me (**2**), Ph (**3**), CF_3_ (**4**)], are obtained from the reaction of *cis*-[Pt(tpy)_2_] with the appropriate PhI(O_2_CR)_2_ reagent. Treatment of complexes of this type with different carboxylates (R’CO_2_^–^) results in the formation of mixed-carboxylato derivatives, namely (*OC*-6-43)-[Pt(tpy)_2_(O_2_CMe)(O_2_CR′)] [R′ = *t*-Bu (**5**), CF_3_ (**6**), Ph (**7**)], (*OC*-6-34)-[Pt(tpy)_2_(O_2_CCF_3_)(O_2_CR′)] [R′ = *t*-Bu (**8**), Me (**9**), Ph (**10**)], and (*OC*-6-34)-[Pt(tpy)_2_(O_2_C-*t*-Bu)(O_2_CMe)] (**11**). Irradiation of **1**–**3** and **5**–**11** with UV light (365 nm) in MeCN gives 5-methyl-2-(2-pyridyl)phenyl pivalate (**12**), 5-methyl-2-(2-pyridyl)phenyl acetate (**13**) or 5-methyl-2-(2-pyridyl)phenyl benzoate (**14**) as the major photoproduct from most complexes, resulting from a reductive C–O coupling between a tpy ligand and a carboxylato ligand. Cyclometalation of **12**–**14** at the ensuing Pt(II) species to produce *cis*-[Pt(tpy)(tpyO_2_CR/R′)], reduction to *cis*-[Pt(tpy)_2_] and isomerization to (*OC*-6-33)-[Pt(tpy)_2_(O_2_CR/R′)_2_] are identified as secondary processes in most cases. In contrast, complex **4** exclusively photoisomerizes to (*OC*-6-33)-[Pt(tpy)_2_(O_2_CCF_3_)_2_] (**4′**). The C–O couplings are favored for the most electron-rich carboxylato ligands and occur predominantly from the carboxylato trans to N. Consistent with this, a computational study reveals that the lowest singlet and triplet LMCT excited states result from electronic transitions to a dσ* orbital distributed along the N–Pt–O axis, which would trigger the observed processes.

## Abstract 2

Reductive aryl−carboxylate couplings are demonstrated as the major photoprocesses taking place from the irradiation of unsymmetrical bis-cyclometalated Pt(IV) dicarboxylato complexes with UV light. The factors that influence these reactions are elucidated.

## Introduction

The reactivity of electronically excited transition-metal organometallic complexes is currently gaining relevance in the context of the development of photochemical catalyzed processes. In particular, recent accounts have highlighted the potential of new methodologies within the framework of light-induced transition-metal catalysis, where the metal complex not only performs the function of light absorption, but also executes bond-breaking and bond-forming steps.^1−3^ The formation of C–heteroatom bonds is a key focus in the advancement of synthetic tools because of the widespread presence of such bonds in natural products and pharmaceuticals.^4^ The utilization of light irradiation to promote these processes under mild conditions has gained significant traction, aligning with the increasing demand for energy-efficient strategies, and recent studies have revealed a variety of reductive couplings that can take place from transition-metal complexes in their excited state, which have been integrated into catalytic processes.^5−14^ Among them, aryl-carboxylate couplings are worthy of special note,^13,14^ since these reactions are considered to be particularly difficult under thermal conditions due to the low nucleophilicity of carboxylates.^15^

The photochemistry of Pt(IV) complexes is dominated by metal–ligand bond cleavage and reductive processes, which are triggered by ligand-to-metal charge-transfer (LMCT) excited states involving electronic transitions to the strongly antibonding dσ* orbitals.^16^ This reactivity has been exploited as the basis for the development of photoactivatable prodrugs intended for the localized treatment of cancer,^17−21^ many of which are Pt(IV) dicarboxylato complexes that, upon photoexcitation, are reduced to the pharmacologically active Pt(II) species, with the release of carboxyl radicals.^22^ In addition, photochemical studies on Pt(IV) complexes have demonstrated the release of hydroxyl radicals,^23,24^ or C–halogen reductive couplings resulting in the halogenation of aromatic compounds.^25,26^

**Scheme 1:**
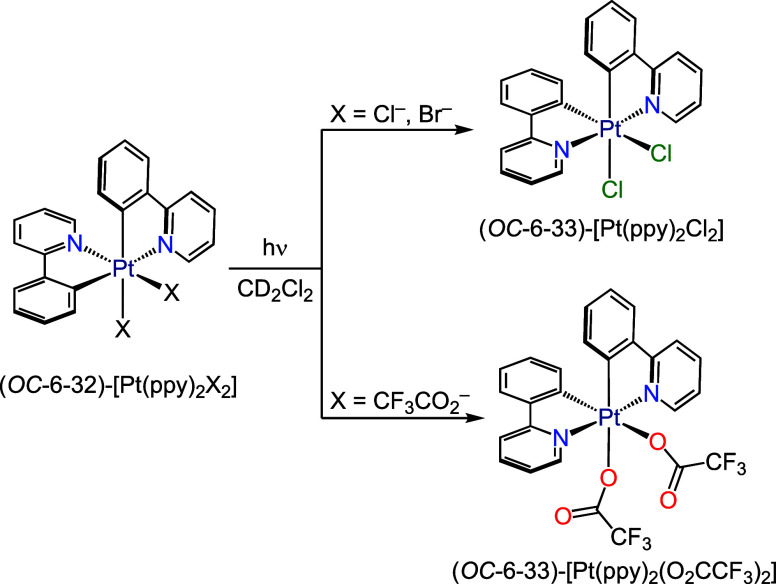
Previously Reported Photoisomerizations of Bis-Cyclometalated Pt(IV) Complexes ppy = Cyclometalated 2-phenylpyridine.

Several classes of Pt(IV) complexes with cyclometalated 2-arylpyridines (C∧N) and related heteroaromatic ligands have been developed in recent years, some of which are capable of producing efficient phosphorescent emissions from triplet ligand-centered excited states (^3^LC or ^3^π–π*) with very little metal-to-ligand charge-transfer (MLCT) character. The highest emission efficiencies are attained for tris-cyclometalated complexes with a facial geometry, *fac*-[Pt(C∧N)_3_]^+^,^27−29^ bis-cyclometalated complexes of the type [Pt(C∧N)_2_(R/Ar)(X)],^30,31^ which also feature a facial disposition of metalated *C*-donor moieties, and derivatives with cyclometalated aryl-*N*-heterocyclic carbene ligands.^32−34^ In all these classes, the mutual arrangement and strong σ donating ability of the ligands induce a strong ligand-field splitting that raises dσ* orbitals, making ^3^LMCT states thermally inaccessible from the emissive ^3^LC states. In contrast, bis-cyclometalated Pt(IV) complexes lacking strong-field ligands tend to show weaker emissions or, in some cases, photochemical reactivity, due to the presence of accessible ^3^LMCT excited states.^35,36^ In particular, the unsymmetrical complexes (*OC*-6-32)-[Pt(ppy)_2_X_2_], where ppy is cyclometalated 2-phenylpyridine and X = Cl^–^, Br^–^ or CF_3_CO_2_^–^, undergo photoisomerization to the *C*_2_-symmetrical complexes (*OC*-6-33)-[Pt(ppy)_2_X_2_] upon exposure to UV light in CD_2_Cl_2_ (Scheme 1).^35^ In the case of the bromo derivative, an halogen-exchange reaction with the solvent was demonstrated, which supported a radical mechanism initiated by the homolytic cleavage of Pt–Br bonds. The same mechanism was inferred for the chloro derivative, whereas an heterolytic cleavage of Pt–O bonds was proposed to explain the isomerization of the trifluoroacetato derivative. Recently, we reported a study on the photoreactivity of unsymmetrical bis-cyclometalated alkynyl(carboxylato) Pt(IV) complexes and showed that, depending on the electronic properties of the alkynyl and carboxylato ligands, C–C and/or C–O reductive couplings involving one of the cyclometalated ligands were induced by irradiation with UV light.^37^

Various C(sp^3^)–O reductive couplings have been reported from Pt(IV) methyl complexes in recent decades, involving carboxylato, hydroxide, or aryloxido ligands, which usually require heating.^38−44^ However, achieving C(sp^2^)–O reductive couplings from Pt(IV) complexes is challenging and the first examples of Aryl–O couplings from well-defined and isolable Pt(IV) complexes were reported in 2020,^45^ which occur thermally and require fluorido ligands in the coordination sphere. Following our previous experience, we decided to further explore photochemical pathways to C–heteroatom couplings from Pt(IV) complexes. In the present work, we investigate the photoreactivity of a series of unsymmetrical bis-cyclometalated Pt(IV) complexes bearing either two identical or two different carboxylato ligands, with the aim to exclusively promote C(sp^2^)–O couplings between 2-arylpyridine and carboxylato ligands, and to understand the electronic properties and conditions that favor these couplings.

## Results and Discussion

### Synthesis and Characterization of Unsymmetrical Bis-cyclometalated Pt(IV) Dicarboxylato Complexes

**Scheme 2:**
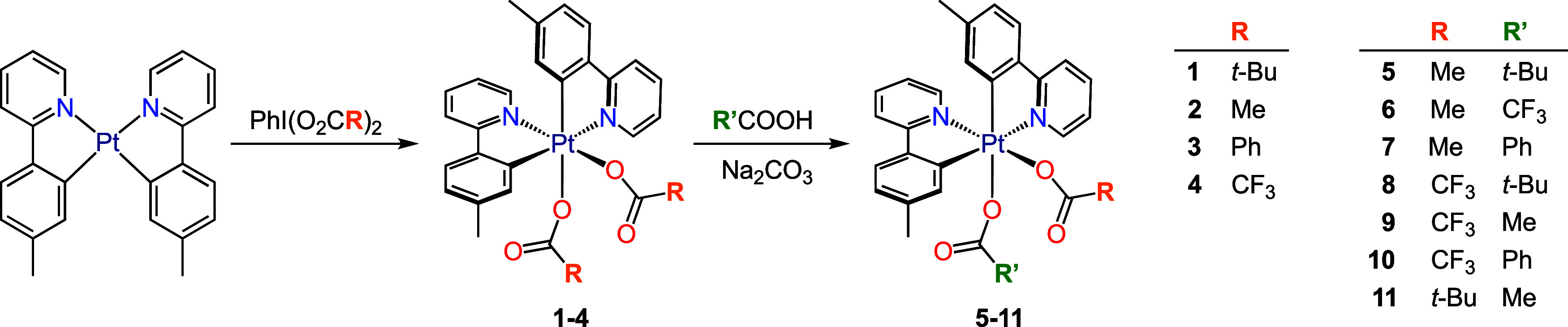
Synthesis of Unsymmetrical Bis-cyclometalated Pt(IV) Dicarboxylato Complexes

Previous studies by Whitfield and Sanford^46,47^ explored the oxidation of the Pt(II) complex *cis*-[Pt(ppy)_2_] with the hypervalent iodine reagents PhI(O_2_CR)_2_ (R = Me, CF_3_), which produces Pt(IV) complexes of the type (*OC*-6-32)-[Pt(ppy)_2_(O_2_CR)_2_], featuring an unsymmetrical arrangement of cyclometalated ligands and mutually cis, monodentate carboxylato ligands. The synthesis of the analogous complexes (*OC*-6-32)-[Pt(tpy)_2_(O_2_CR)_2_] [tpy = 2-(*p*-tolyl)pyridine; R = Me (**2**), CF_3_ (**4**)] (Scheme 2) has been recently reported by us using a similar methodology.^37^ For the present study, the derivatives with R = *t*-Bu (**1**) and Ph (**3**) were prepared in good yields (80 or 78%, respectively) from the reactions of *cis*-[Pt(tpy)_2_] with the appropriate PhI(O_2_CR)_2_ reagent. Solutions of complexes **1**, **2** and **4** in CH_2_Cl_2_ were treated with an excess of different carboxylates, which were generated in situ by adding the corresponding acids in the presence of Na_2_CO_3_. These reactions produced mixed-carboxylato derivatives resulting from the substitution of one of the carboxylato ligands by the carboxylato derived from the added acid. Thus, complexes (*OC*-6-43)-[Pt(tpy)_2_(O_2_CMe)(O_2_CR′)], with R′ = *t*-Bu (**5**), CF_3_ (**6**), Ph (**7**), were obtained from **2** using pivalic, trifluoroacetic, or benzoic acid, whereas (*OC*-6-34)-[Pt(tpy)_2_(O_2_CCF_3_)(O_2_CR′)], with R′ = *t*-Bu (**8**), Me (**9**), Ph (**10**), were obtained from **4** using pivalic, acetic, or benzoic acid (Scheme 2). To further test the influence of the coordination position of the carboxylato ligands on the observed photoreactivity, the derivative (*OC*-6-34)-[Pt(tpy)_2_(O_2_C-*t*-Bu)(O_2_CMe)] (**11**) was obtained from **1** using acetic acid. All the mixed-carboxylato complexes were isolated in good yields (70–91%).

These substitution reactions also proceeded in the absence of Na_2_CO_3_, but did not complete after 20 h, suggesting that an exchange equilibrium was reached. In the presence of Na_2_CO_3_, the reactions were complete within 20 h at room temperature, excepting those leading to complexes **6**, **7** or **10**, which required heating at 40 °C. In all cases, only one of the carboxylato ligands was substituted, even when a large excess of the added acid was employed. Also, a single isomer was obtained, resulting from the selective substitution of the carboxylato ligand trans to the metalated *p-*tolyl group of one of the tpy ligands, and not the one trans to a pyridyl ring, as inferred from the NMR data (see below). Given the stronger kinetic *trans* effect of the metalated *p-*tolyl as compared to the pyridyl, the observed selectivity suggests that the substitutions most likely proceed via a dissociative mechanism.

**Figure 1:**
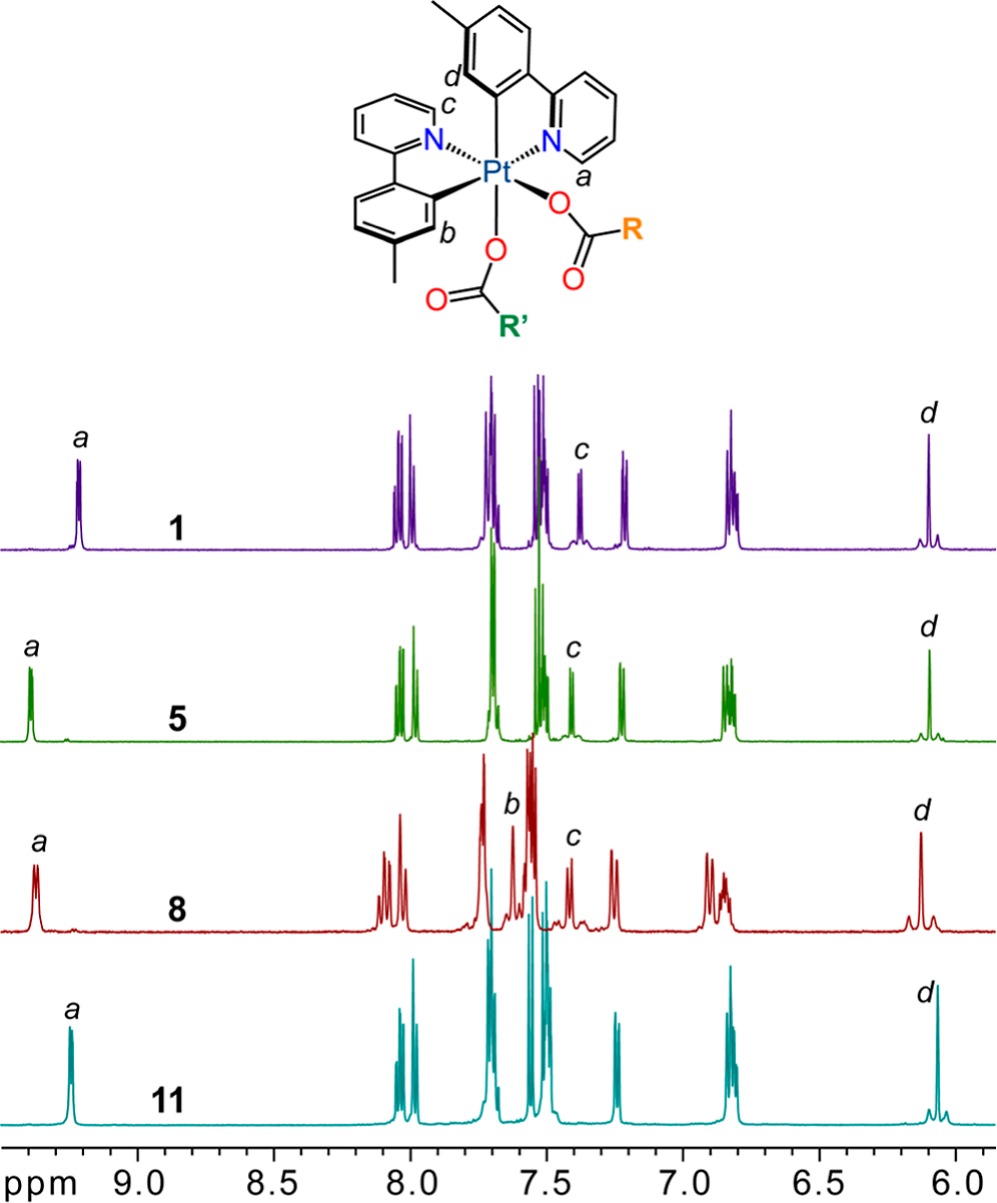
^1^H NMR spectra of complexes **1**, **5**, **8** and **11** (aromatic region). The signals corresponding to protons *b* and *c* cannot be distinguished in some cases.

As previously observed for **2** and **4**,^37^ the ^1^H NMR spectra of complexes **1**, **3**, and **5**–**11** show two sets of aromatic resonances, consistent with the unsymmetrical arrangement of the tpy ligands. As an illustration, the spectra of complexes **1**, **5**, **8** and **11** are shown in Figure 1. The resonances arising from the protons ortho to the metalated carbon atoms (*b*, *d*) or the coordinated nitrogen atoms (*a*, *c*) show satellites or a broadening at the base, respectively, due to their coupling with the ^195^Pt isotope. Proton *c* (range 7.36–7.48 ppm) is strongly shielded with respect to *a* (9.2–9.4 ppm) because it is directed toward the shielding region generated by the diamagnetic current of the orthogonal pyridine ring. A similar situation is found for protons *b* (7.66–7.90 ppm) and *d* (6.04–6.19 ppm). In the cases of the mixed-carboxylato derivatives, the presence of two different carboxylato ligands is demonstrated by the resonances of the R and R′ groups in the ^1^H and/or ^19^F NMR spectra (see the Supporting Information). The ^19^F NMR spectra of **6** and **8**–**10** demonstrate the coordination position of the trifluoroacetato ligand, because ^195^Pt satellites are observed when it is trans to a coordinated N atom, but not when it is trans to a metalated C atom. Thus, the CF_3_ group gives a singlet resonance without satellites for **6**, where the trifluoroacetato is trans to the metalated C atom, whereas a singlet with satellites is observed for derivatives **8**–**10**, where the ligand is trans to the coordinated N atom. We have observed this phenomenon for other Pt(IV) complexes bearing one or two trifluoroacetato ligands.^37^ It indicates that the transmission of spin–spin coupling between the Pt and F nuclei is less effective when the trifluoroacetato is coordinated trans to C, which can be explained by a weaker metal–ligand bond due to the stronger trans influence of the metalated *p*-tolyl, in agreement with the observed longer Pt–O bond distances with respect to those trans to N (see below).

**Figure 2:**
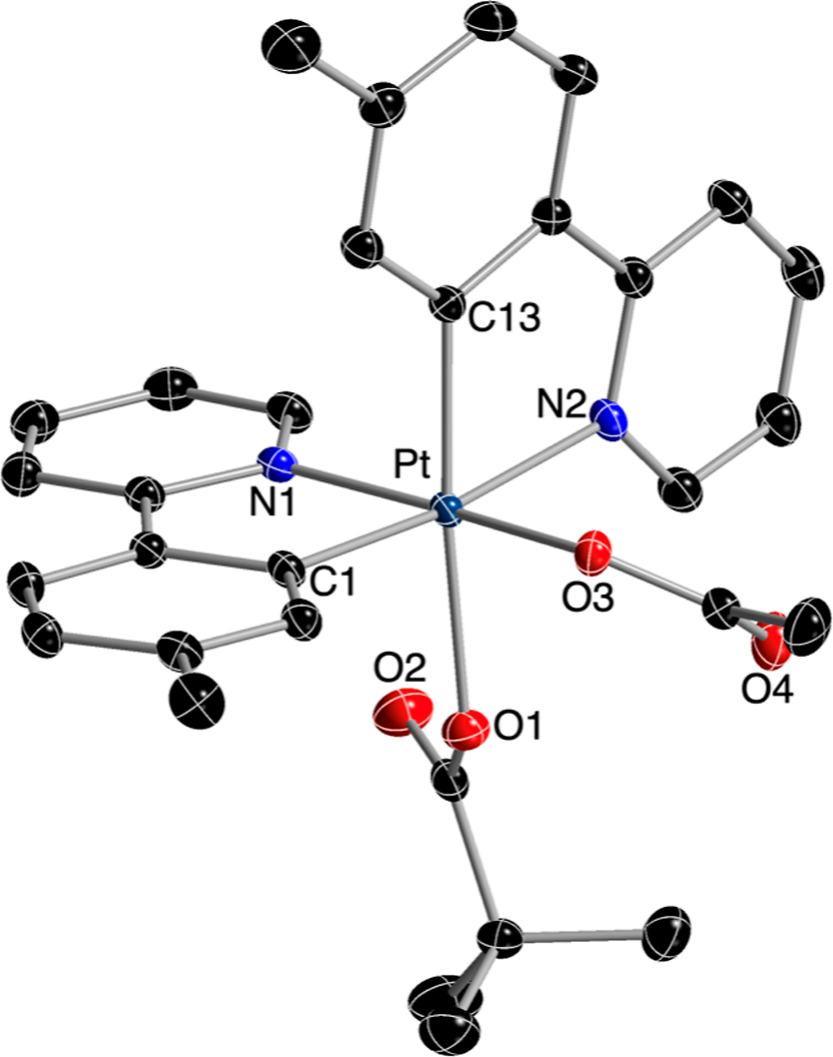
Structure of complex **5** in the crystal (thermal ellipsoids at 50% probability). Hydrogen atoms are omitted. Selected bond distances (Å) and angles (deg): Pt–C1, 1.999(2); Pt–C13, 2.012(2); Pt–O3, 2.0215(16); Pt–N1, 2.0222(19); Pt–O1, 2.1243(16); Pt–N2, 2.1471(19); C13–Pt–N1, 90.14(8); C1–Pt–N1, 81.41(8); C1–Pt–O1, 87.58(8); O3–Pt–O1, 86.80(7); O3–Pt–N2, 92.56(7); C13–Pt–N2, 80.26(8).

**Figure 3:**
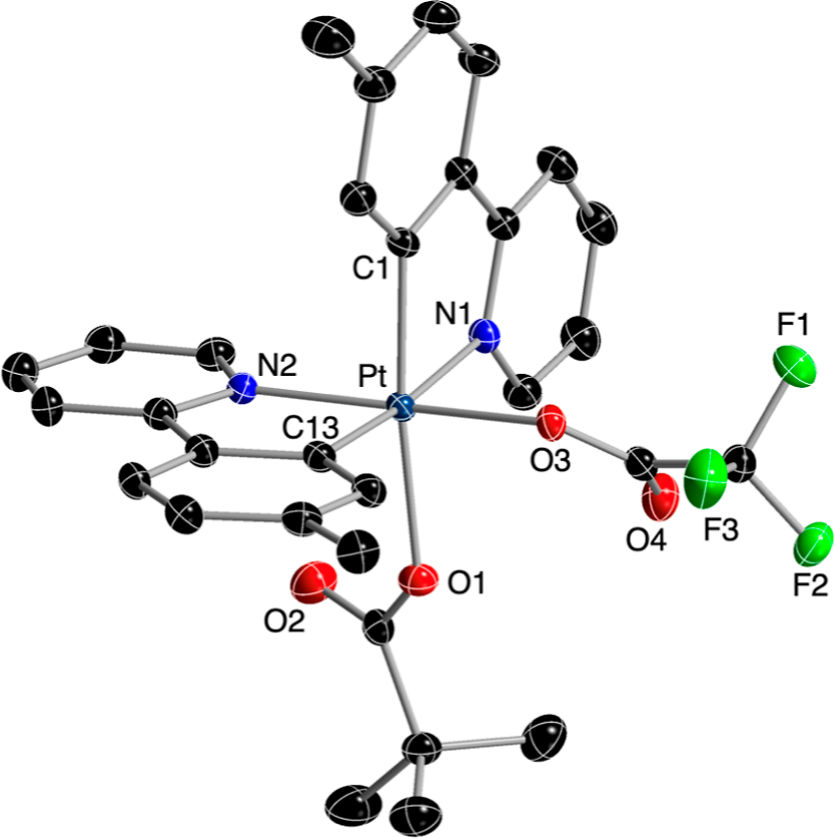
Structure of complex **8** in the crystal (thermal ellipsoids at 50% probability). Hydrogen atoms are omitted. Selected bond distances (Å) and angles (deg): Pt–C13, 2.002(2); Pt–N2, 2.0080(18); Pt–C1, 2.022(2); Pt–O3, 2.0349(15); Pt–O1, 2.1257(16); Pt–N1, 2.1514(19); C1–Pt–N1, 80.18(8); N2–Pt–C1, 89.59(8); C13–Pt–N2, 81.37(8); C13–Pt–O1, 87.74(8); O3–Pt–O1, 86.42(6); O3–Pt–N1, 80.18(8).

The crystal structures of complexes **5** and **8** were solved by X-ray diffraction and confirmed the proposed ligand arrangement (Figures 2 and 3). In both cases, the tpy ligands are unsymmetrically disposed. The acetato ligand in **5** and the trifluoroacetato in **8** are situated trans to the coordinated nitrogen of one of the tpy ligands, whereas the pivalato ligand is trans to the metalated carbon of the other tpy ligand. The corresponding Pt–O bond distances are ca. 0.1 Å longer for the pivalato relative to the acetato or trifluoroacetato, because of the stronger trans influence of the metalated aryl ring.

### Electronic Absorption Spectra

**Figure 4:**
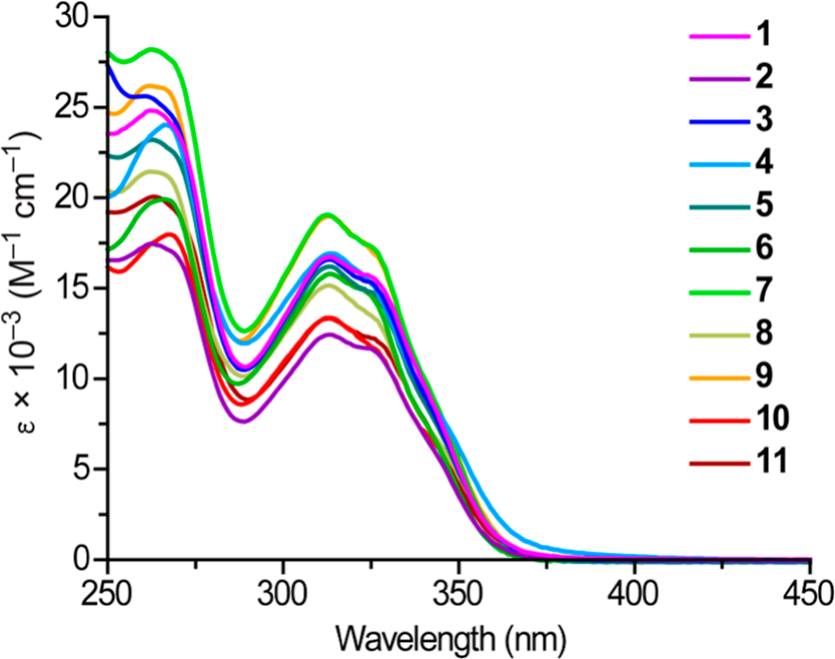
Electronic absorption spectra of **1**–**11** in MeCN solution (ca. 5 × 10^–5^ M) at 298 K.

The electronic absorption spectra of complexes **1**–**11** in MeCN solution are shown in Figure 4 and the data are compiled in Table 1. All of them show a vibronically structured band in the 290–360 nm range, which can be assigned to the lowest-energy singlet π–π* transition centered on the cyclometalated tpy ligands (^1^LC). Its shape is characteristic of bis-cyclometalated Pt(IV) complexes with an unsymmetrical arrangement of cyclometalated 2-arylpyridines^27,35,36^ and does not show a perceptible dependency on the electronic properties of the carboxylato ligands. Additionally, some of the derivatives give rise to a weak tail extending to ca. 400 nm, which can be mainly ascribed to ligand-to-ligand charge transfer (^1^LLCT) transitions, as deduced from TDDFT calculations (see below).

**Table 1:** Electronic Absorption Data for the Dicarboxylato Complexes in MeCN Solution (Ca. 5 × 10^–5^ M) at 298 K

complex	λ_max_/nm (ε × 10–3/M^–1^ cm^–1^)
**1**	263 (24.8), 313 (16.7), 327 (15.3), 341 (9.3)
**2**	266 (17.3), 313 (12.4), 327 (11.5), 341 (6.8)
**3**	263 (25.5), 313 (16.5), 327 (14.9), 343 (8.0)
**4**	266 (24.0), 314 (16.9), 326 (15.1), 349 (6.5)
**5**	263 (23.2), 313 (16.2), 325(14.8), 341 (8.2)
**6**	267 (19.9), 313 (15.8), 324(17.2), 339 (8.0)
**7**	263 (28.2), 312 (19.1), 325(17.3), 341 (9.9)
**8**	262 (21.4), 313 (15.2), 325(13.5), 346 (6.1)
**9**	262 (26.2), 313 (18.9), 324(14.7), 341 (9.5)
**10**	267 (18.0), 312 (13.5), 327(11.5), 344 (6.0)
**11**	263 (20.6), 313 (13.4), 328 (11.9), 341 (7.4)

### Photoreactivity

**Scheme 3:**
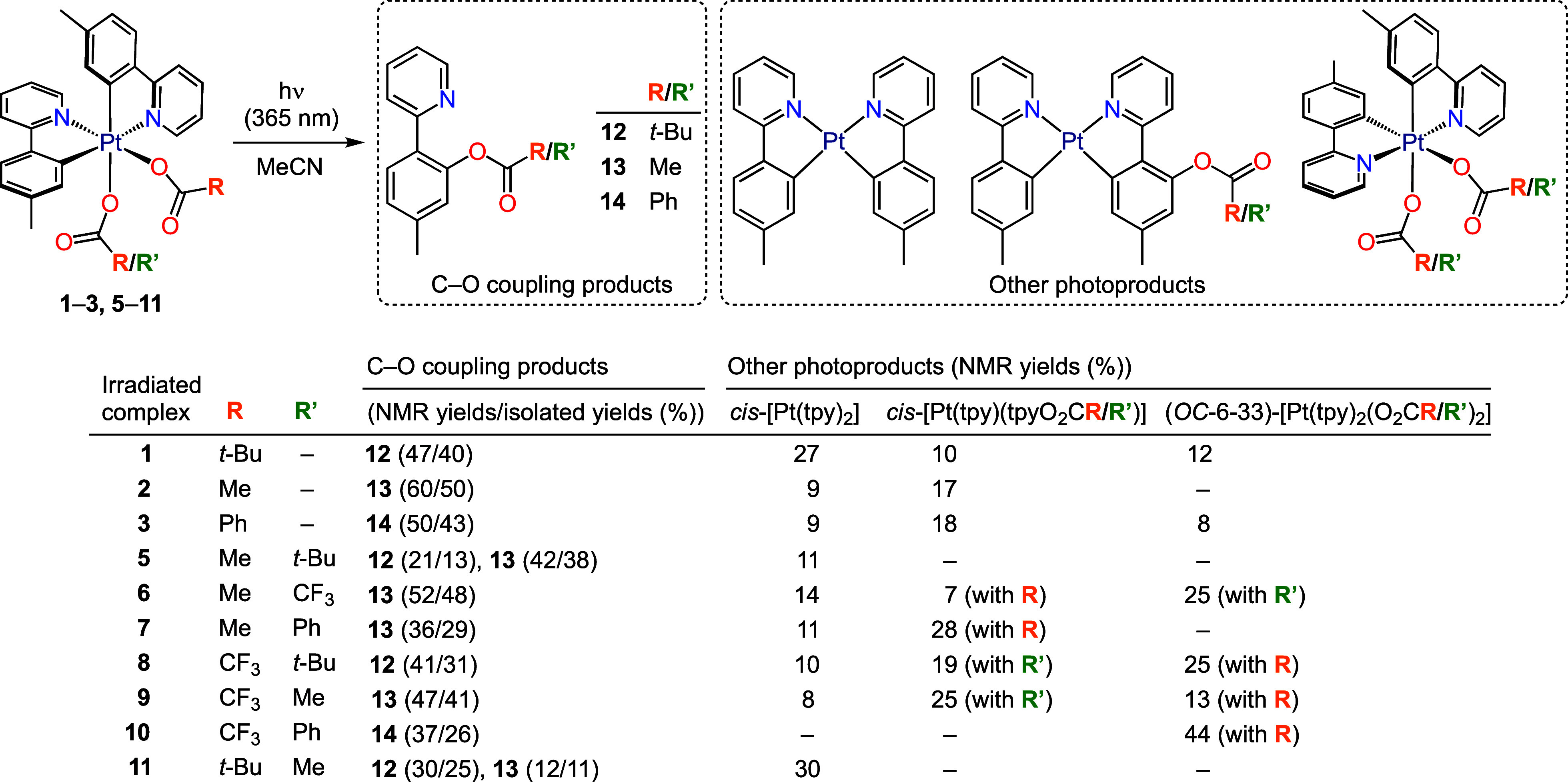
Identified Photoproducts from the Irradiation of **1**–**3**, **5**–**11** with UV Light

**Scheme 4:**
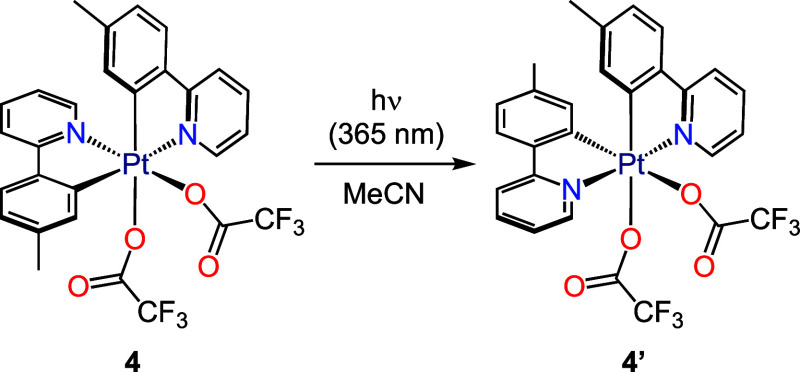
Photoisomerization of Complex **4** Under UV Light Irradiation

To characterize the photochemical reactivity of the dicarboxylato complexes, we irradiated them in deoxygenated MeCN solution at room temperature using a 365 nm LED source. The complexes were completely consumed within 2 h. For most of complexes **1**–**3** and **5**–**11**, the major product was 5-methyl-2-(2-pyridyl)phenyl pivalate (**12**), 5-methyl-2-(2-pyridyl)phenyl acetate (**13**) or 5-methyl-2-(2-pyridyl)phenyl benzoate (**14**), which result from the reductive coupling between a tpy ligand and a carboxylato ligand (Scheme 3). These products formed in up to 60% yield, as determined from ^1^H NMR spectra of the crude reaction mixtures using an internal standard, and could be isolated in up to 50% yields through chromatography. Additional photoproducts were detected in the crude mixtures after irradiation and their yields were determined by ^1^H NMR; depending on the electronic properties of the carboxylato ligands, these include different proportions of *cis*-[Pt(tpy)_2_], *cis*-[Pt(tpy)(tpyO_2_CR/R′)] (where tpyO_2_CR/R′ is a cyclometalated 5-methyl-2-(2-pyridyl)phenyl carboxylate) or a *C*_2_-symmetrical complex with two identical carboxylato ligands (*OC*-6-33)-[Pt(tpy)_2_(O_2_CR/R′)_2_], as indicated in Scheme 3. The bis(trifluoroacetato) complex **4** underwent photoisomerization to (*OC*-6-33)-[Pt(tpy)_2_(O_2_CCF_3_)_2_] (**4′**; 60% isolated yield) (Scheme 4), as observed for the reported ppy complex (*OC*-6-32)-[Pt(ppy)_2_(O_2_CCF_3_)_2_],^35^ and no other photoproducts were detected.

**Scheme 5:**
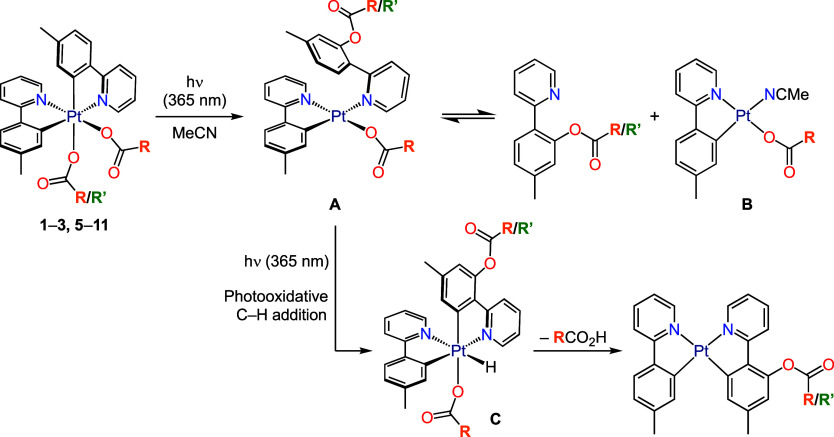
Hypothesized Reaction Pathway and Intermediates Leading to *cis*-[Pt(tpy)(tpyO_2_CR/R′)]

Complexes **1**–**3** produced relatively good yields of the respective C–O coupling products (**12**–**14**), together with significant amounts of *cis*-[Pt(tpy)_2_], the corresponding *cis*-[Pt(tpy)(tpyO_2_CR)] complex and, in the cases of **1** and **3**, the isomer (*OC*-6-33)-[Pt(tpy)_2_(O_2_CR)_2_]. The formation of *cis*-[Pt(tpy)_2_] reveals a competitive pathway through which both carboxylato ligands are eliminated, although the ensuing organic species was not detected. Within this triad of complexes, the highest proportion of *cis*-[Pt(tpy)_2_] is obtained for **1**, suggesting that the C–O coupling is relatively difficult for the pivalato ligand, which can be attributed to the steric hindrance of the *t*-Bu group. Complexes *cis*-[Pt(tpy)(tpyO_2_CR)] result from the cyclometalation of **12**–**14**, and therefore they should be accounted for when estimating the total yield of the C–O coupling process, which reaches its highest value for **2** (77%, as determined from ^1^H NMR data). A possible explanation for the formation of these complexes is that the reductive C–O coupling step produces a Pt(II) intermediate bearing compounds **12**–**14** as *N*-coordinated ligands (**A**, Scheme 5), from which they can be released, with formation of the Pt(II) complex **B** or a similar species that could be in equilibrium with **A**. We note that Pt(II) complexes of the type [Pt(C∧N)(O_2_CR)(L)] have been previously demonstrated as the products of thermal reductive eliminations from Pt(IV) complexes.^41^ Coordination equilibria involving cyclometalated Pt(II) complexes and 2-arylpyridines have also been demonstrated.^36^ The coordinated tpyO_2_CR ligand in **A** could undergo metalation through a photooxidative C–H addition mechanism, which has been demonstrated for complexes of the type [PtX(C∧N)(N∧CH)] (X = Me, C_6_F_5_, Cl)^48−50^ and would produce a hydride intermediate (**C**), that would undergo the reductive elimination of a carboxylic acid molecule. When the irradiation of **2** was conducted in the presence of **13** (molar ratio: 1:2), complex [Pt(tpy)(tpyO_2_CMe)] (**15**) was obtained in a much higher yield (51%), which is consistent with the proposed pathway because a higher concentration of **13** should shift the **A**–**B** equilibrium toward **A**, thereby increasing the probability of cyclometalation. However, intermediates **A**–**C** could not be identified in the reaction mixture. Although the derivatives *cis*-[Pt(tpy)(tpyO_2_CR)] with R = *t*-Bu or Ph were not isolated, they were identified in the crude reaction mixtures based on the characteristic ^1^H NMR resonances of the tpyO_2_CR ligands.

Significant photoisomerization to the *C*_2_-symmetrical complexes (*OC*-6-33)-[Pt(tpy)_2_(O_2_CR)_2_] was observed for **1** and **3** but not for **2**. Although these isomers could not be isolated, they were unequivocally identified by their characteristic single set of aromatic resonances in the ^1^H NMR spectra^35^ of the crude reaction mixtures. In the case of **1**, it is likely that this photoisomerization pathway becomes competitive because of the difficulty of the C–O coupling due to steric factors. However, in the case of **3**, the effect could be a consequence of the weaker electron-donating character of the benzoato ligand, which would also make the C–O coupling more difficult and favor isomerization, as deduced from the behavior of the bis-trifluoroacetato complex **4**.

As observed for **1**–**3**, the main photoprocess taking place from **5** and its isomer **11** is the C–O reductive coupling, followed by reduction to *cis*-[Pt(tpy)_2_]. However, complexes *cis*-[Pt(tpy)(tpyO_2_CMe/*t*-Bu)] and (*OC*-6-33)-[Pt(tpy)_2_(O_2_CMe/*t*-Bu)_2_] were not detected. Compounds **12** and **13** were obtained as coupling products in both cases, but **13** was obtained in a higher proportion from **5**, whereas **12** was produced in a higher proportion from **11**, meaning that the C–O coupling is favored for the carboxylato trans to N as compared to the one trans to C. In addition, the proportion of *cis*-[Pt(tpy)_2_] produced from **11** is significantly higher compared to **5**, but similar to that produced from **1**, in line with the previous observation that the C–O reductive coupling involving the pivalate is more difficult as compared to the acetate.

Complexes **6**–**10** produced a single C–O coupling product involving the strongest electron-donating carboxylato ligand. In no case was a coupling product involving the trifluoroacetato ligand observed. Among the additional photoproducts, the corresponding *cis*-[Pt(tpy)(tpyO_2_CR/R′)] complexes were obtained in significant proportions for **6**–**9** and the total C–O coupling yields range from 59 to 72%. The trifluoroacetato derivatives **6**, **8** and **9** produced significant amounts of the *C*_2_-symmetrical complex **4′**, indicating that ligand exchange and isomerization are taking place. These are in fact the major processes in the case of **10**, which produces **4′** in 44% yield, consistent with the above-noted observation that the less electron-donating carboxylato ligands favor isomerization.

### Computational Study

**Figure 5:**
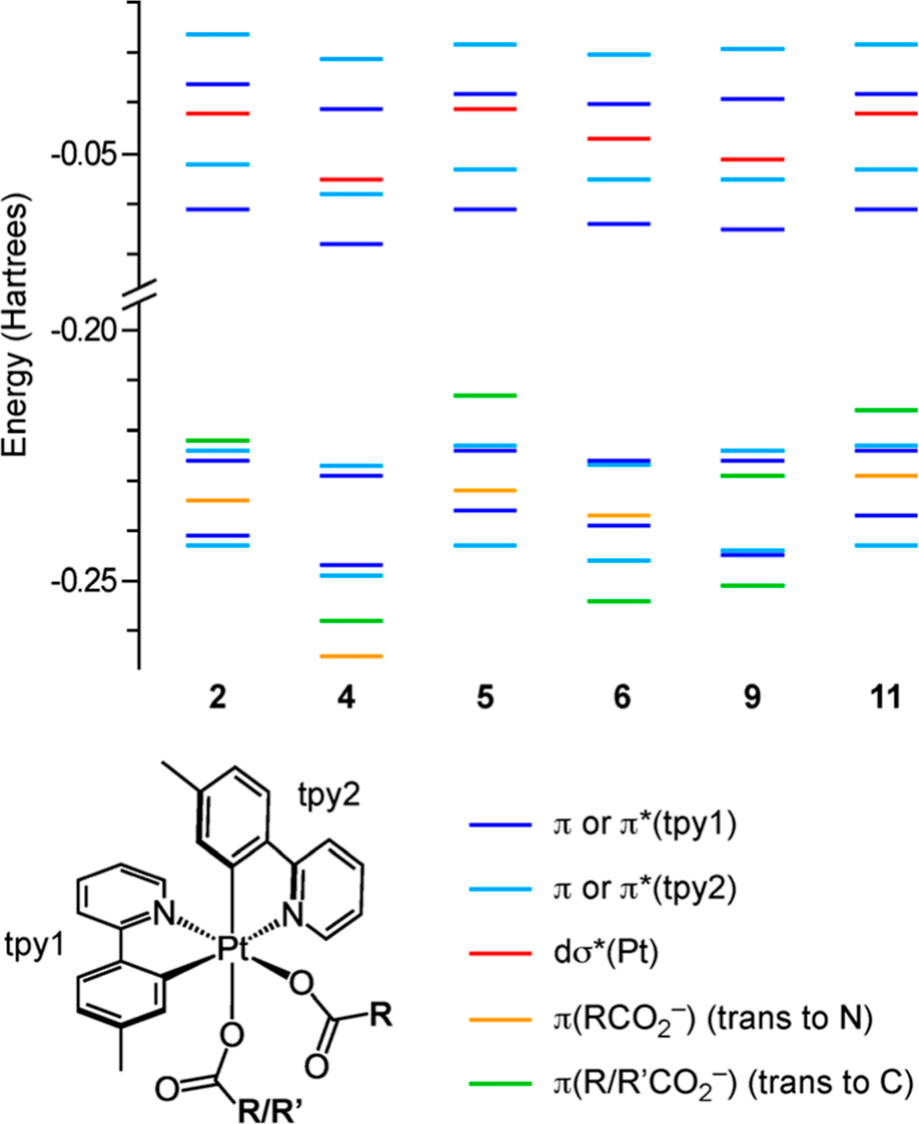
Molecular orbital energy diagram from DFT calculations.

To get an understanding of the photochemical reactivity of the dicarboxylato complexes, DFT and TDDFT calculations were carried out for complexes **2**, **4**, **5**, **6**, **9** and **11** considering solvent effects (MeCN). The complete details are given in the Supporting Information. A frontier orbital energy diagram and a color-coded representation of the main contributions are shown in Figure 5. The highest occupied molecular orbital (HOMO) of complexes **4**, **6** and **9** is mainly a π orbital of one of the tpy ligands (tpy2 in Figure 5), with little participation from metal dπ orbitals. As expected, the energy of the π system of the carboxylato ligands depends on the electronic properties of the R or R′ group and is significantly lower for the trifluoroacetato as compared to the acetato or pivalato ligands. In addition, it is affected by the coordination position, being higher when the carboxylato is trans to a metalated tolyl group than when it is trans to a pyridyl group. Thus, the π system of the carboxylato trans to a tolyl group becomes the HOMO in complexes **2** (acetato), **5** (pivalato) and **11** (acetato). In all cases, the lowest unoccupied molecular orbital (LUMO) and the LUMO+1 are essentially π* orbitals of the tpy ligands. The LUMO+2 is a dσ* orbital mainly distributed along the N–Pt–O axis and its energy is lower for derivatives that have a trifluoroacetato ligand in this axis (**4**, **9**).

**Figure 6:**
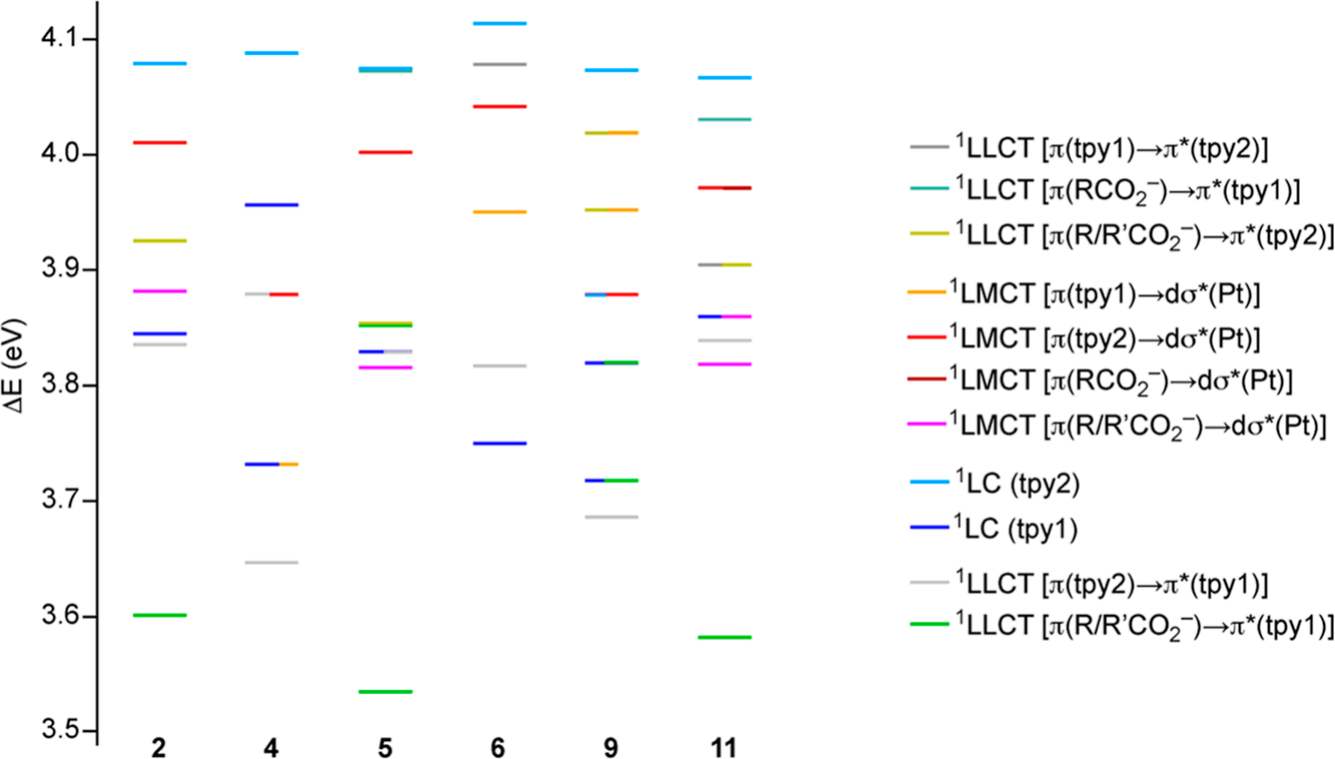
Energy diagram showing the lowest vertical singlet excitations for complexes **2**, **4**, **5**, **6**, **9**, and **11** from TDDFT calculations at the ground-state geometries and a color-coded representation of their main character (see Figure 5 for ligand numbering).

The TDDFT calculations show that the most important singlet excitations in the lowest-energy range correspond to essentially ^1^LC transitions involving each of the tpy ligands, which are predicted at around 320 (tpy1) or 304 (tpy2) nm (see Figure 6 for an energy diagram, Figure 5 for ligand numbering) and can be therefore associated with the most intense features observed at wavelengths longer than 300 nm in the experimental absorption spectra. Additional excitations with LLCT character and low oscillator strengths are also predicted, some of which have lower energies than the ^1^LC transitions and must contribute to the tails observed in the experimental spectra. In the cases of complexes **2**, **5** and **11**, the lowest of such excitations is a HOMO–LUMO transition, which can be designated as ^1^LLCT [π(R/R′CO_2_^–^) → π*(tpy1)]. Transitions of a similar character are higher in energy for **9** because the presence of the trifluoroacetato ligand causes a decrease in the energy of the acetato π system with respect to **2** or **11**, whereas they are not predicted among the lowest-energy excitations for **4** and **6** because these complexes have a trifluoroacetato ligand trans to a tolyl group. Instead, the lowest ^1^LLCT excitations in **4**, **6** and **9** occur between π and π* orbitals of different tpy ligands. Significant ^1^LMCT [π(R/R′CO_2_^–^) → dσ*(Pt)] excitations are predicted for **2**, **5** and **11**, whose energies are similar to those of the lowest ^1^LC(tpy1) excitation. In contrast, complexes **4**, **6** and **9** present important ^1^LMCT [π(tpy1 or tpy2) → dσ*(Pt)] excitations above the lowest ^1^LC(tpy1) excitation.

**Figure 7:**
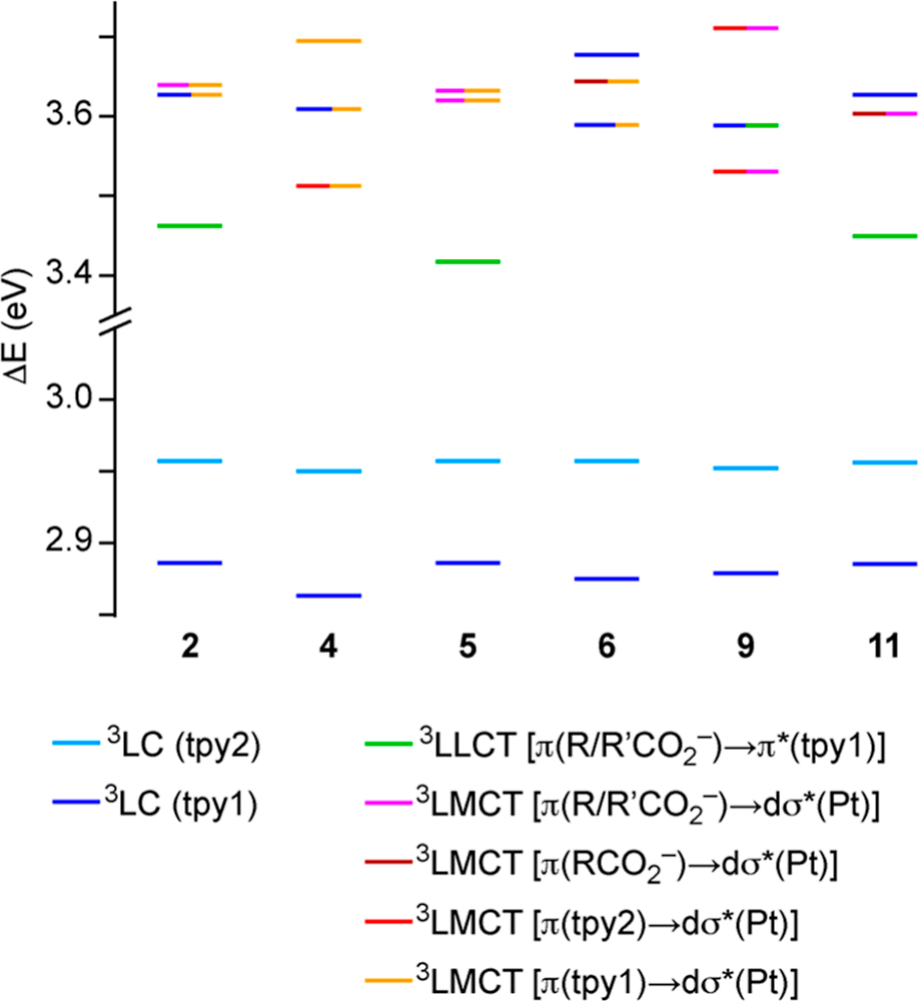
Energy diagram showing the lowest vertical triplet excitations for complexes **2**, **4**, **5**, **6**, **9**, and **11** from TDDFT calculations at the ground-state geometries and a color-coded representation of their main character (see Figure 5 for ligand numbering).

A diagram showing the energies of the first five vertical triplet excitations is depicted in Figure 7. In all cases, the two lowest triplet excitations (T_1_, T_2_) correspond to ^3^LC transitions involving each of the cyclometalated ligands. In the cases of **2**, **5** and **11**, the T_3_ excitation is a ^3^LLCT [π(R/R′CO_2_^–^) → π*(tpy)] transition. In most cases, the T_4_ and T_5_ excitations of these complexes and the T_3_–T_5_ excitations of **4**, **6** and **9** have a significant LMCT character from the carboxylato and/or tpy ligands to the lowest dσ* orbital.

### Proposed Reaction Pathways

The emission spectrum of the LED source (Figure S1) employed for the irradiation of the carboxylato complexes overlaps the lowest-energy edge of their absorption spectra up to ca. 340 nm. Therefore, the ^1^LLCT [π(R/R′CO_2_^–^) → π*(tpy1)] states for **2**, **5** and **11**, or ^1^LLCT [π(tpy2) → π*(tpy1)] for **4** and **9** should be populated, in addition to the lowest ^1^LC(tpy1) state in all cases. However, it cannot be stated with certainty that ^1^LMCT states are hit by this source for all of the complexes, because their energies are predicted to be higher than those of the ^1^LC(tpy1) state for some of them (**2**, **4**, **6**, **9**) and the shape of the corresponding bands cannot be distinguished in the experimental absorption spectra. Intersystem crossing to the triplet manifold should occur from the lowest singlet excited state due to the strong spin–orbit coupling induced by the metal, as commonly observed for cyclometalated Pt(IV) complexes, leading to the population of the lowest ^3^LC state.^27,29−31,33,34,36,51^ Such pathway has been previously demonstrated for the analogous complexes (*OC*-6-32)-[Pt(ppy)_2_(O_2_CR)_2_] (R = Me, CF_3_), which show phosphorescence from ^3^LC states at low temperature.^35^ Thermal population of triplet excited states of LMCT character could then cause the observed photoreactivity.

**Scheme 6:**
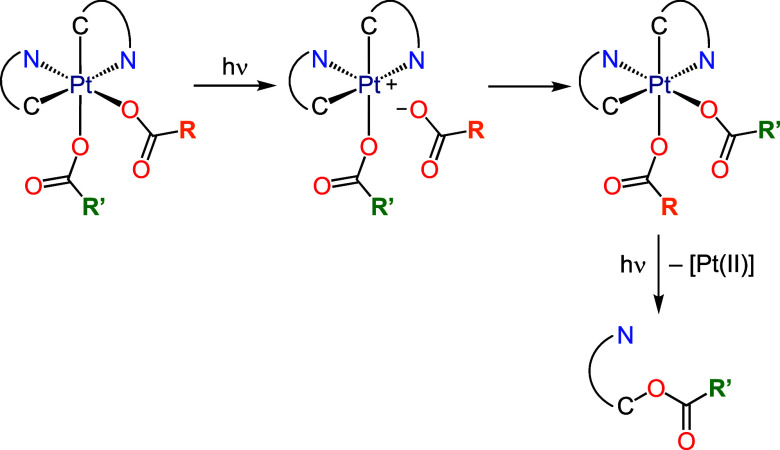
Proposed Heterolytic Dissociation of the Pt–O Bond Trans to N Leading to an Exchange of Coordination Positions Between Different Carboxylato Ligands

In all cases, LMCT states entail the occupation of the lowest dσ* orbital (LUMO+2), which is mainly distributed along the N–Pt–O axis and could cause the lengthening and cleavage of the involved N–Pt and/or Pt–O bonds. As previously deduced from the photoreactivity of **5** and **11**, which bear carboxylato ligands of similar electronic properties, the C–O coupling is much more likely to involve the carboxylato trans to N, suggesting that the primary effect of the lowest LMCT state is to trigger this process. Taking into account previous studies on the photoreactivity of hydroxo^23,24^ and carboxylato^22^ Pt(IV) complexes, it is possible that the LMCT state causes the homolytic cleavage of the Pt–O bond trans to N for the most electron-rich carboxylates, which could initiate the C–O coupling or, in a competitive pathway, the reduction to *cis*-[Pt(tpy)_2_]. However, radical-trapping experiments using 5,5-dimethyl-1-pyrroline *N*-oxide (DMPO) did not conclusively support the release of carboxyl radicals. Alternatively, these processes could take place through a concerted reductive elimination mechanism. The population of the lowest LMCT state could also trigger the heterolytic cleavage of the Pt–O bond trans to N for the trifluoroacetato ligand and, to a lesser extent, for other carboxylato ligands, which, in the cases of the mixed derivatives, could lead to an exchange between the coordination positions of the carboxylato ligands through a cationic pentacoordinate intermediate and subsequent C–O coupling involving the most electron-rich carboxylato (Scheme 6). The pentacoordinate intermediate could also facilitate the exchange of carboxylato ligands between the different Pt(IV) and Pt(II) species present in solution and promote a rearrangement of cyclometalated ligands, resulting in the formation of *C*_2_-symmetrical complexes with two identical carboxylato ligands, which become the only photostable Pt(IV) species. This would explain the high proportions of complex **4′** obtained from the irradiation of derivatives **6**, **8**–**10**.

## Conclusions

The photoreactivity of Pt(IV) complexes bearing two cyclometalated 2-(*p*-tolyl)pyridine ligands in an unsymmetrical disposition and two identical or different carboxylato ligands has been studied in detail. Three main photochemical processes have been demonstrated to take place upon irradiation with UV light, namely, C–O reductive coupling between one carboxylato and one cyclometalated ligand, reduction to a bis-cyclometalated Pt(II) complex, and isomerization to a *C*_2_-symmetrical Pt(IV) dicarboxylato complex. In addition, the cyclometalation of the ensuing C–O coupling products at a Pt(II) species is observed in most cases, which probably occurs photochemically. From the analysis of the proportions of photoproducts, three main generalities can be extracted: (i) the C–O coupling process is favored for the most electron-rich carboxylato ligands, whereas it is never observed for the trifluoroacetato ligand, which leads to isomerization; (ii) the C–O coupling preferentially involves the carboxylato ligand trans to N, and (iii) a bulky substituent on the carboxylato ligand makes the C–O coupling more difficult.

The computational study shows that the lowest LMCT state involves the occupation of a dσ* orbital mainly distributed along the N–Pt–O axis. This state could trigger the C–O coupling or reduction to the bis-cyclometalated Pt(II) complex for the most electron-rich carboxylato ligands through an initial homolytic Pt–O bond dissociation or a concerted reductive elimination mechanism. The cleavage of the Pt–O bond could occur exclusively in a heterolytic fashion for the trifluoroacetato ligand and marginally for the rest of carboxylato ligands, leading to scrambling and providing a pathway for the C–O coupling involving the carboxylato originally trans to C.

In short, the present study provides an understanding of the factors that facilitate light-induced C(sp^2^)–O reductive couplings in the coordination sphere of Pt(IV). This new knowledge could be of application for the development of photochemical catalyzed processes leading to C–heteroatom couplings using cyclometalated platinum complexes as catalysts.

## Experimental Section

### General Considerations, Materials and Instrumentation

Unless otherwise noted, all photochemical reactions were carried out at room temperature using extra-dry MeCN as the solvent and flame-dried glassware under an N_2_ atmosphere. Synthesis grade Et_2_O and CH_2_Cl_2_ were degassed and dried using a Pure Solv MD-5 solvent purification system from Innovative Technologies, Inc. Other solvents were used as received. Complexes *cis*-[Pt(tpy)_2_]^48^ and (*OC*-6-32)-[Pt(tpy)_2_(OCCR)_2_] [R = Me (**2**), CF_3_ (**4**)]^37^ were synthesized following published procedures. Compound **13** was independently prepared following a published method.^52^ All other reagents were obtained from commercial sources. Irradiations with 365 nm UV light were performed in flat-bottom, 20 mL Carius tubes (⌀ 20 mm) made of borosilicate glass and fitted with a PTFE vacuum stopcock, which were placed on top of individual LED emitters (LED Engin LuxiGen LZ1–10UV0R-0000) fixed to an aluminum heat sink and cooled by a fan. The radiant flux at the bottom of the tube was ca. 1360 mW based on technical specifications. The emission spectrum of the LEDs is shown in Figure S1. NMR spectra were recorded on Bruker Avance 300, 400, or 600 MHz spectrometers at 298 K. ^1^H and ^13^C{^1^H} NMR spectra were referenced using residual signals of nondeuterated solvent and are given in ppm downfield from tetramethylsilane. ^19^F NMR spectra were referenced against external CFCl_3_. Elemental analyses were carried out with a LECO CHNS-932 microanalyzer. UV–vis absorption spectra were registered on a PerkinElmer Lambda 750S spectrophotometer. Electrospray ionization high-resolution mass spectra (ESI-HRMS) were recorded on an Agilent 6220 Accurate-Mass time-of-flight (TOF) LC/MS.

### Synthesis of (*OC*-6-32)-[Pt(tpy)_2_(O_2_C-*t-*Bu)_2_] (**1**)

To a suspension of *cis*-[Pt(tpy)_2_] (100 mg, 0.19 mmol) in acetone (2 mL) was added PhI(O_2_C-*t-*Bu)_2_ (114 mg, 0.28 mmol) and the mixture was stirred for 1 h, whereupon the color of the suspension changed from yellow to white. Et_2_O (10 mL) and *n*-pentane (3 mL) were then added to complete the precipitation of the white solid, which was collected by filtration, washed with Et_2_O (3 × 2 mL) and vacuum-dried to give **1**. Yield: 110 mg, 80%. ^1^H NMR (600 MHz, CD_2_Cl_2_, δ): 9.22 (ddd, *J* = 5.5, 1.7, 0.9 Hz, 1H), 8.07–8.02 (m, 1H), 7.99 (d, *J* = 7.7 Hz, 1H), 7.72 (s with satellites, *J*_PtH_ = 10.4 Hz, 1H), 7.71–7.67 (m, 2H), 7.53 (dd, *J* = 11.7, 7.8 Hz, 2H), 7.52–7.49 (m, 1H), 7.38 (ddd with satellites, *J*_PtH_ = 31.3 Hz, *J*_HH_ = 6.1, 1.4, 0.8 Hz, 1H), 7.21 (ddd, *J* = 7.8, 1.7, 0.6 Hz, 1H), 6.85–6.78 (m, 2H), 6.10 (s with satellites, *J*_PtH_ = 39.2 Hz, 1H), 2.54 (s, 3H), 2.03 (s, 3H), 1.03 (s, 9H), 0.92 (s, 9H). ^13^C NMR (151 MHz, CD_2_Cl_2_, δ): 183.6 (C), 182.2 (C), 166.4 (C), 162.5 (C), 149.2 (CH), 148.1 (CH), 146.4 (C), 142.3 (*J*_PtC_ = 29.5 Hz, C), 141.2 (*J*_PtC_ = 47.3 Hz, C), 140.4 (CH), 140.2 (CH), 140.1 (C), 139.3 (C), 133.7 (*J*_PtC_ = 33.0 Hz, CH), 132.1 (C), 129.7 (CH), 126.9 (CH), 126.6 (CH), 124.7 (CH), 124.5 (*J*_PtC_ = 35.4 Hz, CH), 123.2 (CH), 122.2 (*J*_PtC_ = 29.2 Hz, CH), 119.8 (CH), 119.7 (CH), 40.3 (C), 39.8 (C), 28.7 (3CH_3_), 28.6 (3CH_3_), 22.5 (CH_3_), 21.9 (CH_3_). Anal. Calcd for C_34_H_38_N_2_O_4_Pt: C, 55.65; H, 5.21; N, 3.82. Found: C, 55.83; H, 5.06; N, 3.55.

### Synthesis of (*OC*-6-32)-[Pt(tpy)_2_(O_2_CPh)_2_] (**3**)

This complex was obtained as a white solid following the procedure described for **1**, from *cis*-[Pt(tpy)_2_] (110 mg, 0.21 mmol) and PhI(O_2_CPh)_2_ (140 mg, 0.31 mmol). Yield: 125 mg, 78%. ^1^H NMR (600 MHz, CD_2_Cl_2_, δ): 9.40 (d, *J* = 5.4 Hz, 1H), 8.13–8.01 (m, 2H), 8.01–7.94 (m, 2H), 7.90 (s with satellites, *J*_PtH_ = 20.8, 1H), 7.88–7.82 (m, 2H), 7.80–7.71 (m, 2H), 7.61–7.56 (m, 2H), 7.54 (d, *J* = 6.0 Hz, 1H), 7.52–7.48 (m, 1H), 7.42–7.38 (m, 1H), 7.36–7.29 (m, 3H), 7.29–7.20 (m, 3H), 6.93–6.86 (m, 2H), 6.19 (s with satellites, *J*_PtH_ = 37.6 Hz, 1H), 2.55 (s, 3H), 2.06 (s, 3H). ^13^C NMR (151 MHz, CD_2_Cl_2_, δ): 171.6 (C), 170.2 (C), 166.5 (C), 162.2 (C), 150.1 (CH), 148.5 (CH), 146.2 (C), 143.0 (*J*_PtC_ = 29.0 Hz), 141.6 (*J*_PtC_ = 46 Hz, C), 140.7 (CH), 140.6 (CH), 140.0 (*J*_PtC_ = 17.0 Hz, C), 139.3 (C), 138.1 (C), 135.7 (C), 133.7 (*J*_PtC_ = 32.8 Hz, 1H), 131.1 (CH), 131.0 (C), 130.6 (CH), 130.1 (CH), 130.0 (CH), 129.8 (CH), 128.2 (CH), 128.1 (CH), 127.5 (CH), 127.1 (CH), 125.0 (CH), 124.9 (CH), 123.5 (CH), 122.5 (*J*_PtC_ = 28.0 Hz, CH), 120.1 (CH), 120.0 (CH), 22.7 (CH_3_), 22.0 (CH_3_). Anal. Calcd for C_31_H_29_N_2_O_4_Pt: C, 58.99; H, 3.91; N, 3.62. Found: C, 59.07; H, 3.96; N, 3.34.

### Synthesis of (*OC*-6-43)-[Pt(tpy)_2_(O_2_CMe)(O_2_C-*t*-Bu)] (**5**)

To a solution of **2** (100 mg, 0.15 mmol) in CH_2_Cl_2_ (2 mL) were added pivalic acid (44 mg, 0.43 mmol) and Na_2_CO_3_ (100 mg, 0.94 mmol), and the mixture was stirred for 20 h protected from light. The suspension was filtered through Celite and the solvent was removed under reduced pressure. The addition of Et_2_O (5 mL) to the residue led to the precipitation of a white solid, which was collected by filtration, washed with Et_2_O (2 × 3 mL) and vacuum-dried to give **5**. Yield: 81 mg, 75%. ^1^H NMR (600 MHz, CD_2_Cl_2_, δ): 9.39 (ddd, *J* = 5.5, 1.7, 0.8 Hz, 1H), 8.06–8.02 (m, 1H), 7.98 (d, *J* = 8.2 Hz, 1H), 7.72–7.67 (m, 3H), 7.55–7.49 (m, 4H), 7.42 (ddd with satellites, *J*_PtH_ = 31.8 Hz, *J*_HH_ = 6.1, 1.3, 0.9 Hz, 1H), 7.22 (ddd, *J* = 7.9, 1.6, 0.7 Hz, 1H), 6.85 (ddd, *J* = 7.9, 1.7, 0.8 Hz, 1H), 6.82 (dt, *J* = 6.3, 2.5 Hz, 1H), 6.10 (s with satellites, *J*_PtH_ = 38.0 Hz, 1H), 2.57 (s, 3H), 2.03 (s, 3H), 1.90 (s, 3H), 0.87 (s, 9H). ^13^C{^1^H} NMR (150.8 MHz, CD_2_Cl_2_): 183.6 (C), 174.8 (C), 166.3 (C), 162.2 (*J*_PtC_ = 41.8 Hz, C), 150.9 (CH), 148.4 (CH), 146.5 (C), 142.6 (*J*_PtC_ = 28.6 Hz, C), 141.3 (*J*_PtC_ = 48.5 Hz, C), 140.4 (CH), 140.3 (CH), 140.2 (C), 139.0 (C), 133.8 (*J*_PtC_ = 35.4 Hz, CH), 131.5 (C), 129.5 (CH), 127.1 (CH), 126.9 (CH), 124.8 (*J*_PtC_ = 26.4 Hz, CH), 124.6 (*J*_PtC_ = 33.3 Hz, CH), 123.1 (CH), 122.2 (*J*_PtC_ = 25.7 Hz, CH), 119.7 (*J*_PtC_ = 38.9 Hz, CH), 119.6 (CH), 40.4 (C), 28.6 (3CH_3_), 24.1 (*J*_PtC_ = 46.6 Hz, CH_3_), 22.6 (CH_3_), 21.9 (CH_3_). Anal. Calcd for C_31_H_32_N_2_O_4_Pt: C, 53.83; H, 4.66; N, 4.05. Found: C, 53.55; H, 4.87; N, 4.01.

### Synthesis of (*OC*-6-43)-[Pt(tpy)_2_(O_2_CMe)(O_2_CCF_3_)] (**6**)

This complex was obtained as a white solid following the procedure described for **5**, but heating at 40 °C, from **2** (52 mg, 0.08 mmol) trifluoroacetic acid (100 μL, 1.30 mmol) and Na_2_CO_3_ (100 mg, 0.94 mmol). Yield: 50 mg, 89%. ^1^H NMR (401 MHz, CD_2_Cl_2_, δ): 9.21 (d, *J* = 4.5 Hz, 1H), 8.13–8.05 (m, 1H), 8.05–8.00 (m, 1H), 7.80–7.75 (m, 2H), 7.72 (s with satellites, *J*_PtH_ = 20.1 Hz, 1H), 7.61–7.54 (m, 2H), 7.52 (d, *J* = 8.0 Hz, 1H), 7.36 (d with satellites, *J*_PtH_ = 31.7 Hz, *J*_HH_ = 6.1 Hz, 1H), 7.28 (d, *J* = 7.8 Hz, 1H), 6.94–6.84 (m, 2H), 6.06 (s with satellites, *J*_PtH_ = 43.9 Hz, 1H), 2.58 (s, 3H), 2.05 (s, 3H), 1.92 (s, 3H). ^19^F NMR (377 MHz, CD_2_Cl_2_, δ): –75.34 (br). ^13^C{^1^H} NMR (151 MHz, CD_2_Cl_2_, δ): 166.5 (C), 161.5 (q, *J*_CF_ = 33.0 Hz, C), 161.4 (C), 149.7 (CH), 147.9 (CH), 145.4 (C), 143.3 (C), 141.8 (C), 141.1 (CH), 140.9 (CH),139.2 (C), 138.9 (C), 133.2 (*J*_PtC_ = 36.0 Hz, CH), 130.0 (CH), 128.2 (C), 128.0 (CH), 127.5 (CH), 125.0 (CH), 123.8 (CH), 123.0 (CH), 120.4 (CH), 120.1 (CH), 116.9 (q, *J*_CF_ = 294.2 Hz, CF_3_), 23.8 (CH_3_), 22.6 (CH_3_), 21.9 (CH_3_). Anal. Calcd for C_28_H_23_F_3_N_2_O_4_Pt: C, 47.80; H, 3.30; N, 3.98. Found: C, 47.84; H, 3.47; N, 3.83.

### Synthesis of (*OC*-6-43)-[Pt(tpy)_2_(O_2_CMe)(O_2_CPh)] (**7**)

This complex was obtained as a white solid following the procedure described for **5**, but heating at 40 °C, from **2** (60 mg, 0.09 mmol) benzoic acid (100 mg, 0.82 mmol) and Na_2_CO_3_ (100 mg, 0.94 mmol). Yield: 51 mg, 78%. ^1^H NMR (600 MHz, CD_2_Cl_2_, δ): 9.38 (ddd, *J* = 5.5, 1.6, 0.8 Hz, 1H), 8.07–8.03 (m, 1H), 8.01 (d, *J* = 8.0 Hz, 1H), 7.83–7.80 (m, 2H), 7.78 (s *J*_PtH_ = 20.0 Hz, 1H), 7.75–7.71 (m, 2H), 7.57 (d, *J* = 7.9 Hz, 2H), 7.55 (d, *J* = 7.8 Hz, 1H), 7.51–7.55 (m, 2H), 7.36–7.31 (m, 1H), 7.29–7.22 (m, 3H), 6.90–6.83 (m, 2H), 6.12 (s with satellites, *J*_PtH_ = 38.5 Hz, 1H), 2.58 (s, 3H), 2.05 (s, 3H), 1.96 (s, 3H). ^13^C{^1^H} NMR (151 MHz, CD_2_Cl_2_, δ): 175.0 (*J*_PtC_ = 32.7 Hz, C), 171.6 (C), 166.4 (*J*_PtC_ = 33.6 Hz, C), 162.0 (*J*_PtC_ = 41.6 Hz, C), 150.5 (CH), 148.3 (CH), 146.0 (C), 142.9 (*J*_PtC_ = 29.4 Hz, C), 141.5 (*J*_PtC_ = 53.2 Hz, C), 140.5 (CH), 140.4 (C), 140.0 (C),139.2 (*J*_PtC_ = 31.1 Hz, C), 138.0 (C), 133.5 (*J*_PtC_ = 36.1 Hz, CH), 130.8 (C), 130.6 (CH), 129.9 (CH), 129.6 (CH), 128.0 (CH), 127.5 (CH), 127.0 (CH), 125.0 (CH), 124.9 (*J*_PtC_ = 30.8 Hz, CH), 124.8 (*J*_PtC_ = 21.2 Hz, CH), 123.3 (CH), 122.4 (*J*_PtC_ = 29.0 Hz, CH), 120.0 (*J*_PtC_ = 36.3 Hz, CH), 119.8 (CH), 24.0 (CH_3_), 22.6 (CH_3_), 21.9 (CH_3_). Anal. Calcd for C_33_H_28_N_2_O_4_Pt: C, 55.69; H, 3.97; N, 3.94. Found: C, 55.71; H, 4.24; N, 3.92.

### Synthesis of (*OC*-6-34)-[Pt(tpy)_2_(O_2_CCF_3_)(O_2_C-*t*-Bu)] (**8**)

This complex was obtained as a white solid following the procedure described for **5**, from **4** (100 mg, 0.13 mmol), pivalic acid (100 mg, 0.98 mmol) and Na_2_CO_3_ (140 mg, 0.31 mmol). Yield: 85 mg, 86%. ^1^H NMR (401 MHz, CD_2_Cl_2_, δ): 9.37 (d, *J* = 5.1 Hz, 1H), 8.13–8.06 (m, 1H), 8.06–8.00 (m, 1H), 7.76–7.71 (m, 2H), 7.63 (s with satellites, *J*_PtH_ = 18.6 Hz, 1H), 7.60–7.52 (m, 3H), 7.42 (d with satellites, *J*_PtH_ = 38.4 Hz, *J*_HH_ = 6.0 Hz, 1H), 7.25 (d, *J* = 7.8 Hz, 1H), 6.90 (d, *J* = 7.8 Hz, 1H), 6.88–6.81 (m, 1H), 6.13 (s with satellites, *J*_PtH_ = 35.8 Hz, 1H), 2.55 (s, 3H), 2.06 (s, 3H), 0.89 (s, 9H). ^19^F NMR (282.4 MHz, CD_2_Cl_2_, δ): – 75.32 (s, *J*_PtF_ = 10.6 Hz). ^13^C{^1^H} NMR (150.8 MHz, CD_2_Cl_2_, δ): 183.8 (C), 166.8 (C), 161.9 (C), 160.9 (q, *J*_CF_ = 35.8 Hz, C), 150.8 (CH), 148.3 (CH), 146.2 (C), 143.1 (C), 142.0 (C), 141.0 (CH), 140.7 (CH), 139.9 (C), 138.7 (*J*_PtC_ = 32.2 Hz, C), 134.0 (*J*_PtC_ = 30.9 Hz, CH), 130.7 (C), 129.0 (CH), 127.6 (CH), 127.5 (CH), 125.2 (*J*_PtC_ = 24.3 Hz, CH), 124.8 (*J*_PtC_ = 30.4 Hz, CH), 122.5 (*J*_PtC_ = 32.8 Hz, CH), 120.1 (CH), 40.4 (C), 28.5 (3CH_3_), 22.7 (CH_3_), 22.0 (CH_3_). Anal. Calcd for C_31_H_29_N_2_O_4_Pt: C, 49.93; H, 3.92; N, 3.76. Found: C, 49.97; H, 4.17; N, 3.60.

### Synthesis of (*OC*-6-34)-[Pt(tpy)_2_(O_2_CCF_3_)(O_2_CMe)] (**9**)

This complex was obtained as a white solid following the procedure described for **5**, from **4** (100 mg, 0.132 mmol), acetic acid (500 μL, 8.70 mmol) and Na_2_CO_3_ (50 mg, 0.47 mmol). Yield: 67 mg, 72%. ^1^H NMR (600 MHz, CD_2_Cl_2_, δ): 9.31 (ddd, *J* = 5.6, 1.7, 0.8 Hz, 1H), 8.12–8.07 (m, 1H), 8.06–8.01 (m, 1H), 7.75–7.70 (m, 2H), 7.60 (s with satellites, *J*_PtH_ = 18.3 Hz, 1H), 7.58–7.53 (m, 4H), 7.48 (d with satellites, *J*_PtH_ = 37.2 Hz, *J*_HH_ = 6.5 Hz, 1H), 7.28 (d, *J* = 7.9 Hz, 1H), 6.89 (d, *J* = 8.0 Hz, 1H), 6.84 (dt, *J* = 6.4, 2.5 Hz, 1H), 6.07 (s with satellites, *J*_PtH_ = 36.1 Hz, 1H), 2.56 (s, 3H), 2.04 (s, 3H), 1.82 (s, 3H). ^19^F NMR (282.4 MHz, CD_2_Cl_2_, δ): –74.49 (s, *J*_PtF_ = 11.3 Hz). ^13^C{^1^H} NMR (151 MHz, CD_2_Cl_2_, δ): 176.9 (C), 166.3 (C), 162.0 (*J*_PtC_ = 42.5 Hz, C), 160.7 (q, *J*_CF_ = 37.6 Hz, C), 150.8 (CH), 149.2 (CH), 145.7 (C), 143.4 (*J*_PtC_ = 23.1 Hz, C), 141.9 (*J*_PtC_ = 41.3 Hz, C), 141.0 (CH), 140.9 (CH), 139.9 (C), 138.7 (C), 133.7 (*J*_PtC_ = 31.9 Hz, CH), 130.0 (*J*_PtC_ = 89.9 Hz, C), 129.2 (CH), 127.8 (CH), 127.6 (CH), 125.1 (*J*_PtC_ = 11.7 Hz, CH), 125.1 (*J*_PtC_ = 13.3, CH), 123.7 (CH), 122.3 (*J*_PtC_ = 35.2 Hz, CH), 120.1 (CH), 120.0 (CH), 114.7 (q, *J*_CF_ = 295.8 Hz, CF_3_), 24.9 (CH_3_), 22.7 (CH_3_), 21.9 (CH_3_). Anal. Calcd for C_28_H_23_F_3_N_2_O_4_Pt: C, 47.80; H, 3.30; N, 3.98. Found: C, 47.77; H, 3.46; N, 3.94.

### Synthesis of (*OC*-6-34)-[Pt(tpy)_2_(O_2_CCF_3_)(O_2_CPh)] (**10**)

This complex was obtained as a white solid following the procedure described for **5**, but heating at 40 °C, from **4** (100 mg, 0.132 mmol), benzoic acid (101 mg, 0.827 mmol) and Na_2_CO_3_ (100 mg, 0.943 mmol). Yield: 71 mg, 70%. ^1^H NMR (401 MHz, CD_2_Cl_2_, δ): 9.38 (d, *J* = 5.1 Hz, 1H), 8.13–8.02 (m, 2H), 7.84–7.74 (m, 4H), 7.70 (s with satellites, *J*_PtH_ = 18.6 Hz, 1H), 7.60–7.49 (m, 4H), 7.37–7.31 (m, 1H), 7.30–7.23 (m, 3H), 6.95–6.86 (m, 2H), 6.13 (s with satellites, *J*_PtH_ = 36.9 Hz, 1H), 2.56 (s, 3H), 2.08 (s, 3H). ^19^F (282.4 MHz, CD_2_Cl_2_, δ): –75.15 (s, *J*_PtF_ = 10.9 Hz). ^13^C{^1^H} NMR (151 MHz, CD_2_Cl_2_, δ): 171.6 (C), 166.8 (C), 161.8 (s, *J*_PtC_ = 40.0 Hz, C), 160.9 (q, *J*_CF_ = 38.0 Hz), 150.6 (CH), 148.51 (CH), 145.8 (C), 143.4 (s, *J*_PtC_ = 25.0 Hz, C), 142.1 (C), 141.1 (CH), 140.9 (CH), 139.7 (C), 138.9 (C), 137.7 (C), 133.7 (s, *J*_PtC_ = 32.8 Hz, CH), 132.8 (CH), 132.5 (C), 130.8 (CH), 130.0 (CH), 129.6 (CH), 129.1 (CH), 128.1 (CH), 127.9 (CH), 127.8 (CH), 125.3 (s, *J*_PtC_ = 26.8 Hz, CH), 125.1 (s, *J*_PtC_ = 27.2 Hz, CH), 123.8 (CH), 122.6 (s, *J*_PtC_ = 35.4 Hz, CH), 120.3 (CH), 120.2 (CH), 114.8 (*J*_CF_ = 291.8 Hz), 22.7 (CH_3_), 22.0 (CH_3_). Anal. Calcd for C_33_H_25_F_3_N_2_O_4_Pt: C, 51.77; H, 3.29; N, 3.66. Found: C, 51.63; H, 3.41; N, 3.60.

### Synthesis of (*OC*-6-34)-[Pt(tpy)_2_(O_2_C-*t*-Bu)(O_2_CMe)] (**11**)

This complex was obtained as a white solid following the procedure described for **5**, from **1** (110 mg, 0.15 mmol), acetic acid (100 μL) and Na_2_CO_3_ (50 mg, 0.47 mmol). Yield: 98 mg, 91%. ^1^H NMR (600 MHz, CD_2_Cl_2_, δ): 9.24 (ddd, *J* = 5.5, 1.7, 0.8 Hz, 1H), 8.07–8.02 (m, 1H), 7.98 (d, *J* = 7.9 Hz, 1H), 7.74–7.67 (m, 3H), 7.56 (d, *J* = 7.9 Hz, 1H), 7.51 (d, *J* = 7.9 Hz, 1H), 7.51–7.45 (m, 2H), 7.24 (dd, *J* = 7.9, 1.7 Hz, 1H), 6.86–6.79 (m, 2H), 6.06 (s with satellites, *J*_PtH_ = 38.4 Hz, 1H), 2.55 (s, 3H), 2.02 (s, 3H), 1.79 (s, 3H), 0.97 (s, 9H). ^13^C NMR (151 MHz, CD_2_Cl_2_, δ): 182.2 (C), 176.9 (C), 166.0 (C), 162.2 (C), 150.4 (CH), 149.2 (*J*_PtC_ = 39.6 Hz), 146.0 (C), 142.7 (C), 141.2 (C), 140.4 (*J*_PtC_ = 20.6 Hz, CH), 140.3 (CH), 139.8 (C), 139.2 (C), 133.5 (CH), 131.5 (C), 129.9 (CH), 127.3 (CH), 126.7 (CH), 124.8 (*J*_PtC_ = 32.9 Hz), 124.7 (CH), 123.1 (CH), 122.1 (*J*_PtC_ = 27.8 Hz, CH), 119.7 (CH), 39.7 (C), 28.6 (3CH_3_), 25.2 (CH_3_) 22.6 (CH_3_), 21.9 (CH_3_). Anal. Calcd for C_31_H_32_N_2_O_4_Pt·0.5CH_2_Cl_2_: C, 51.53; H, 4.53; N, 3.82. Found: C, 51.62; H, 4.54; N, 3.47.

### Synthesis of (*OC*-6-33)-[Pt(tpy)_2_(O_2_CCF_3_)_2_] (**4′**)

A solution of complex **4** (40 mg, 0.05 mmol) in MeCN (5 mL) was irradiated for 1 h. The solvent was evaporated under reduced pressure, the residue was dissolved in CHCl_3_ (1 mL) and Et_2_O (10 mL) was added, whereupon a white solid precipitated, which was collected by filtration, washed with Et_2_O (2 × 3 mL) and vacuum-dried to give **4′**. Yield: 26 mg, 65%. ^1^H NMR (600 MHz, CD_2_Cl_2_, δ): 9.38 (d with satellites, *J*_PtH_ = 25.8 Hz, *J*_HH_ = 5.9 Hz, 2H), 8.16 (td, *J* = 7.9, 1.6 Hz, 2H), 7.98–7.94 (m, 2H), 7.59–7.54 (m, 2H), 7.52 (d, *J* = 7.9 Hz, 2H), 7.02 (d, *J* = 8.16 Hz, 2H), 5.75 (s with satellites, *J*_PtH_ = 33.7 Hz, 2H), 2.12 (s, 6H). ^19^F NMR (282 MHz, CD_2_Cl_2_, δ): –75.7 (s, 6 F). ^13^C NMR (151 MHz, CD_2_Cl_2_, δ): 164.4 (*J*_PtC_ = 59.7 Hz, C), 161.2 (q, *J*_CF_ = 36.7 Hz, C), 148.9 (CH), 143.1 (*J*_PtC_ = 42.6 Hz, C), 142.3 (CH), 139.3 (*J*_PtC_ = 27.2 Hz, C), 132.2 (*J*_PtC_ = 830.1 Hz, C), 129.0 (*J*_PtC_ = 28.5 Hz, CH), 128.2 (CH), 125.6 (*J*_PtC_ = 33.3 Hz, CH), 123.8 (*J*_PtC_ = 25.7 Hz, CH), 120.7 (*J*_PtC_ = 34.7 Hz, CH), 116.9 (q, *J*_CF_ = 290.3 Hz, CF_3_), 22.2 (CH_3_). Anal. Calcd for C_28_H_20_F_6_N_2_O_4_Pt: C, 44.39; H, 2.66; N, 3.70. Found: C, 44.49; H, 2.65; N, 3.73.

### Synthesis of 5-methyl-2-(2-pyridyl)phenyl pivalate (**12**)

A solution of complex **1** (55 mg, 0.075 mmol) in MeCN (5 mL) was irradiated for 2 h with a 365 nm LED source. The resultant crude mixture was adsorbed on silica gel that had been previously deactivated with a 9:1 (v/v) mixture of CH_2_Cl_2_ and NEt_3_ and then chromatographed on silica gel using a 9:1 (v/v) mixture of hexane and AcOEt; Et_3_N was previously added to the hexane fraction (1%). The collected fraction was evaporated under reduced pressure to give **12** as a yellow oil. Yield: 8 mg, 40%. ^1^H NMR (401 MHz, CD_2_Cl_2_, δ): 8.64 (ddd, *J* = 4.8, 1.8, 1.0 Hz, 1H), 7.70 (dt, *J* = 7.8, 1.9 Hz, 1H), 7.58 (d, *J* = 7.9 Hz, 1H), 7.48 (d, *J* = 7.9 Hz, 1H), 7.25–7.19 (m, 1H), 7.17 (d, *J* = 7.9 Hz, 1H), 6.90 (s, 1H), 2.41 (s, 3H), 1.21 (s, 9H). ^13^C{^1^H} NMR (101 MHz, CD_2_Cl_2_, δ): 177.3 (C), 156.1 (C), 150.0 (CH), 148.9 (C), 140.6 (C), 136.4 (CH), 131.4 (C), 131.0 (CH), 127.4 (CH), 124.4 (CH), 124.0 (CH), 122.5 (CH), 39.4 (C), 27.4 (3CH_3_), 21.4 (CH_3_). HRMS (ESI+, *m*/*z*): [M + H^+^]^+^ calcd for C_17_H_19_NO_2_, 270.1494; found, 270.1484. The NMR data are consistent with those found in the literature.^53^

### Synthesis of 5-methyl-2-(2-pyridyl)phenyl acetate (**13**)

This compound was obtained as a yellow oil following the procedure described for **12**, from **2** (40 mg, 0.062 mmol). Yield: 7 mg, 50%. The NMR data are consistent with those found in the literature.^37^

### Synthesis of 5-methyl-2-(2-pyridyl)phenyl benzoate (**14**)

This compound was obtained as a yellow oil following the procedure described for **12**, from **3** (50 mg, 0.065 mmol). Yield: 8 mg, 43%. ^1^H NMR (401 MHz, CD_2_Cl_2_, δ): 8.61 (ddd, *J* = 4.8, 1.9, 1.0 Hz, 1H), 8.16–8.13 (m, 2H), 7.78 (d, *J* = 7.9 Hz, 1H), 7.72–7.64 (m, 3H), 7.58–7.54 (m, 2H), 7.32 (d, *J* = 7.8 Hz, 1H), 7.24–7.20 (m, 1H), 7.18 (s, 1H), 2.53 (s, 3H). ^13^C{^1^H} NMR (101 MHz, CD_2_Cl_2_, δ): 165.7 (C), 156.0 (C), 150.0 (CH), 148.8 (C), 140.9 (C), 136.6 (CH), 133.9 (CH), 131.2 (CH), 131.0 (C), 130.5 (CH), 130.2 (C),129.0 (CH), 127.7 (CH), 124.3 (CH), 123.9 (CH), 122.5 (CH), 21.5 (CH_3_). HRMS (ESI+, *m*/*z*): [M + H^+^]^+^ calcd for C_19_H_15_NO_2_, 290.1181; found, 290.1179. The NMR data are consistent with those found in the literature.^54^

### Synthesis of *cis*-[Pt(tpy)(tpyO_2_CMe)] (**15**)

To a solution of complex **2** (65 mg, 0.100 mmol) in MeCN (5 mL) was added compound **13** (45 mg, 0.200 mmol) and the mixture was irradiated with a 365 nm LED source for 16 h. The solvent was removed under reduced pressure and the residue chromatographed on silica gel using a hexane/AcOEt mixture and gradually increasing the AcOEt proportion from 20–70%. The collected fraction was evaporated under reduced pressure and the residue was crystallized from CH_2_Cl_2_/Et_2_O (1:10) to give **15** (30 mg, 51%). ^1^H NMR (600 MHz, CD_2_Cl_2_, δ): 8.81 (d, *J* = 5.6 Hz, 1H), 8.72 (d, *J* = 5.6 Hz, 1H), 8.13 (dd, *J* = 8.3, 1.4 Hz, 1H), 7.96–7.75 (m, 5H), 7.54 (d, *J* = 7.8 Hz, 1H), 7.34–7.26 (m, 2H), 6.97 (d, *J* = 7.9 Hz, 1H), 6.64 (s, 1H), 2.41 (s, 9H). ^13^C{^1^H} NMR (151 MHz, CD_2_Cl_2_, δ): 169.8 (C), 166.9 (C), 164.6(C), 151.7 (C), 149.4 (C), 148.6 (CH), 148.2 (CH), 148.1 (C), 144.3 (C), 141.1 (C), 140.4 (C), 138.7 (*J*_PtC_ = 103.2 Hz, CH), 138.5 (CH), 138.4 (CH), 136.7 (*J*_PtC_ = 96.0 Hz, CH), 135.7 (C), 124.6 (CH), 123.8 (*J*_PtC_ = 42.4 Hz, CH), 123.2 (CH), 122.7 (CH), 122.4 (CH), 119.6 (CH), 119.1 (CH), 22.1 (CH_3_), 21.9 (CH_3_), 21.7 (CH_3_). Anal. Calcd for C_26_H_22_N_2_O_2_Pt: C, 52.97; H, 3.76; N, 4.75. Found: C, 53.01; H, 3.99; N, 4.41.

### Computational Methods

DFT calculations were carried out with Gaussian 16,^55^ using the B3LYP functional^56,57^ together with the 6-31G**^58,59^ basis set for the light atoms and the LANL2DZ^60^ basis set and effective core potential for the Pt atom. Optimizations were carried out without symmetry restrictions. Vertical excitation energies were obtained from TDDFT calculations at the ground-state optimized geometries. The solvent effect (MeCN) was accounted for in all cases by using the SMD variation of the Polarizable Continuum Model, as implemented in Gaussian.^61^ The optimized geometries were confirmed as energy minima by frequency calculations (zero imaginary frequencies). For a better interpretation of triplet vertical excitations, natural transition orbitals^62^ were computed for some of them using Gaussian; the dominant hole-particle pair is shown in each case (Figures S23–S25, S29–S31, S35–S37, S41–S43, S47–S49, S53–S55); when several hole-particle pairs with important eigenvalues were obtained, weighed sums of the hole cubes and, separately, the particle cubes, were performed using the ChemCraft software to obtain a hole-particle pair accounting for more than 90% of the transition.

## Supporting Information Available

Additional experimental details, X-ray structure determinations, crystallographic data, NMR spectra, and computational data (PDF)
