# Supplementary material for: Photoinduced Reductive C–O Couplings from Unsymmetrical Bis-Cyclometalated Pt(IV) Dicarboxylato Complexes
Source: Inorg Chem. 2024 Dec 27;64(1):662–73. doi: 10.1021/acs.inorgchem.4c03667 (PMC11734118; doi:10.1021/acs.inorgchem.4c03667)
Supplement: Supplementary file 1 — ic4c03667_si_001.pdf [file ic4c03667_si_001.pdf]

SUPPORTING INFORMATION

Photoinduced Reductive C–O Couplings from Unsymmetrical Bis-cyclometalated Pt(IV) Dicarboxylato Complexes

Juan Carlos López-López,[†] *Delia Bautista*[‡] *and Pablo González-Herrero*[*,†]

[†]Departamento de Química Inorgánica, Facultad de Química, Universidad de Murcia, Campus de Espinardo, 19, 30100 Murcia, Spain.

[‡]Área Científica y Técnica de Investigación, Universidad de Murcia, Campus de Espinardo, 21, 30100 Murcia, Spain.

*E-mail: pgh@um.es

Contents:

1. Additional experimental details 2
   1.1. Emission spectrum of the employed LEDs 2
   1.2. X-ray structure determinations 2
2. NMR spectra of new compounds 4
3. Computational data 22
   3.1. Complex 2 22
   3.2. Complex 4 27
   3.3. Complex 5 33
   3.4. Complex 6 39
   3.5. Complex 9 45
   3.6. Complex 11 51
4. References 60

1. Additional experimental details

1.1. Emission spectrum of the employed LEDs

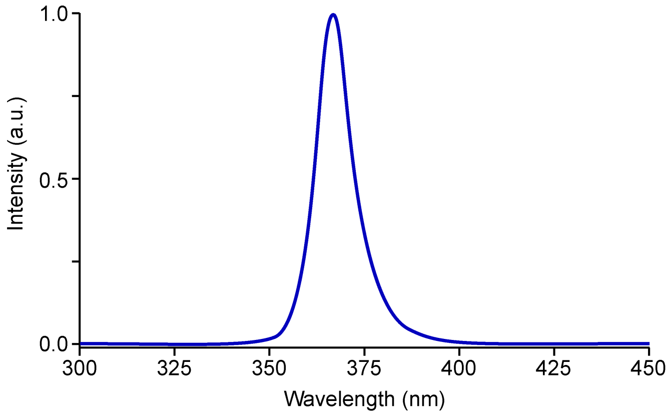

Figure S1. Emission spectrum of the LED emitters employed for irradiations.

1.2. X-ray structure determinations

Single crystals of **5** and **8** suitable for X-ray diffraction were obtained by slow liquid-liquid diffusion from CH_2Cl_2/hexane. The data were collected on a Bruker D8 QUEST diffractometer with monochromated Mo-$K\alpha$ radiation performing φ and ω scans. The structures were solved by direct methods and refined anisotropically on F^2 using the program SHELXL-2018 (G. M. Sheldrick, University of Göttingen).[1,2] Numerical details are given in Table S1. Methyl hydrogens were included as part of rigid idealized methyl groups allowed to rotate but not tip; other hydrogens were included using a riding model.

Table S1. Crystallographic data for **5** and **8**.

	**5**	**8**
formula	$C_{31}H_{32}N_2O_4Pt$	$C_{31}H_{29}F_3N_2O_4Pt$
fw	691.67	745.65
T (K)	100(2) K	100(2) K
λ (Å)	0.71073	0.71073
cryst syst	Monoclinic	Monoclinic
space group	$P2_1/n$	$P2_1/n$
a (Å)	10.1004(9)	10.2325(9)
b (Å)	19.4942(17)	19.5747(17)
c (Å)	13.8082(14)	13.9296(12)
α (°)	90	90
β (°)	94.908(3)	94.213(3)
γ (°)	90	90
V (Å^3)	2708.9(4)	2782.5(4)
Z	4	4
ρ_{calcd} (Mg m^{-3})	1.696	1.780
μ (mm^{-1})	5.219	5.103
R1[a]	0.0178	0.0183
wR2[b]	0.0421	0.0431

[a] $R1 = \Sigma||F_o|-|F_c||/\Sigma|F_o|$ for reflections with $I > 2\sigma(I)$. [b] $wR2 = [\Sigma[w(F_o^2-F_c^2)^2/\Sigma[w(F_o^2)^2]]^{0.5}$ for all reflections; $w^{-1} = \sigma^2(F^2) + (aP)^2 + bP$, where $P = (2F_c^2 + F_o^2)/3$ and a and b are constants set by the program.

2. NMR spectra of new compounds

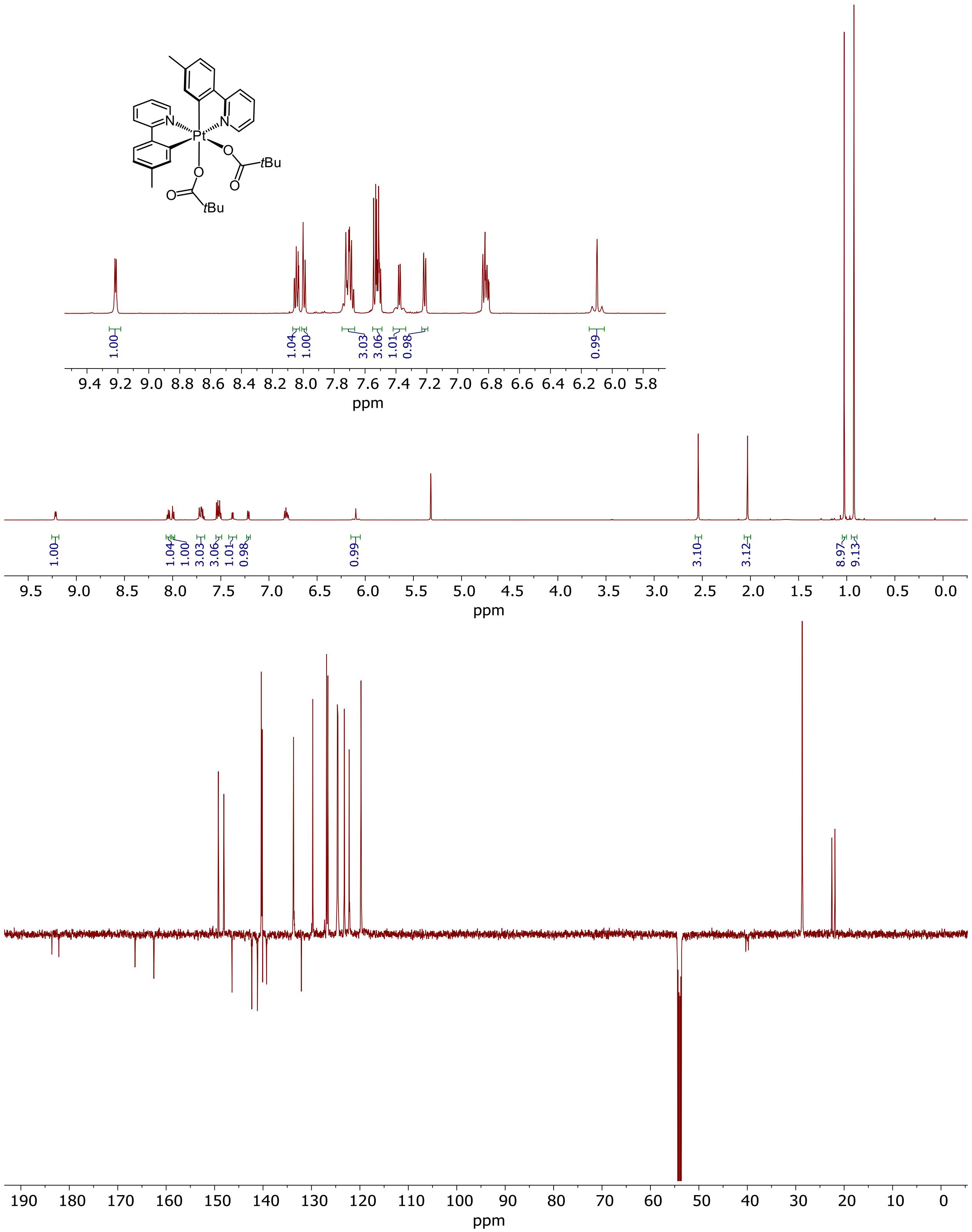

Figure S2. ^{1}H (top) and $^{13}C\{^{1}H\}$ APT (bottom) NMR spectra of complex **1** (CD_2Cl_2, 600 and 151 MHz, respectively).

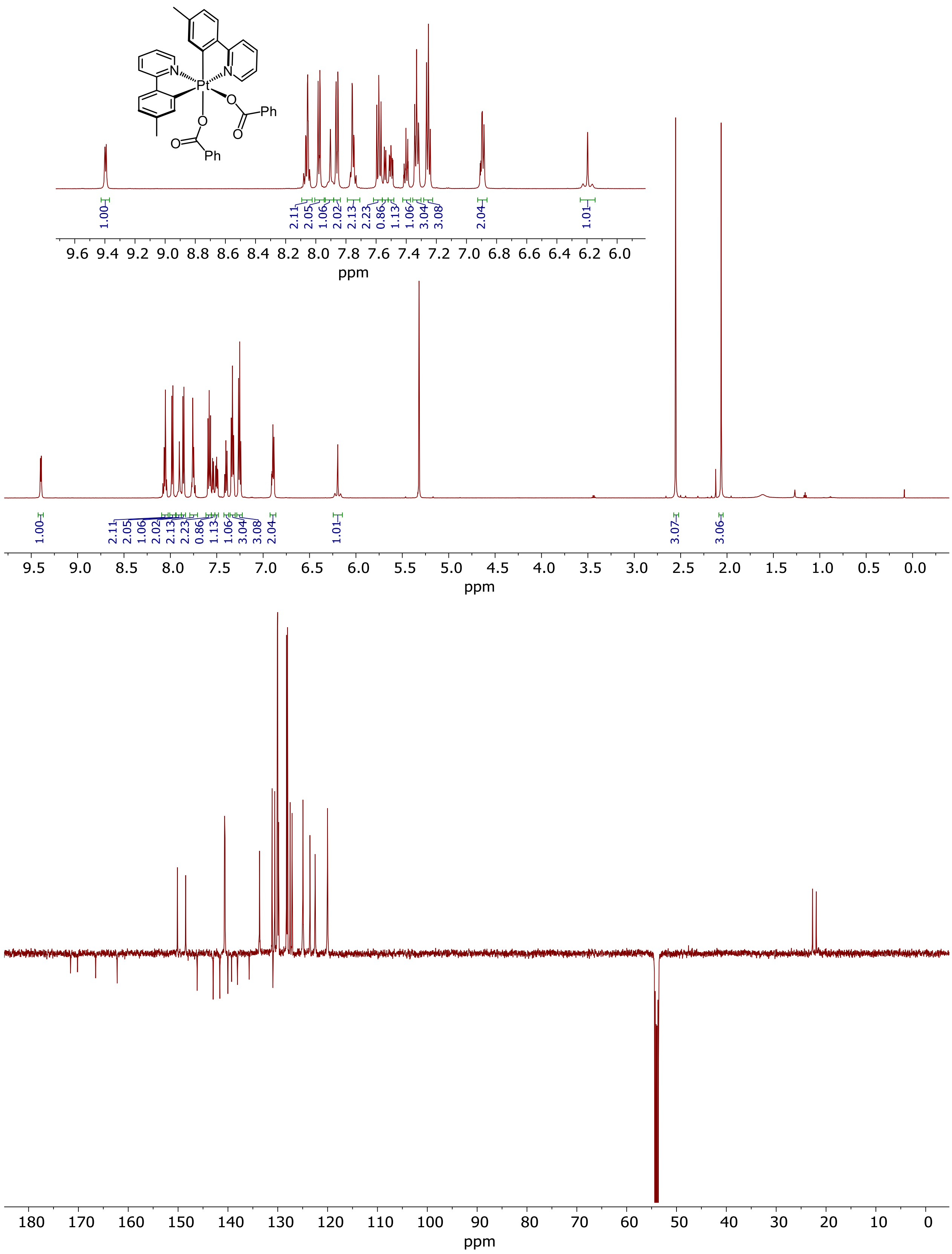

Figure S3. ^{1}H (top) and $^{13}C\{^{1}H\}$ APT (bottom) NMR spectra of complex **3** (CD_2Cl_2, 600 and 151 MHz, respectively).

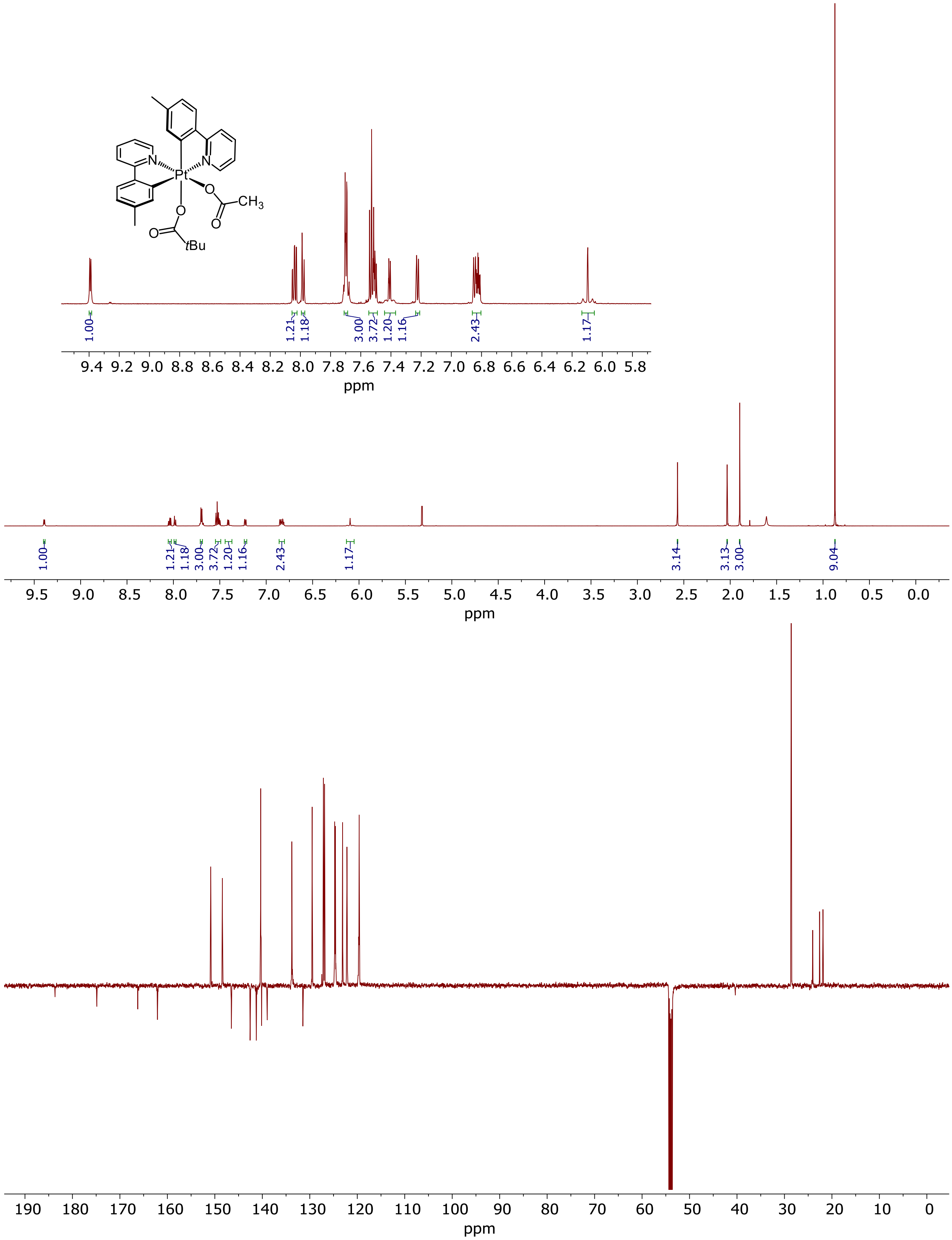

Figure S4. ^{1}H (top) and $^{13}C\{^{1}H\}$ APT (bottom) NMR spectra of complex **5** (CD_2Cl_2, 600 and 151 MHz, respectively).

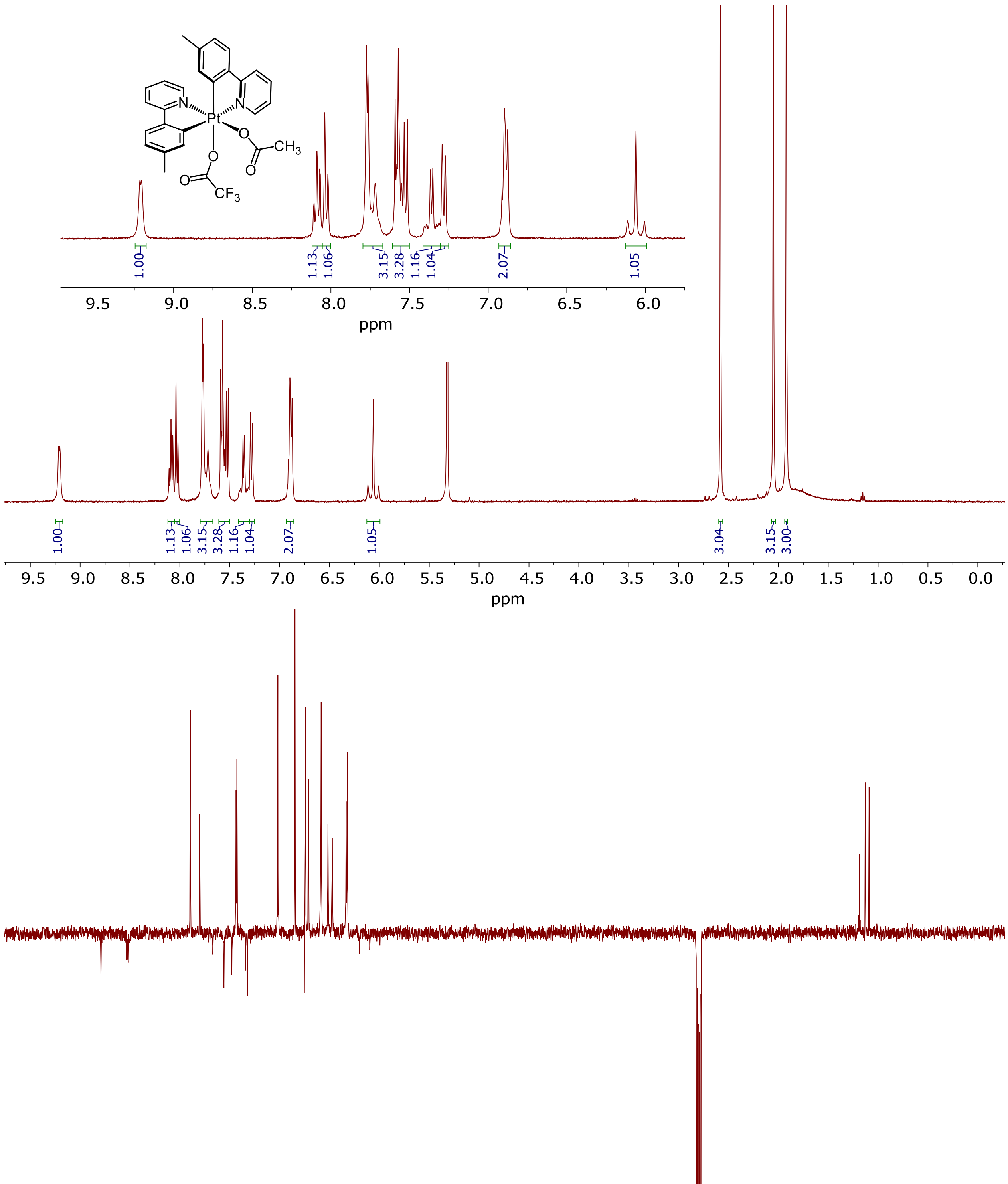

Figure S5. ^{1}H (top) and $^{13}C\{^{1}H\}$ APT (bottom) NMR spectra of complex **6** (CD_2Cl_2, 400 and 151 MHz, respectively).

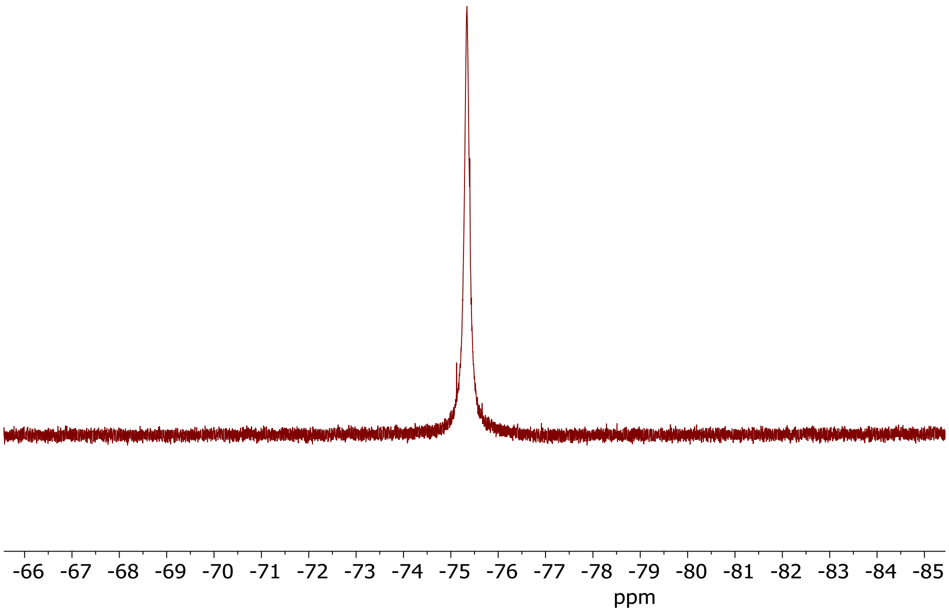

Figure S6. ^{19}F NMR spectrum of complex **6** (CD_2Cl_2, 377 MHz).

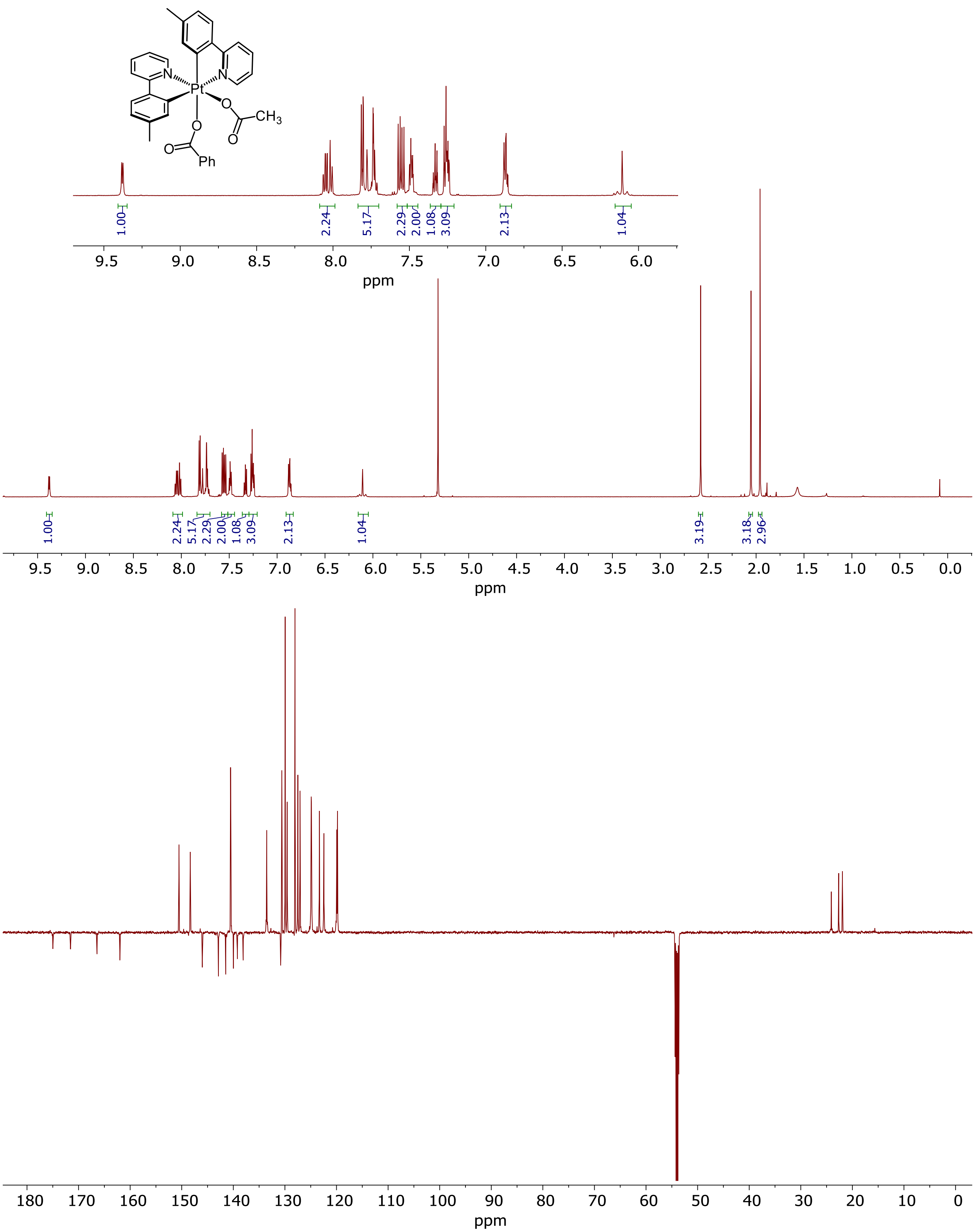

Figure S7. ^{1}H (top) and $^{13}C\{^{1}H\}$ APT (bottom) NMR spectra of complex **7** (CD_2Cl_2, 600 and 151 MHz, respectively).

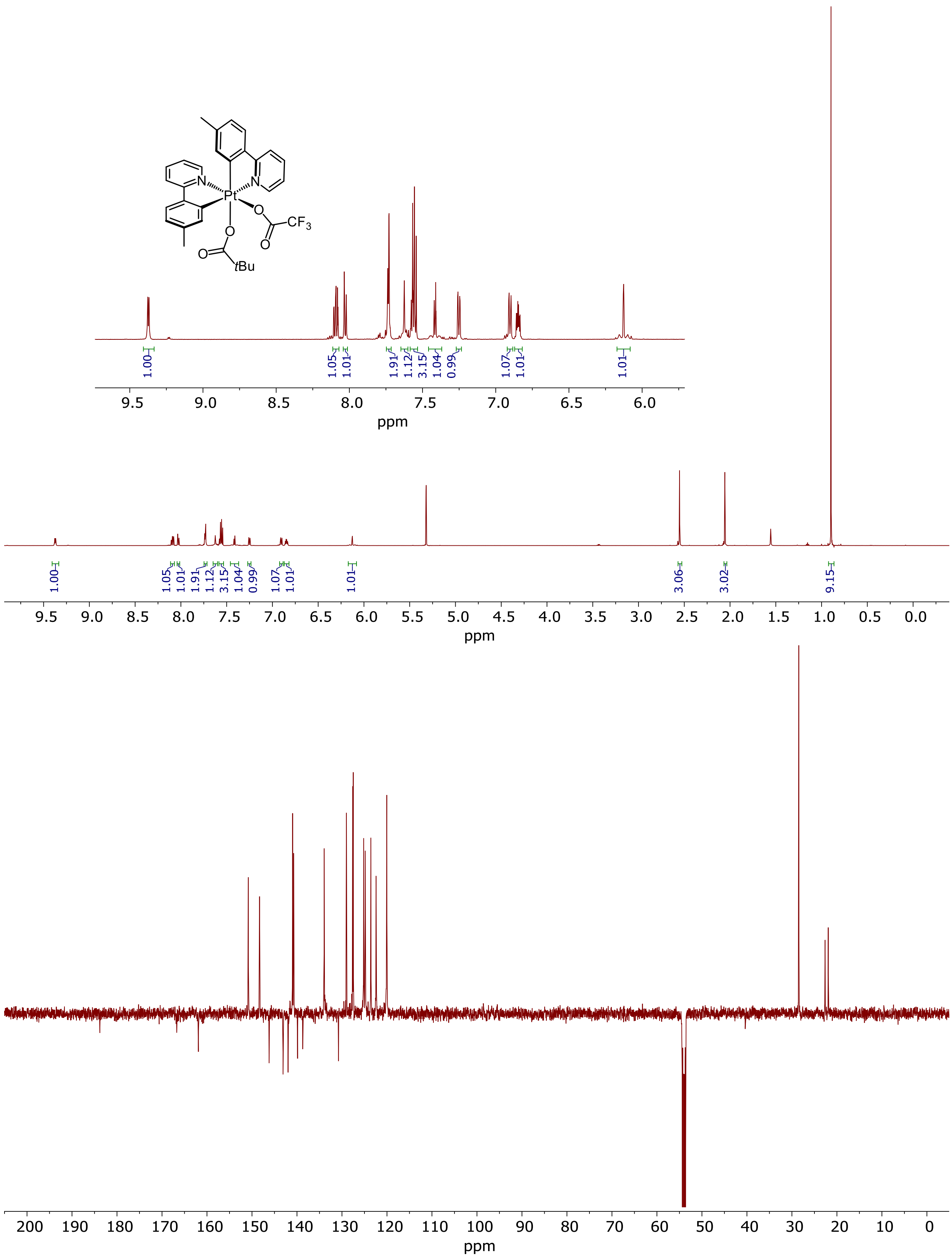

Figure S8. ^{1}H (top) and $^{13}C\{^{1}H\}$ APT (bottom) NMR spectra of complex **8** (CD_2Cl_2, 400 and 151 MHz, respectively).

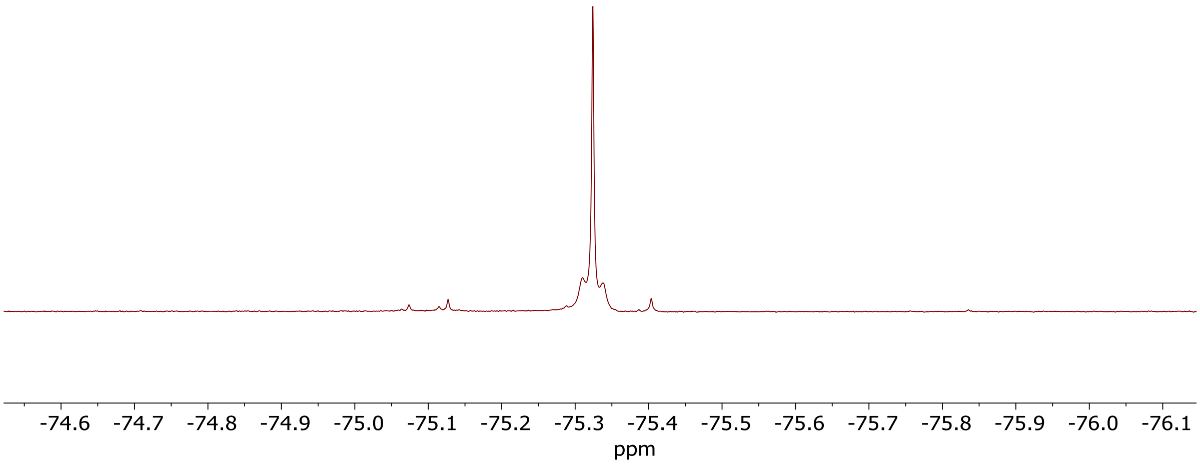

Figure S9. ^{19}F NMR spectrum of complex **8** (CD_2Cl_2, 377 MHz).

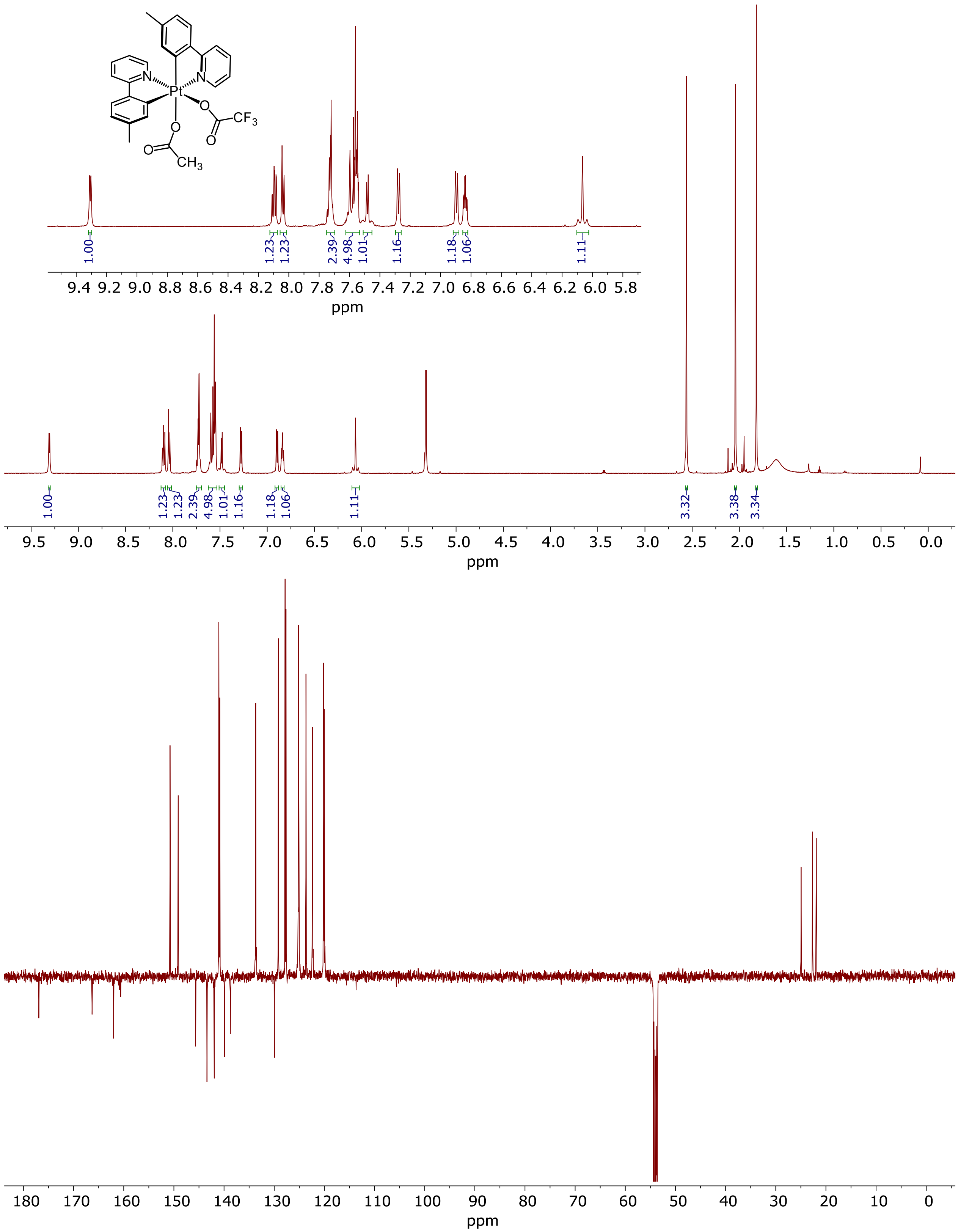

Figure S10. ^{1}H (top) and $^{13}C\{^{1}H\}$ APT (bottom) NMR spectra of complex **9** (CD_2Cl_2, 600 and 282 MHz, respectively).

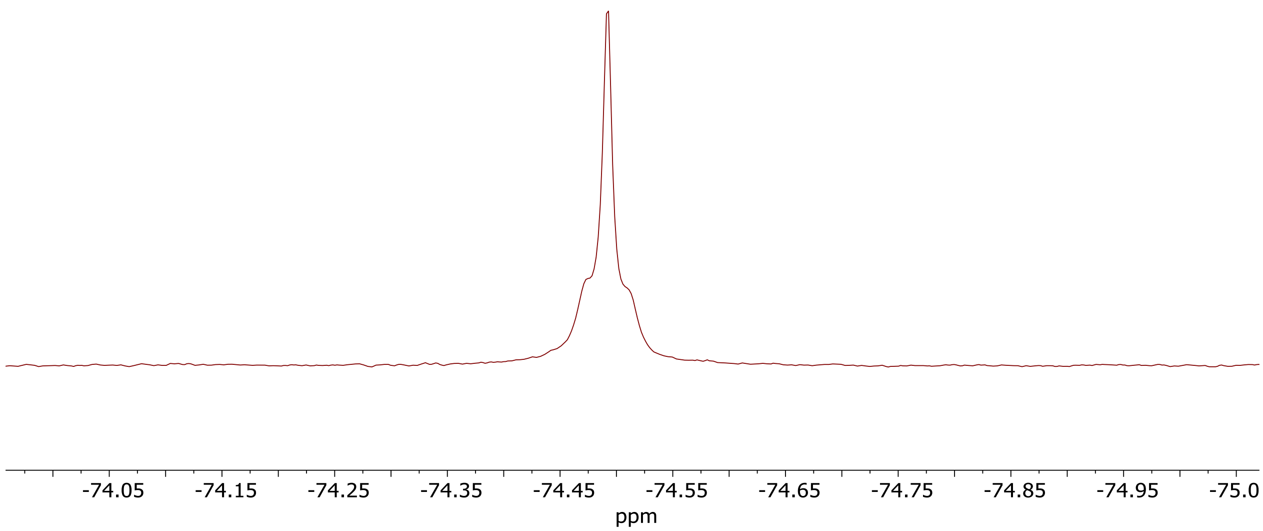

Figure S11. ^{19}F NMR spectrum of complex **9** (CD_2Cl_2, 282 MHz).

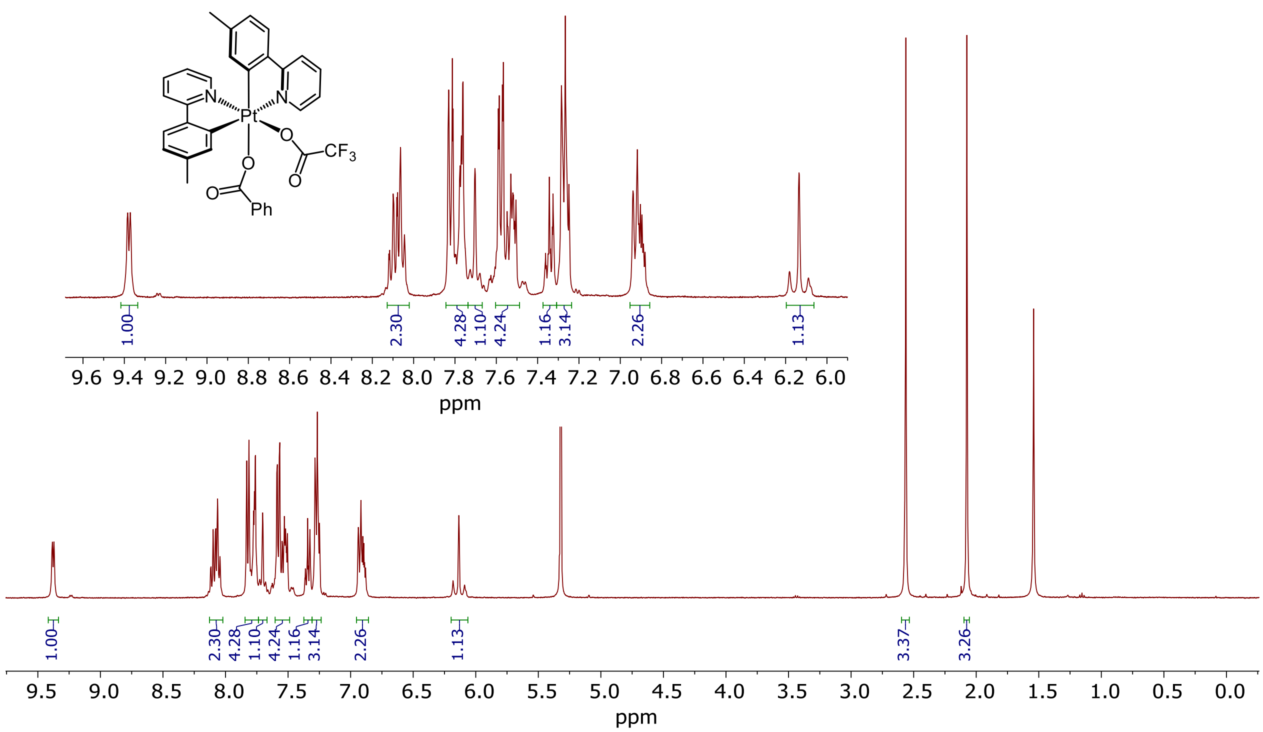

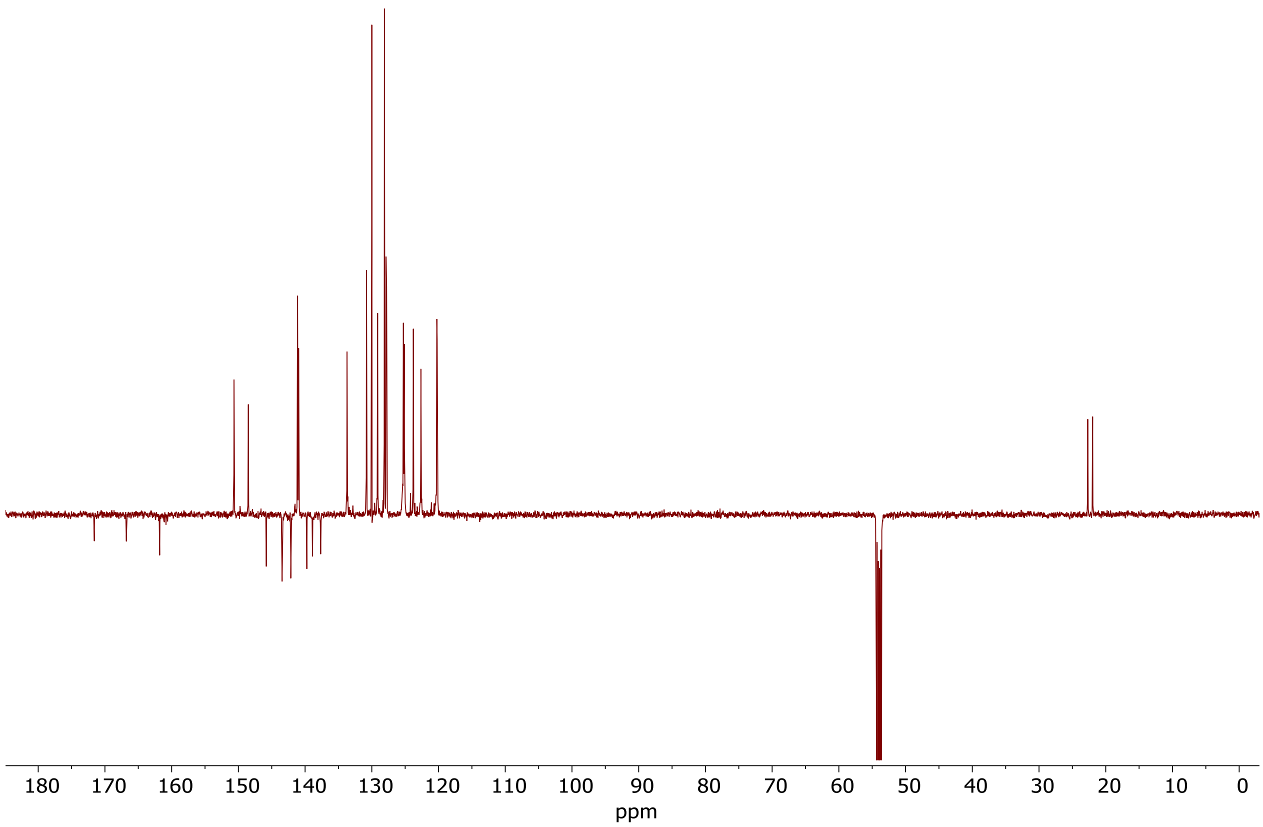

Figure S12. ^{1}H (top) and ^{13}C{^{1}H} APT (bottom) NMR spectra of complex **10** (CD_2Cl_2, 400 and 377 MHz, respectively).

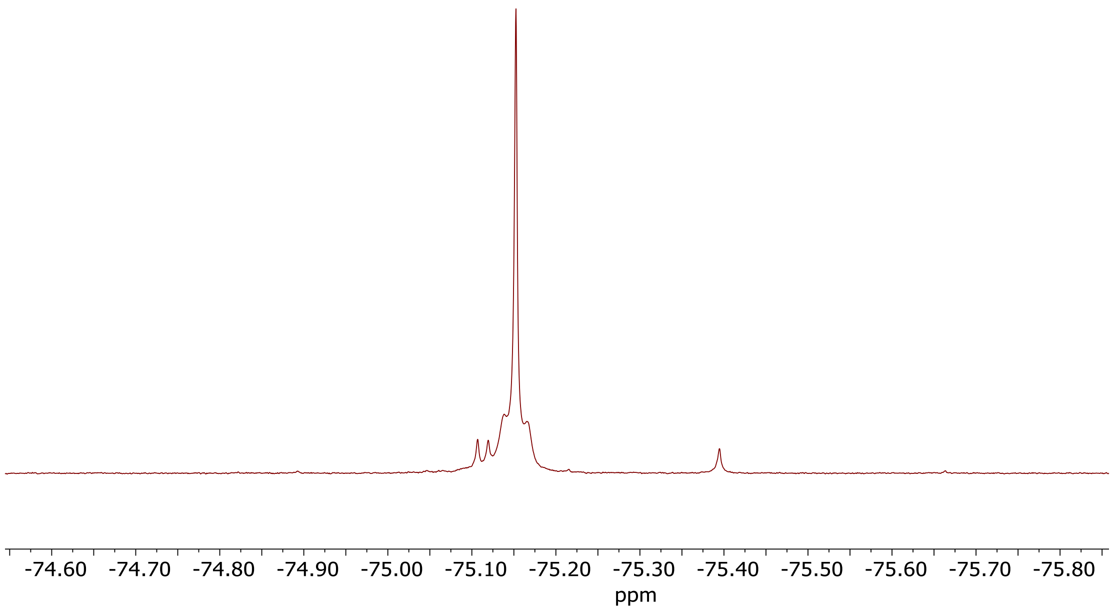

Figure S13. ^{19}F NMR spectrum of complex **10** (CD_2Cl_2, 377 MHz).

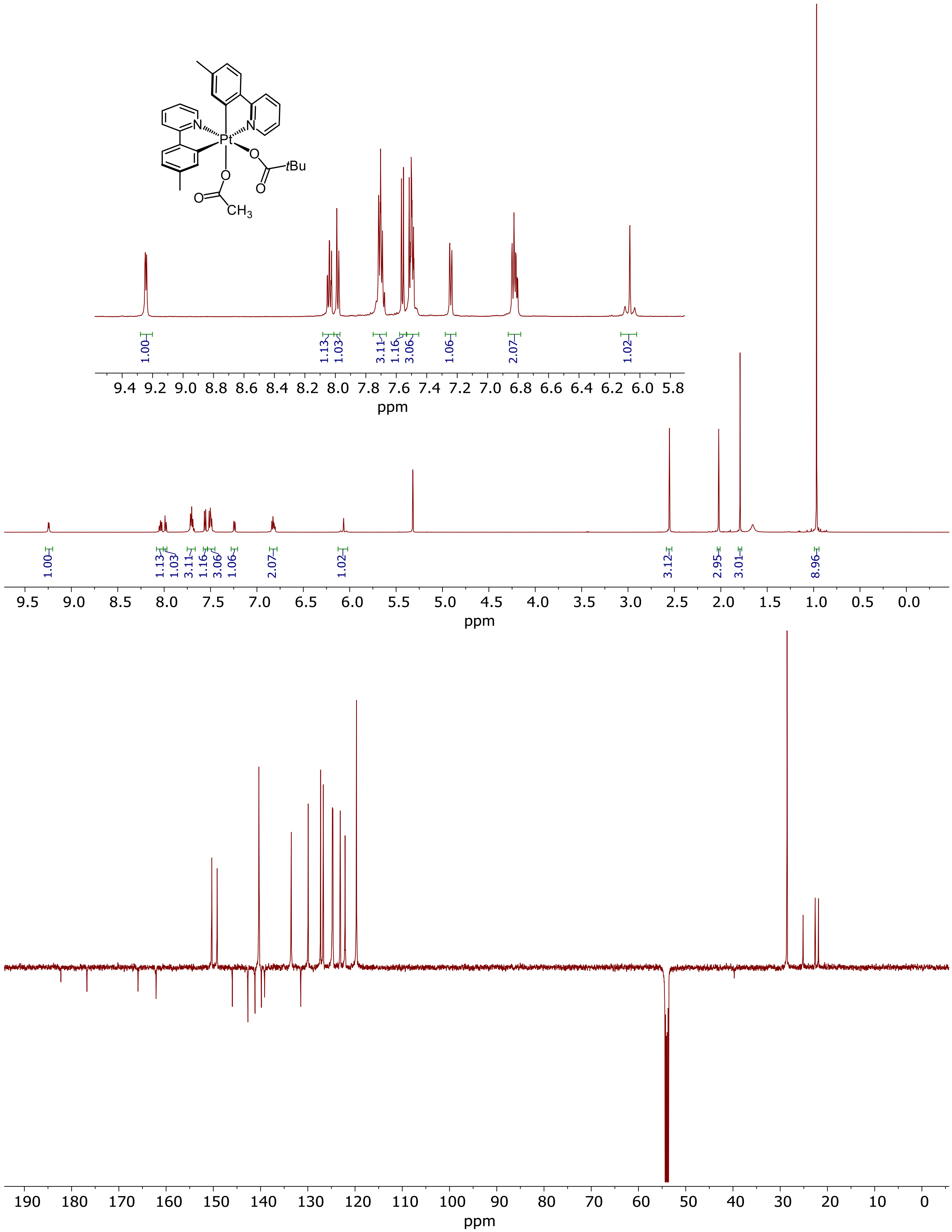

Figure S14. ^{1}H (top) and $^{13}C\{^{1}H\}$ APT (bottom) NMR spectra of complex **11** (CD_2Cl_2, 600 and 151 MHz, respectively).

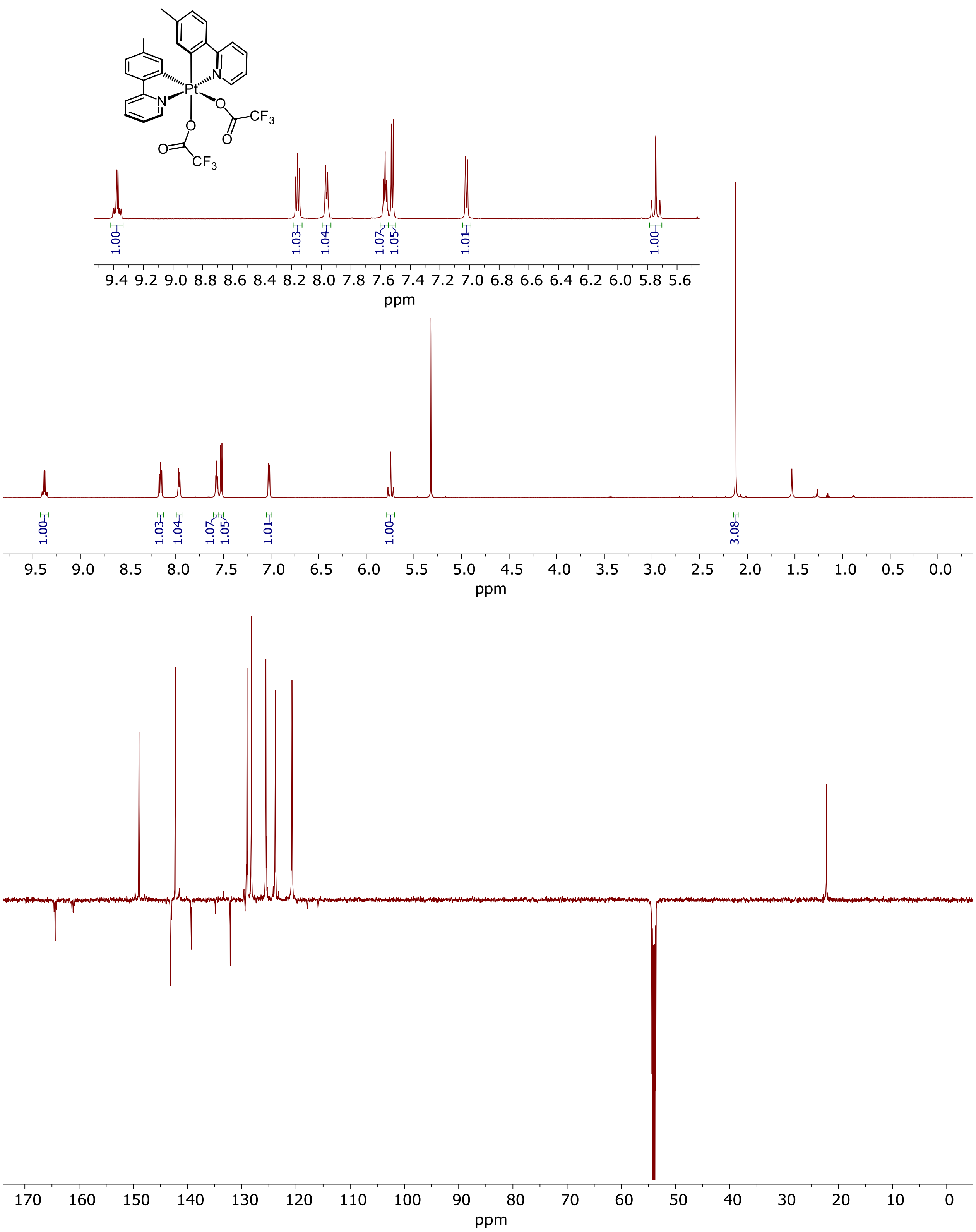

Figure S15. ^{1}H (top) and ^{13}C{^{1}H} APT (bottom) NMR spectra of complex **4'** (CD_2Cl_2, 600 and 282 MHz, respectively).

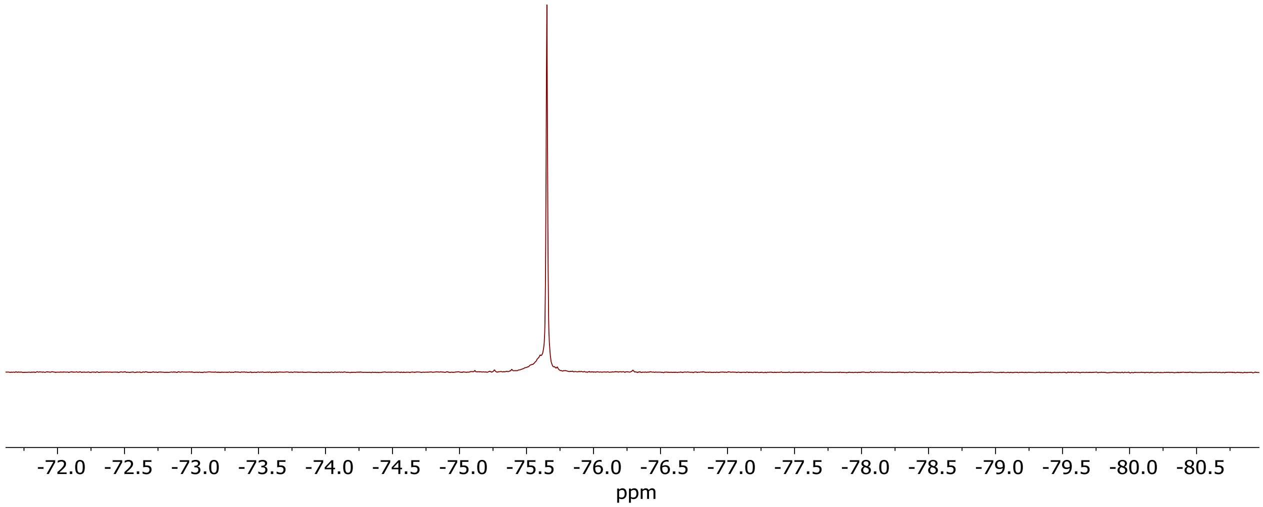

Figure S16. ^{9}F NMR spectrum of complex **4'** (CD_2Cl_2, 282 MHz).

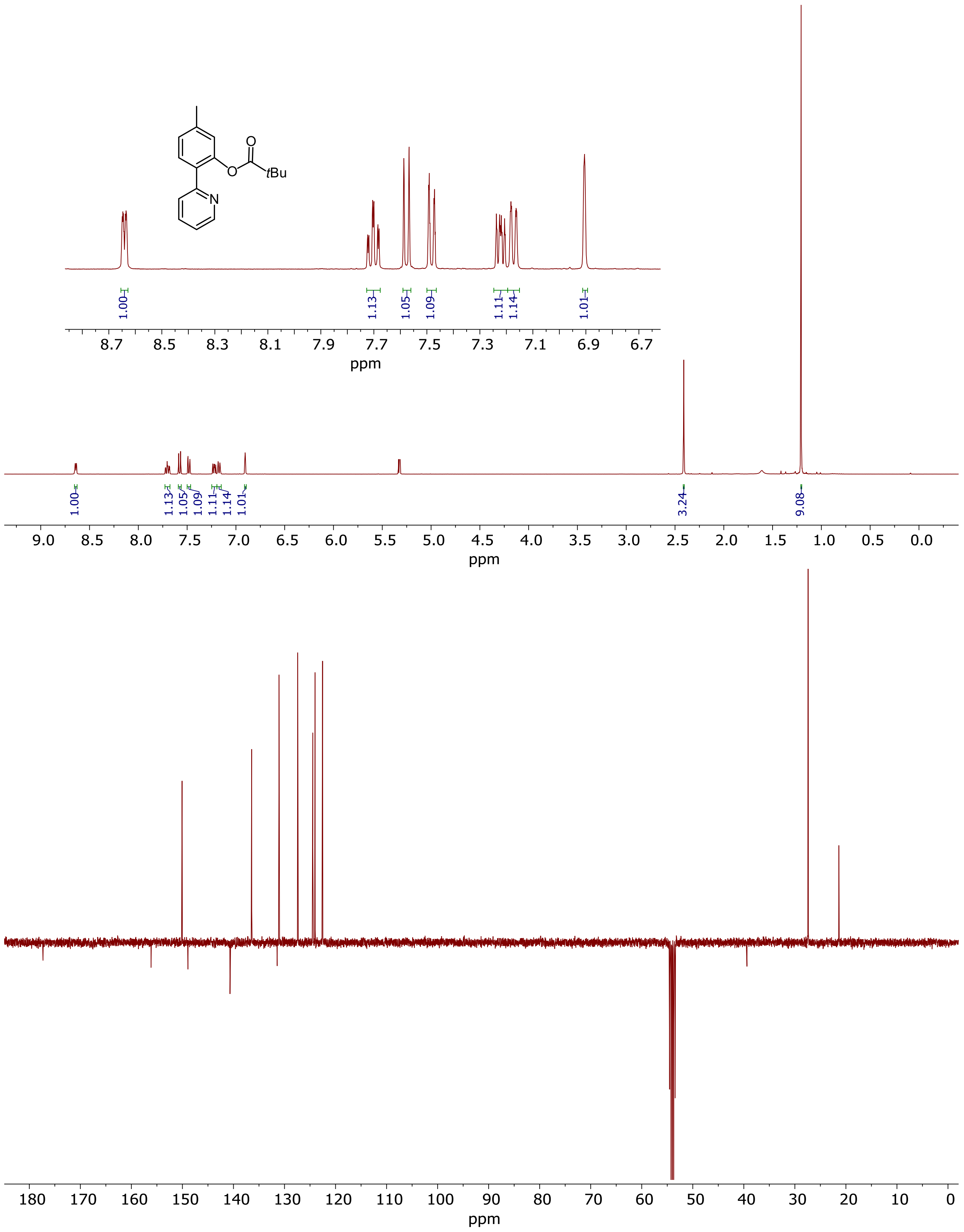

Figure S17. ^{1}H (top) and $^{13}C\{^{1}H\}$ APT (bottom) NMR spectra of compound **12** (CD_2Cl_2, 400 and 100 MHz, respectively).

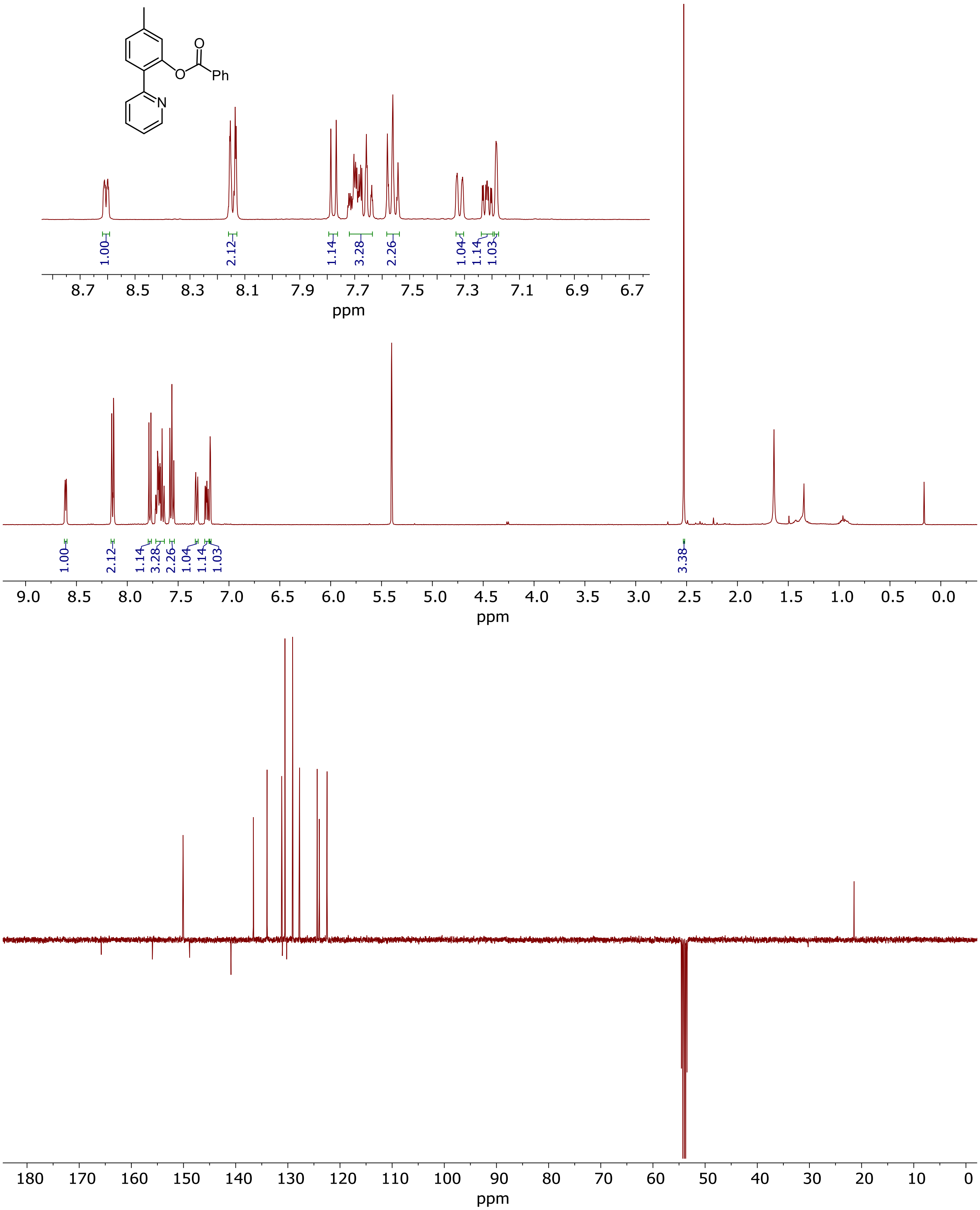

Figure S18. ^{1}H (top) and $^{13}C\{^{1}H\}$ APT (bottom) NMR spectra of compound **14** (CD_2Cl_2, 400 and 100 MHz, respectively).

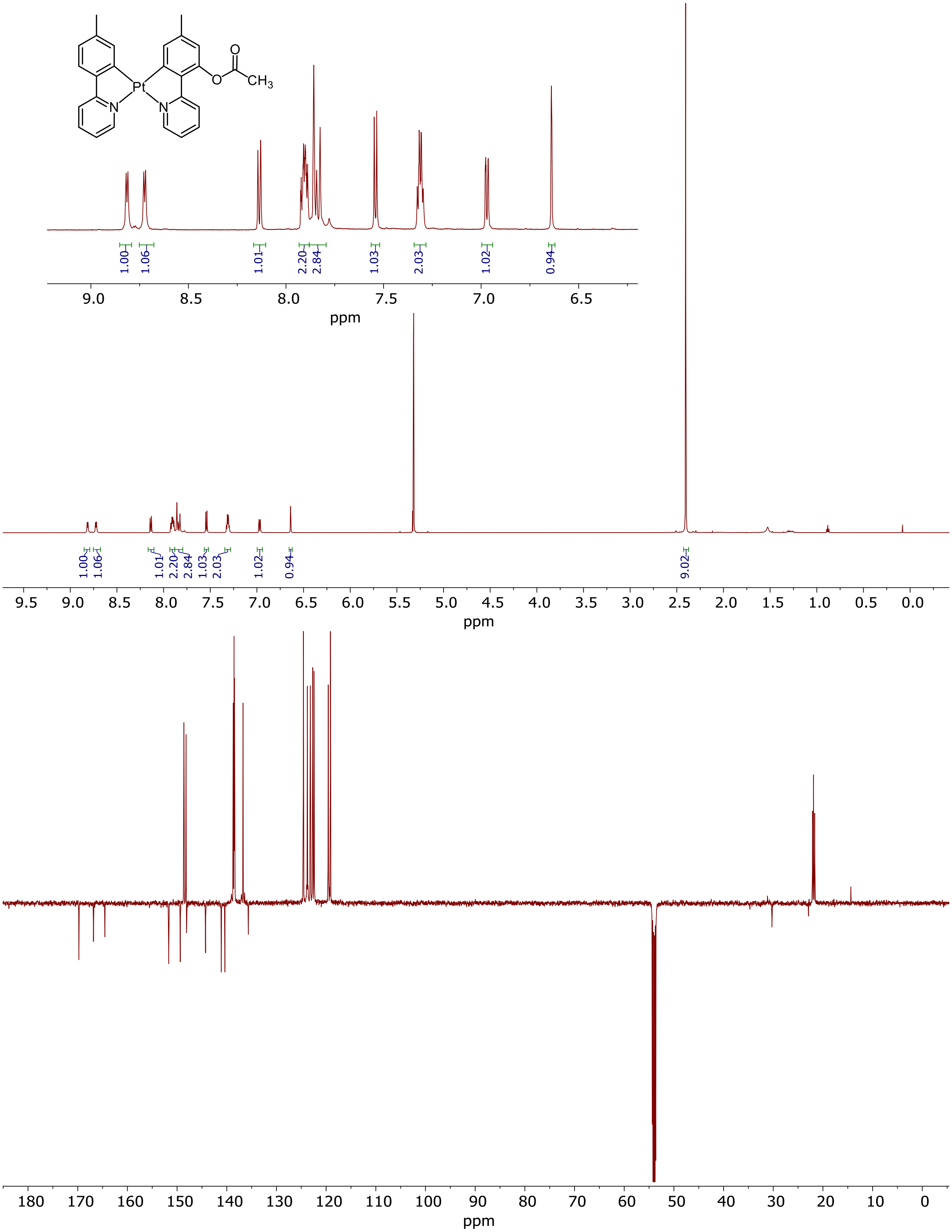

Figure S19. ^{1}H (top) and $^{13}C\{^{1}H\}$ APT (bottom) NMR spectra of complex **15** (CD_2Cl_2, 600 and 151 MHz, respectively).

3. Computational data

3.1. Complex 2

Table S2. Fragment contributions (%; from atomic orbital contributions) to the frontier orbitals of **2** in MeCN solution.

energy (a.u.)	number	L1	L2	L3	L4	Pt
–0.011	135 (LUMO+5)	30	27	0	6	36
–0.028	134 (LUMO+4)	14	84	0	0	2
–0.038	133 (LUMO+3)	84	15	0	0	2
–0.041	132 (LUMO+2)	25	11	15	2	46
–0.053	131 (LUMO+1)	1	97	0	0	1
–0.061	130 (LUMO)	95	0	0	1	3
–0.216	129 (HOMO)	19	2	0	75	3
–0.223	128 (HOMO–1)	5	90	0	2	3
–0.224	127 (HOMO–2)	78	5	0	14	3
–0.233	126 (HOMO–3)	2	3	82	11	3
–0.237	125 (HOMO–4)	41	18	10	24	8
–0.243	124 (HOMO–5)	22	44	4	23	7

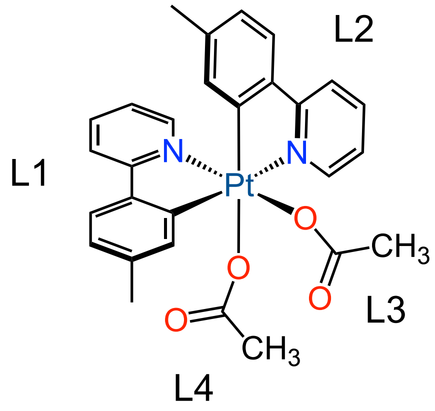

Figure S20. Ligand numbering in complex **2**.

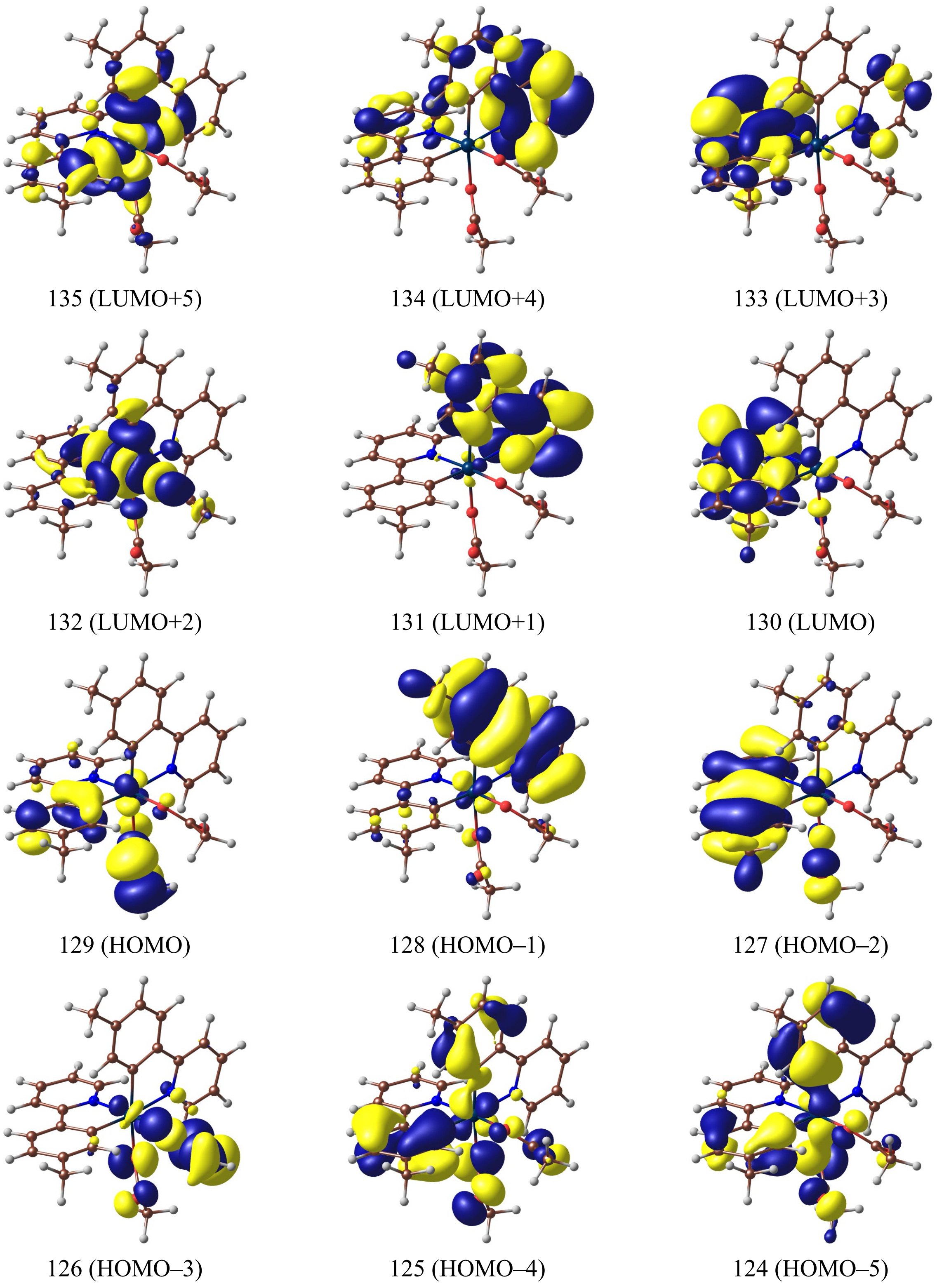

Figure S21. Molecular orbital isosurfaces of **2** (0.03 e $bohr^{-3}$).

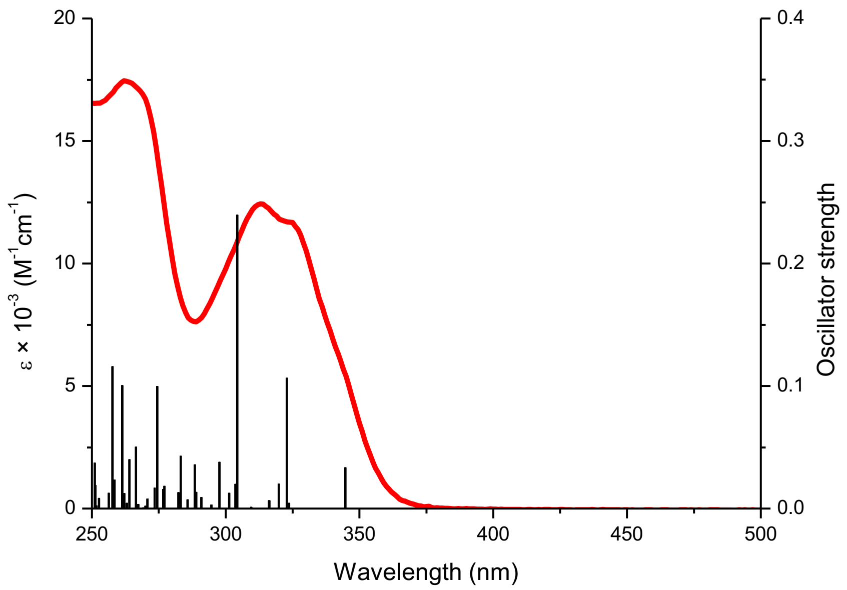

Figure S22. Calculated stick absorption spectrum of **2** compared with the experimental spectrum in MeCN solution (*ca.* 1×10^{-5} M) at 298 K.

Table S3. Selected vertical singlet excitations of **2** from TDDFT calculations at the ground state geometry in MeCN solution.

State	Monoexcitations	Coefficient (percentage)	ΔE/eV	λ/nm	Oscillator strength	Main character
S1	H → L	0.69143 (96%)	3.601	344.3	0.0345	LLCT (L4 → L1)
S2	H-1 → L<br>H-1 → L+2<br>H → L+2	0.60229 (73%)<br>0.15066 (5%)<br>-0.21953 (10%)	3.836	323.3	0.0054	LLCT (L2 → L1)
S3	H-2 → L<br>H-2 → L+2<br>H-1 → L<br>H → L<br>H → L+2	0.60241 (73%)<br>0.12718 (3%)<br>0.16746 (6%)<br>-0.10237 (2%)<br>0.21743 (9%)	3.845	322.5	0.1076	LC (L1)
S4	H-2 → L<br>H-2 → L+2<br>H-1 → L<br>H → L+2	-0.29406 (17%)<br>0.13108 (3%)<br>0.22838 (10%)<br>0.5576 (62%)	3.882	319.4	0.021	LMCT (L4 → Pt)
S5	H → L+1	0.69491 (97%)	3.925	315.9	0.0074	LLCT (L4 → L2)
S6	H-5 → L+2<br>H-4 → L<br>H-4 → L+2<br>H-3 → L<br>H-3 → L+2<br>H-1 → L<br>H-1 → L+2<br>H → L+2	-0.16833 (6%)<br>0.16114 (5%)<br>0.22989 (11%)<br>-0.20556 (8%)<br>-0.23011 (11%)<br>-0.2225 (10%)<br>0.42782 (37%)<br>0.13981 (4%)	4.011	309.1	0.0021	LMCT (L2→ Pt)

S7	H-1 → L+1	0.6581 (87%)	4.079	303.9	0.2405	LC (L2)
S8	H-4 → L<br>H-3 → L<br>H-2 → L+2<br>H-1 → L+2	-0.33314 (22%)<br>0.47377 (45%)<br>0.11168 (2%)<br>0.32694 (21%)	4.089	303.2	0.0209	LLCT (L3 → L1)
S9	H-3 → L<br>H-2 → L+1<br>H-2 → L+2<br>H → L+2	-0.15472 (5%)<br>-0.28081 (16%)<br>0.5663 (64%)<br>-0.18743 (7%)	4.121	300.9	0.0136	LMCT (L1 → Pt)
S10	H-4 → L<br>H-2 → L+1<br>H-2 → L+2	0.10325 (2%)<br>0.59601 (71%)<br>0.27824 (15%)	4.143	299.3	0.0002	LLCT (L1→ L2)

Table S4. Selected vertical triplet excitations of **2** from TDDFT calculations at the ground state geometry in MeCN solution.

State	Monoexcitations	Coefficient (Percentage)	ΔE/eV	λ/nm	Main Character
T1	H–2 → L<br>H–2 → L+3<br>H–1 → L<br>H → L	0.56291 (63%)<br>0.16523 (5%)<br>0.12143 (3%)<br>0.24329 (12%)	2.886	429.6	LC (L1)
T2	H–2 → L+1<br>H–1 → L+1<br>H–1 → L+4	–0.13149 (3%)<br>0.61204 (75%)<br>–0.16948 (6%)	2.957	419.3	LC (L2)
T3	H–5 → L<br>H–4 → L<br>H–2 → L<br>H–2 → L+3<br>H → L	0.12794 (3%)<br>0.25968 (13%)<br>–0.10003 (2%)<br>–0.13417 (4%)<br>0.56735 (64%)	3.462	358.2	LLCT (L4 → L1)
T4	H–13 → L+3<br>H–11 → L+3<br>H–5 → L+2<br>H–2 → L<br>H–2 → L+3<br>H–2 → L+4<br>H–1 → L<br>H–1 → L+3<br>H → L+2<br>H → L+3	–0.13804 (4%)<br>–0.13792 (4%)<br>0.10845 (2%)<br>–0.21322 (9%)<br>0.40826 (33%)<br>0.12415 (3%)<br>–0.14758 (4%)<br>0.1015 (2%)<br>0.19308 (7%)<br>0.21087 (9%)	3.627	341.9	LC(L1) / LMCT (L1 → Pt)
T5	H–8 → L+2<br>H–7 → L+2<br>H–5 → L+2<br>H–4 → L<br>H–2 → L<br>H–2 → L+2<br>H–2 → L+3<br>H–1 → L+2<br>H → L+2	–0.11045 (2%)<br>–0.10956 (2%)<br>0.2123 (9%)<br>–0.11718 (3%)<br>0.12228 (3%)<br>0.26215 (14%)<br>–0.17819 (6%)<br>–0.11672 (3%)<br>0.42008 (35%)	3.639	340.7	LMCT (L1/L4 → Pt)

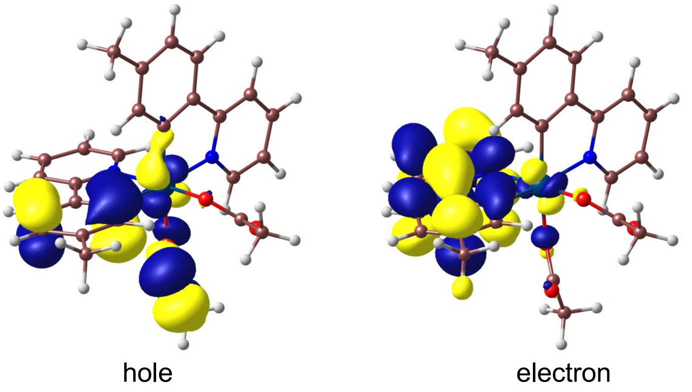

Figure S23. Natural transition orbitals of the T_3 excitation of complex **2** (0.03 e bohr^{-3}).

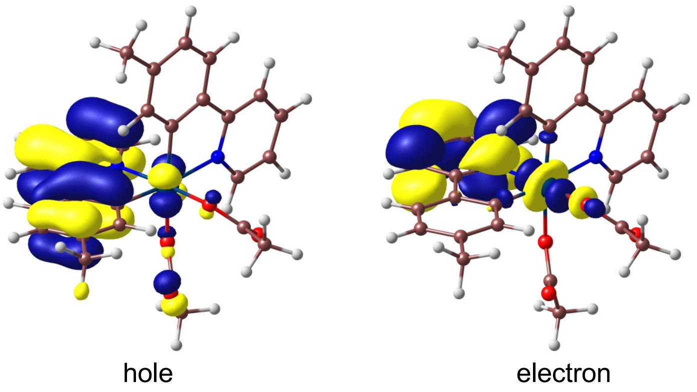

Figure S24. Natural transition orbitals of the T_4 excitation of complex **2** (0.03 e bohr^{-3}).

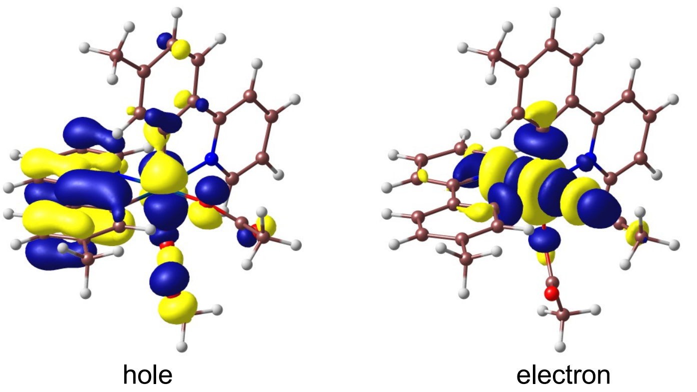

Figure S25. Natural transition orbitals of the T_5 excitation of complex **2** (0.03 e bohr^{-3}).

3.2. Complex 4

Table S5. Fragment contributions (%; from atomic orbital contributions) to the frontier orbitals of **4** in MeCN solution.

energy (a.u.)	number	L1	L2	L3	L4	Pt
–0.026	159 (LUMO+5)	30	27	0	6	36
–0.031	158 (LUMO+4)	14	84	0	0	2
–0.041	157 (LUMO+3)	84	15	0	0	1
–0.055	156 (LUMO+2)	25	10	15	2	47
–0.058	155 (LUMO+1)	1	97	0	0	1
–0.068	154 (LUMO)	95	0	0	1	3
–0.227	153 (HOMO)	5	92	0	0	2
–0.229	152 (HOMO–1)	91	7	0	0	2
–0.247	151 (HOMO–2)	68	20	0	5	7
–0.249	150 (HOMO–3)	21	68	0	3	7
–0.258	149 (HOMO–4)	6	4	2	86	2
–0.265	148 (HOMO–5)	3	7	60	27	4

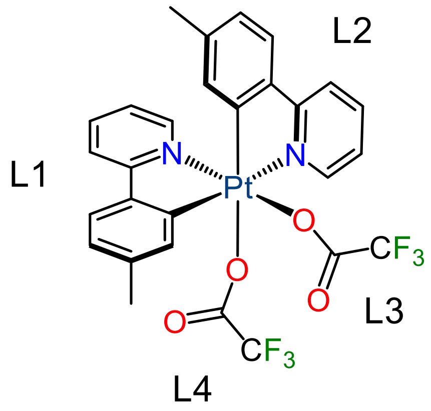

Figure S26. Ligand numbering in complex **4**.

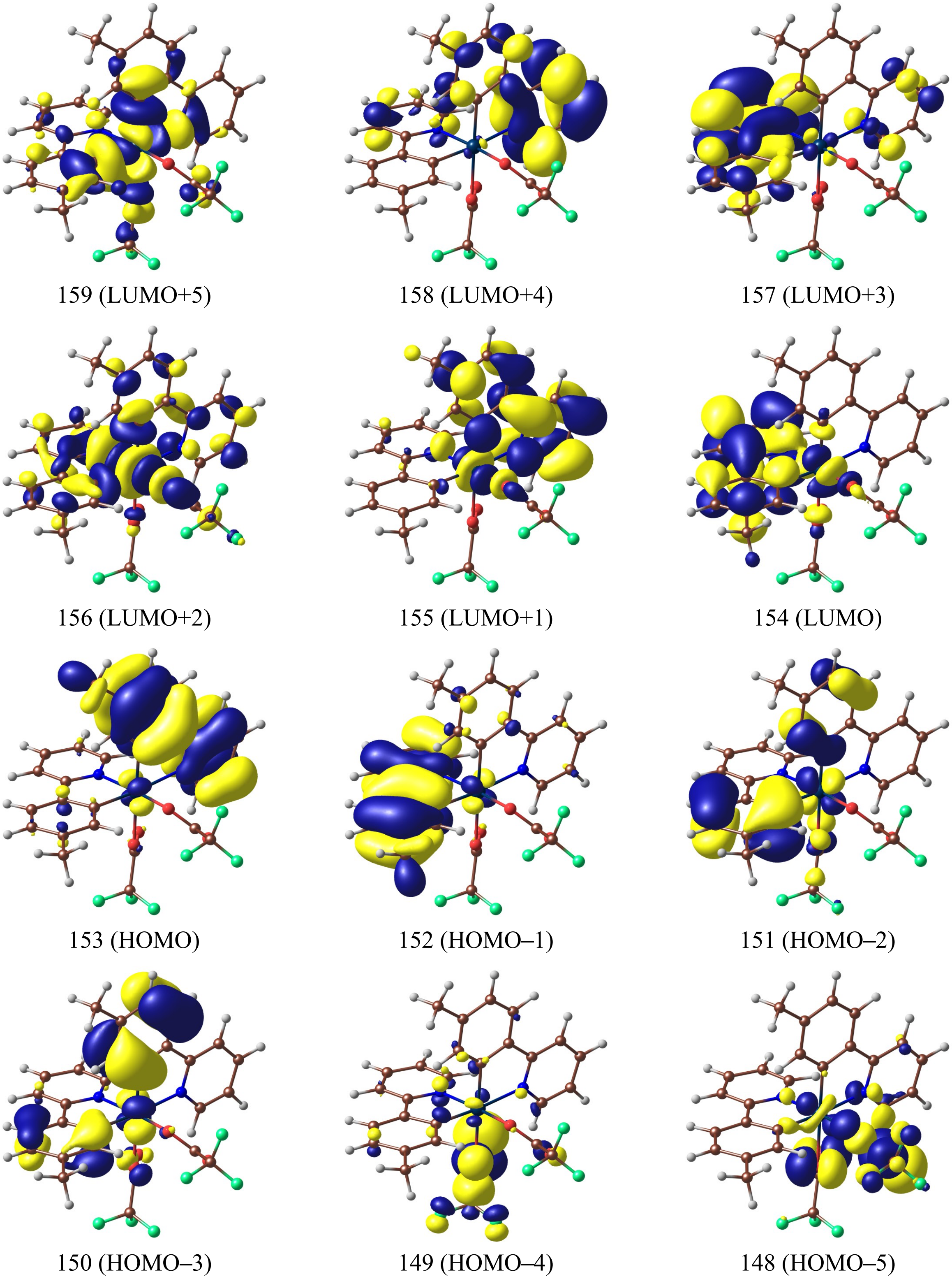

Figure S27. Molecular orbital isosurfaces of **4** (0.03 e bohr^{-3}).

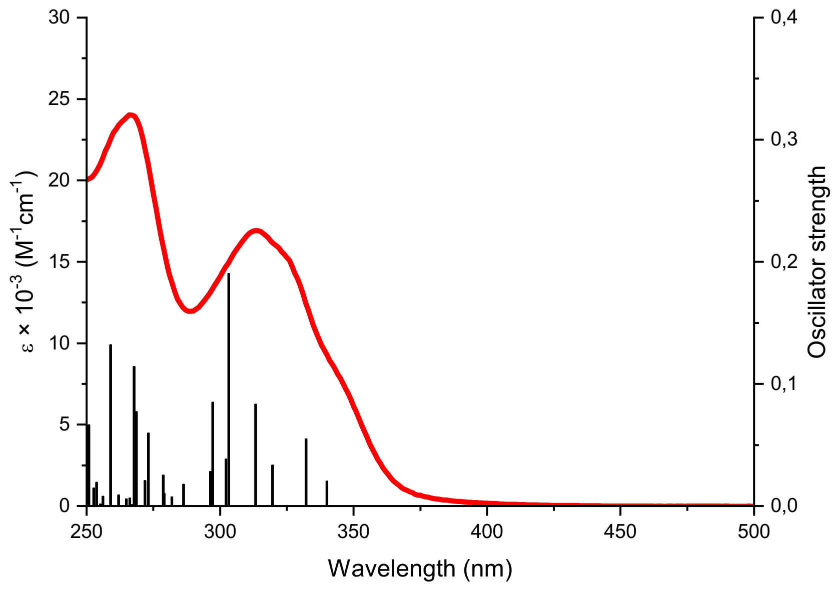

Figure S28. Calculated stick absorption spectrum of **4** compared with the experimental spectrum in MeCN solution (*ca.* 1×10^{-5} M) at 298 K.

Table S6. Selected vertical singlet excitations of **4** from TDDFT calculations at the ground state geometry in MeCN solution.

State	Monoexcitations	Coefficient (probability)	ΔE/eV	λ/nm	Oscillator strength	Main character
S1	H–3 → L<br>H–3 → L+2<br>H–1 → L<br>H–1 → L+2<br>H → L<br>H → L+1<br>H → L+2	–0.10716 (2%)<br>0.10579 (2%)<br>0.19946 (8%)<br>–0.13312 (4%)<br>0.53656 (58%)<br>–0.15701 (5%)<br>–0.28967 (17%)	3.647	340.0	0.0203	LLCT (L2 → L1)
S2	H–1 → L<br>H–1 → L+1<br>H–1 → L+2<br>H → L	0.57766 (67%)<br>–0.10987 (2%)<br>–0.2682 (14%)<br>–0.21636 (9%)	3.732	332.2	0.0551	LC (L1) / LMCT (L1 → Pt)
S3	H–3 → L+2<br>H–2 → L<br>H–1 → L<br>H–1 → L+1<br>H–1 → L+2<br>H → L<br>H → L+1<br>H → L+2	–0.13167 (3%)<br>0.11375 (3%)<br>0.13764 (4%)<br>0.12145 (3%)<br>0.22758 (10%)<br>0.36667 (27%)<br>0.25571 (13%)<br>0.39987 (32%)	3.879	319.6	0.0336	LLCT (L2 → L1) / LMCT (L2→ Pt)
S4	H–2 → L+2<br>H–1 → L	0.10001 (2%)<br>0.26607 (14%)	3.957	313.4	0.0833	LC (L1)

	H–1 → L+1<br>H–1 → L+2<br>H → L<br>H → L+1<br>H → L+2	0.28077 (16%)<br>0.46863 (44%)<br>–0.15315 (5%)<br>–0.15761 (5%)<br>–0.19707 (8%)				
S5	H–2 → L<br>H–2 → L+2<br>H → L+1<br>H → L+2	–0.17208 (6%)<br>0.1084 (2%)<br>0.57812 (67%)<br>–0.28901 (17%)	4.088	303.3	0.1904	LC (L2)
S6	H–3 → L<br>H–3 → L+1<br>H–3 → L+2<br>H–2 → L<br>H–2 → L+1<br>H–2 → L+2<br>H → L+2	0.26903 (14%)<br>–0.12158 (3%)<br>–0.1637 (5%)<br>0.42168 (36%)<br>–0.13563 (4%)<br>–0.23461 (11%)<br>–0.31122 (19%)	4.103	302.2	0.0385	LC (L1)
S7	H–3 → L<br>H–3 → L+2<br>H–2 → L<br>H–1 → L+1<br>H–1 → L+2<br>H → L+1	0.31268 (20%)<br>–0.1308 (3%)<br>–0.25905 (13%)<br>0.45665 (42%)<br>–0.2514 (13%)<br>–0.10388 (2%)	4.171	297.3	0.085	LLCT (L1 → L2)
S8	H–3 → L<br>H–3 → L+2<br>H–2 → L<br>H–1 → L<br>H–1 → L+1<br>H–1 → L+2<br>H–1 → L+3	–0.34267 (23%)<br>0.17716 (6%)<br>0.28443 (16%)<br>–0.10158 (2%)<br>0.41137 (34%)<br>–0.21569 (9%)<br>0.1068 (2%)	4.184	296.4	0.0283	LLCT (L2 → L1)
S9	H–3 → L<br>H–2 → L<br>H–2 → L+1<br>H–2 → L+2	0.14839 (4%)<br>0.27535 (15%)<br>0.27835 (15%)<br>0.51563 (53%)	4.331	286.3	0.0178	LMCT (L1 → Pt)
S10	H–3 → L<br>H–3 → L+1<br>H–3 → L+2<br>H–2 → L<br>H–1 → L+3	0.35438 (25%)<br>0.33185 (22%)<br>0.4177 (35%)<br>0.11862 (3%)<br>–0.16278 (5%)	4.398	281.9	0.0074	LC (L2)/LLCT (L2 → L1)/LMCT (L2 → Pt)

Table S7. Selected vertical triplet excitations of **4** from TDDFT calculations at the ground state geometry in MeCN solution.

State	Monoexcitations	Coefficient (Percentage)	ΔE/eV	λ/nm	Main Character
T1	H–2 → L+7<br>H–1 → L<br>H–1 → L+2<br>H–1 → L+3<br>H → L	0.10283 (2%)<br>0.59555 (71%)<br>0.14916 (4%)<br>0.1742 (6%)<br>0.15888 (5%)	2.863	433.1	LC(L1)
T2	H–3 → L+6<br>H–1 → L+1<br>H → L+1<br>H → L+2<br>H → L+4	0.10887 (2%)<br>–0.13371 (4%)<br>0.57165 (65%)<br>–0.23774 (11%)<br>–0.17551 (6%)	2.950	420.3	LC(L2)
T3	H–3 → L<br>H–3 → L+1<br>H–3 → L+2<br>H–1 → L+2<br>H → L<br>H → L+1<br>H → L+2	0.1786 (6%)<br>–0.10495 (2%)<br>–0.20347 (8%)<br>0.21806 (10%)<br>–0.3501 (25%)8<br>0.15039 (5%)<br>0.37853 (29%)	3.512	353.1	LMCT (L1/L2 → Pt)
T4	H–11 → L+3<br>H–2 → L<br>H–1 → L<br>H–1 → L+2<br>H–1 → L+3<br>H–1 → L+4<br>H → L	0.16163 (5%)<br>–0.25051 (13%)<br>–0.29123 (17%)<br>0.24939 (12%)<br>0.37473 (28%)<br>–0.109 (2%)<br>0.13108 (3%)	3.609	343.6	LC (L1) / LMCT (L1 → Pt)
T5	H–11 → L+3<br>H–3 → L<br>H–2 → L<br>H–2 → L+2<br>H–1 → L+1<br>H–1 → L+2<br>H–1 → L+3<br>H → L<br>H → L+3	0.13972 (4%)<br>–0.11248 (3%)<br>0.23207 (11%)<br>–0.12943 (3%)<br>–0.2225 (10%)<br>–0.31827 (20%)<br>0.35478 (25%)<br>–0.20809 (9%)<br>0.11346 (3%)	3.695	335.5	LMCT (L1 → Pt)

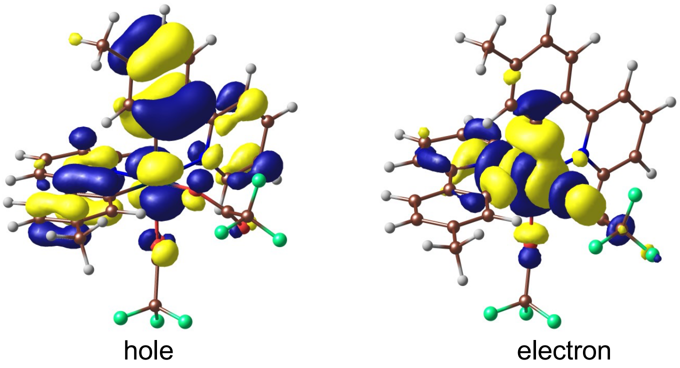

Figure S29. Natural transition orbitals of the T_3 excitation of complex **4** (0.03 e $bohr^{-3}$).

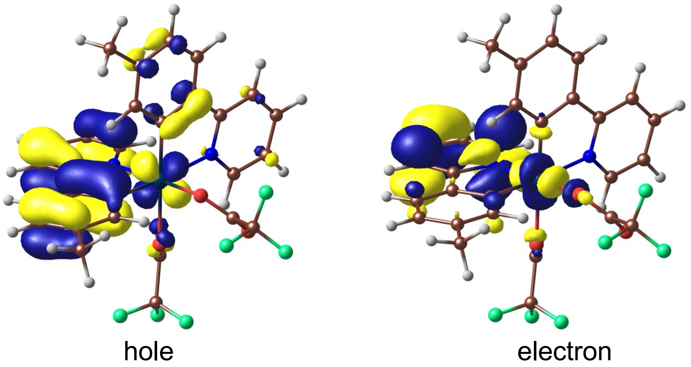

Figure S30. Natural transition orbitals of the T_4 excitation of complex **4** (0.03 e $bohr^{-3}$).

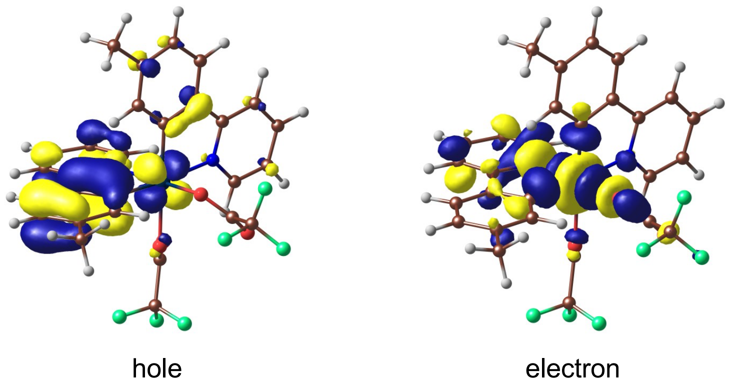

Figure S31. Natural transition orbitals of the T_5 excitation of complex **4** (0.03 e $bohr^{-3}$).

3.3. Complex 5

Table S8. Fragment contributions (%; from atomic orbital contributions) to the frontier orbitals of **5** in MeCN solution.

energy (a.u.)	number	L1	L2	L3	L4	Pt
–0.011	147 (LUMO+5)	30	27	0	6	36
–0.028	146 (LUMO+4)	14	84	0	0	2
–0.038	145 (LUMO+3)	84	15	0	0	1
–0.041	144 (LUMO+2)	25	10	15	2	47
–0.053	143 (LUMO+1)	1	97	0	0	1
–0.061	142 (LUMO)	95	0	0	1	3
–0.213	141 (HOMO)	11	1	1	84	2
–0.223	140 (HOMO–1)	3	91	0	2	3
–0.224	139 (HOMO–2)	84	4	0	8	4
–0.232	138 (HOMO–3)	3	3	81	11	2
–0.236	137 (HOMO–4)	39	17	11	25	8
–0.243	136 (HOMO–5)	20	36	5	33	6

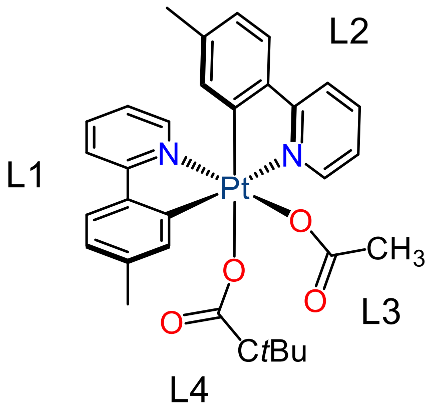

Figure S32. Ligand numbering in complex **5**.

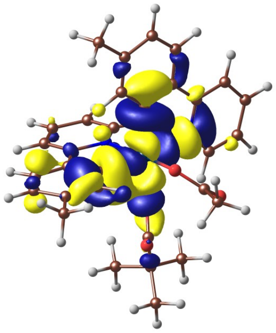

147 (LUMO+5)

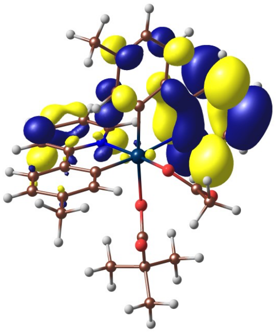

146 (LUMO+4)

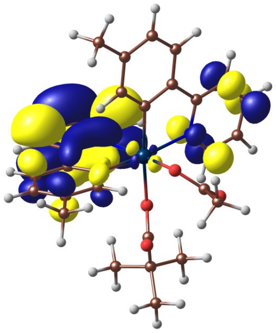

145 (LUMO+3)

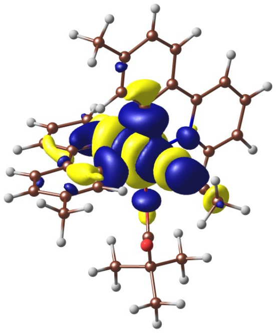

144 (LUMO+2)

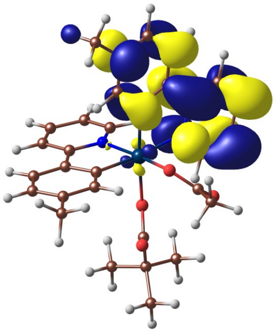

143 (LUMO+1)

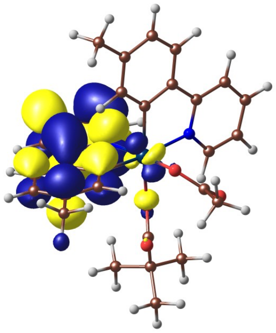

142 (LUMO)

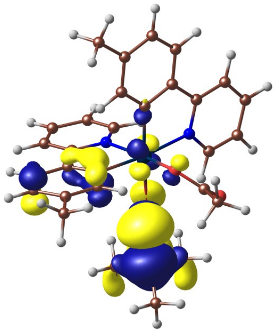

141 (HOMO)

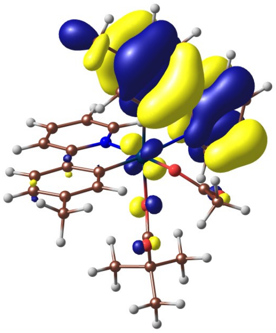

140 (HOMO–1)

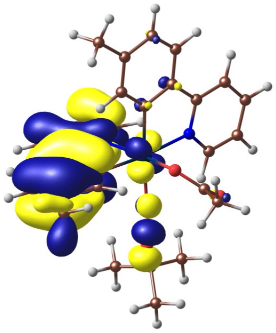

139 (HOMO–2)

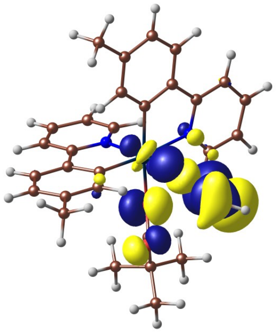

138 (HOMO–3)

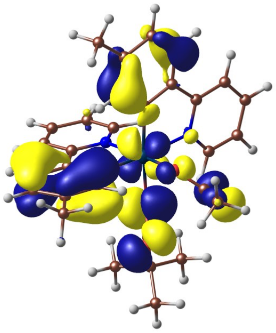

137 (HOMO–4)

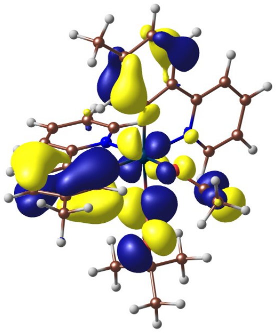

136 (HOMO–5)

Figure S33. Molecular orbital isosurfaces of **5** (0.03 e bohr^{-3}).

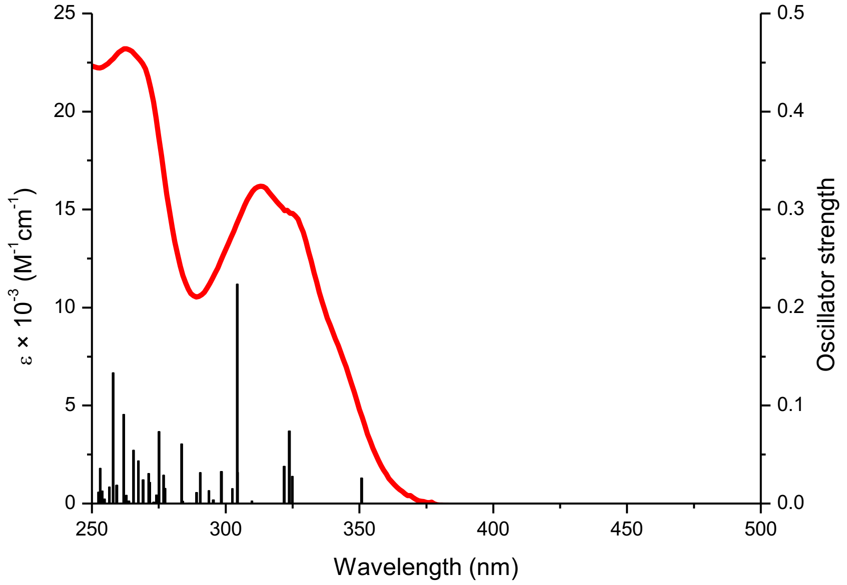

Figure S34. Calculated stick absorption spectrum of **5** compared with the experimental spectrum in MeCN solution (*ca.* 1×10^{-5} M) at 298 K.

Table S9. Selected vertical singlet excitations of **5** from TDDFT calculations at the ground state geometry in MeCN solution.

State	Monoexcitations	Coefficient (percentage)	ΔE/eV	λ/nm	Oscillator strength	Main character
S1	H → L	0.69533 (97%)	3.534	350.8	0.0261	LLCT (L4 → L1)
S2	H–2 → L<br>H–2 → L+2<br>H–1 → L<br>H → L+2	–0.36011 (26%)<br>0.17722 (6%)<br>–0.24047 (12%)<br>0.47225 (45%)	3.816	324.9	0.0279	LMCT (L4 → Pt)
S3	H–4 → L<br>H–3 → L<br>H–2 → L<br>H–1 → L<br>H–1 → L+2<br>H → L+1	0.1484 (4%)<br>–0.10302 (2%)<br>–0.4169 (35%)<br>0.4904 (48%)<br>–0.11656 (3%)<br>–0.12317 (3%)	3.829	323.8	0.0741	LC (L1) / LLCT (L2 → L1)
S4	H–2 → L<br>H–1 → L<br>H → L+1<br>H → L+2	–0.36317 (26%)<br>–0.25173 (13%)<br>0.41988 (35%)<br>–0.3327 (22%)	3.852	321.9	0.0381	LLCT (L4 → L1)
S5	H–2 → L<br>H–1 → L<br>H → L+1<br>H → L+2	0.1367 (4%)<br>0.2743 (15%)<br>0.54566 (60%)<br>0.30982 (19%)	3.854	321.7	0.0006	LLCT (L4 → L2)

S6	H–5 → L+2<br>H–4 → L<br>H–4 → L+2<br>H–3 → L<br>H–3 → L+2<br>H–1 → L<br>H–1 → L+2<br>H → L+2	–0.16394 (5%)<br>–0.20253 (8%)<br>0.21549 (9%)<br>0.24395 (12%)<br>–0.22608 (10%)<br>0.23984 (12%)<br>0.38877 (30%)<br>–0.12938 (3%)	4.002	309.8	0.0027	LMCT (L2 → Pt)
S7	H–4 → L<br>H–4 → L+2<br>H–3 → L<br>H–2 → L+2<br>H–1 → L+2	–0.31413 (20%)<br>–0.13828 (4%)<br>0.43902 (39%)<br>0.15684 (5%)<br>–0.35188 (25%)	4.072	304.5	0.032	LLCT (L3 → L1)
S8	H–2 → L+1<br>H–1 → L+1	0.11236 (3%)<br>0.65256 (85%)	4.075	304.3	0.2238	LC (L2)
S9	H–3 → L<br>H–2 → L+1<br>H–2 → L+2<br>H → L+2	–0.17942 (6%)<br>–0.19945 (8%)<br>0.58236 (68%)<br>–0.16962 (6%)	4.099	302.5	0.0153	LMCT (L1 → Pt)
S10	H–4 → L<br>H–3 → L<br>H–2 → L+1<br>H–2 → L+2	0.10783 (2%)<br>0.11602 (3%)<br>0.62095 (77%)<br>0.20655 (9%)	4.126	300.5	0.0002	LLCT (L1 → L2)

Table S10. Selected vertical triplet excitations of **5** from TDDFT calculations at the ground state geometry in MeCN solution.

State	Monoexcitations	Coefficient (percentage)	ΔE/eV	λ/nm	main character
T1	H–4 → L<br>H–2 → L<br>H–2 → L+3<br>H → L	0.10083 (2%)<br>0.59206 (70%)<br>0.16891 (6%)<br>0.17353 (6%)	2.886	429.6	LC (L1)
T2	H–4 → L+1<br>H–2 → L+1<br>H–1 → L+1<br>H–1 → L+4	–0.10271 (2%)<br>0.11326 (3%)<br>0.61551 (76%)<br>–0.17079 (6%)	2.957	419.3	LC (L2)
T3	H–5 → L<br>H–4 → L<br>H–2 → L+3<br>H → L	–0.10558 (2%)<br>–0.21804 (10%)<br>–0.10144 (2%)<br>0.6112 (75%)	3.417	362.8	LLCT (L4 → L1)
T4	H–8 → L+2<br>H–5 → L+2<br>H–2 → L<br>H–2 → L+2<br>H–2 → L+3<br>H–1 → L<br>H–1 → L+2<br>H → L+2<br>H → L+3	0.13669 (4%)<br>–0.18002 (6%)<br>0.14422 (4%)<br>0.18537 (7%)<br>–0.30201 (18%)<br>–0.16955 (6%)<br>0.14154 (4%)<br>0.34881 (24%)<br>–0.11202 (3%)	3.620	342.5	LMCT (L1/L4 → Pt)
T5	H–13 → L+3	–0.12445 (3%)	3.632	341.4	LMCT (L1/L4 → Pt)
	H–11 → L+3	0.12441 (3%)			
	H–8 → L+2	0.10028 (2%)			
	H–5 → L+2	–0.13336 (4%)			
	H–2 → L	–0.19169 (7%)			
	H–2 → L+2	0.21804 (10%)			
	H–2 → L+3	0.36329 (26%)			
	H–2 → L+4	–0.12706 (3%)			
	H → L	0.10491 (2%)			
	H → L+2	0.29249 (17%)			
	H → L+3	0.13728 (4%)			

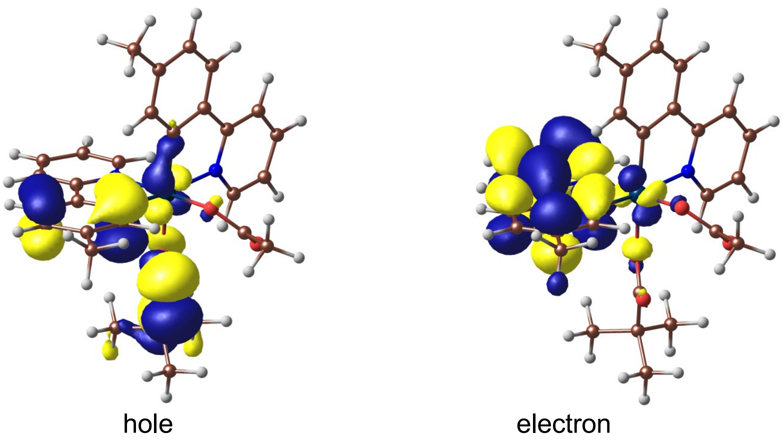

Figure S35. Natural transition orbitals of the T_3 excitation of complex **5** (0.03 e bohr^{-3}).

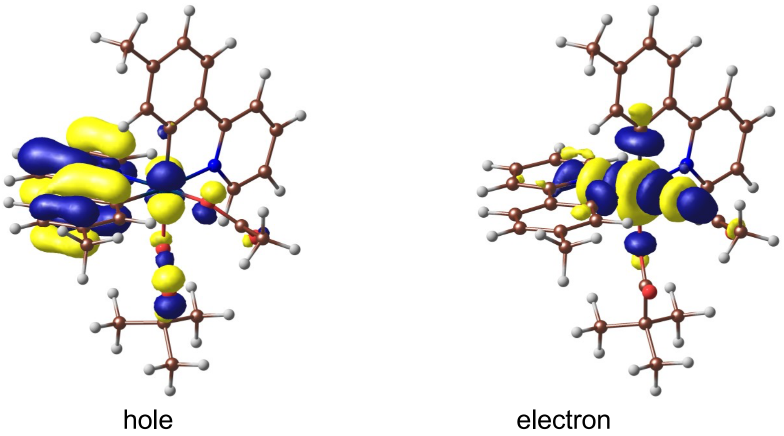

Figure S36. Natural transition orbitals of the T_4 excitation of complex **5** (0.03 e bohr^{-3}).

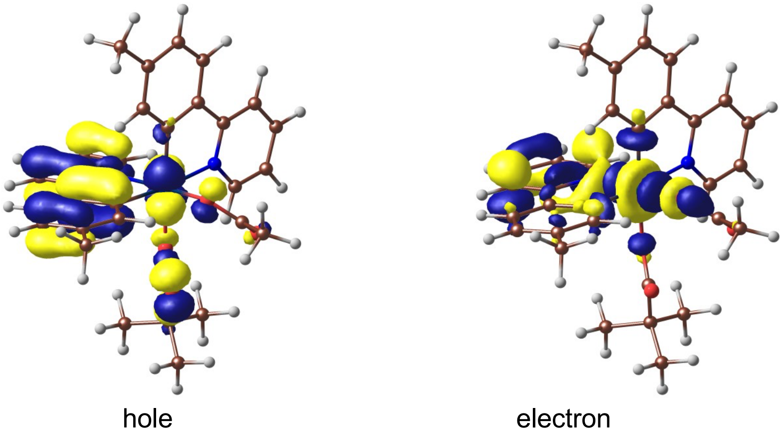

Figure S37. Natural transition orbitals of the T_5 excitation of complex **5** (0.03 e bohr^{-3}).

3.4. Complex 6

Table S11. Fragment contributions (%; from atomic orbital contributions) to the frontier orbitals of **6** in MeCN solution.

energy (a.u.)	number	L1	L2	L3	L4	Pt
–0.019	147 (LUMO+5)	31	27	0	5	36
–0.030	146 (LUMO+4)	0	84	0	0	2
–0.040	145 (LUMO+3)	83	15	0	0	2
–0.047	144 (LUMO+2)	26	13	15	2	45
–0.055	143 (LUMO+1)	1	97	0	0	1
–0.064	142 (LUMO)	92	0	0	1	5
–0.226	141 (HOMO)	68	25	0	1	5
–0.226	140 (HOMO–1)	24	72	1	0	2
–0.237	139 (HOMO–2)	0	4	92	2	1
–0.239	138 (HOMO–3)	70	3	0	21	5
–0.246	137 (HOMO–4)	2	78	3	4	10
–0.254	136 (HOMO–5)	18	9	7	64	3

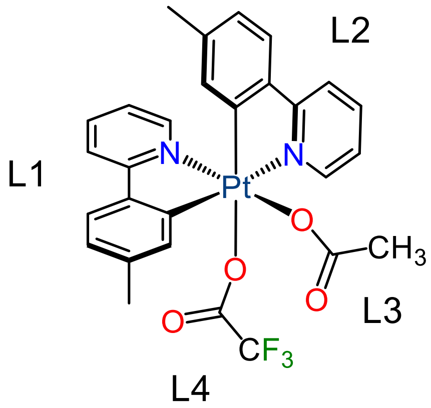

Figure S38. Ligand numbering in complex **6**.

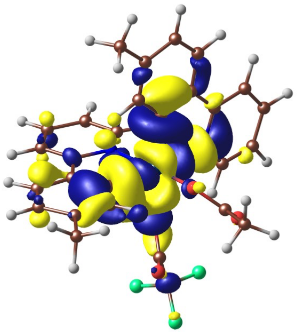

147 (LUMO+5)

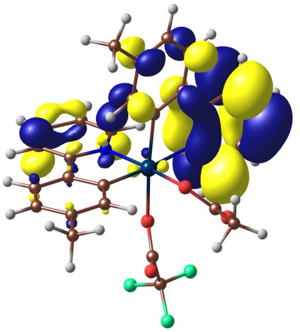

146 (LUMO+4)

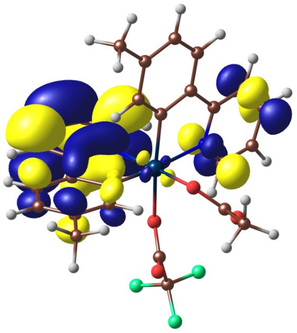

145 (LUMO+3)

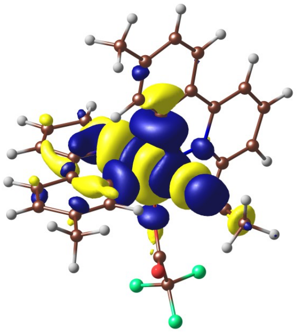

144 (LUMO+2)

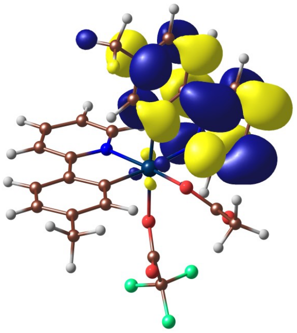

143 (LUMO+1)

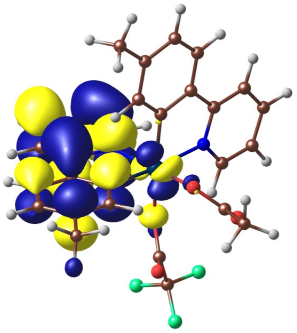

142 (LUMO)

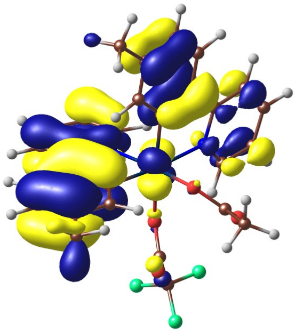

141 (HOMO)

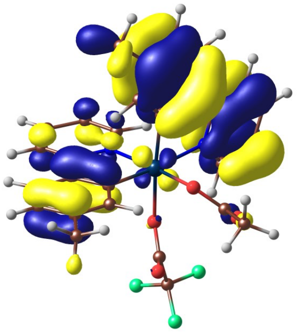

140 (HOMO–1)

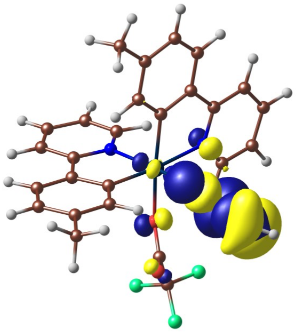

139 (HOMO–2)

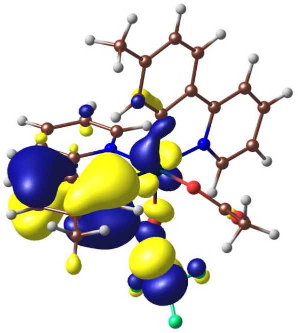

138 (HOMO–3)

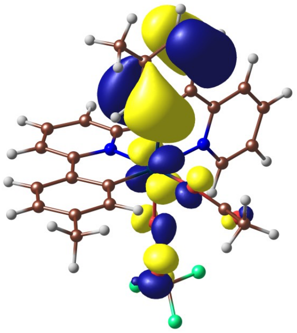

137 (HOMO–4)

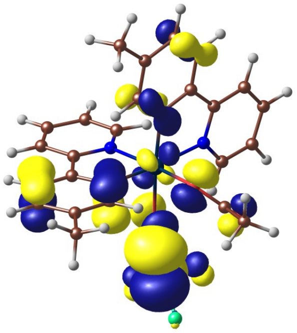

136 (HOMO–5)

Figure S39. Molecular orbital isosurfaces of **6** (0.03 e bohr^{-3}).

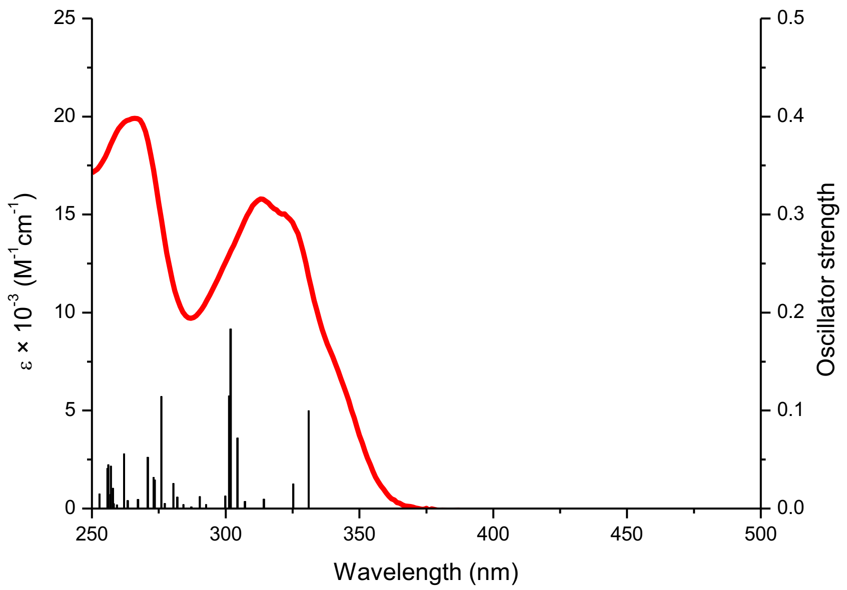

Figure S40. Calculated stick absorption spectrum of **6** compared with the experimental spectrum in MeCN solution (*ca.* 1 × 10^{-5} M) at 298 K.

Table S12. Selected vertical singlet excitations of **6** from TDDFT calculations at the ground state geometry in MeCN solution.

State	Monoexcitations	Coefficient (percentage)	ΔE/eV	λ/nm	Oscillator strength	Main character
S1	H–3 → L<br>H → L<br>H → L+2	0.10728 (2%)<br>0.6417 (82%)<br>–0.2012 (8%)	3.750	330.6	0.1009	LC (L1)
S2	H–4 → L+2<br>H–1 → L<br>H–1 → L+2<br>H → L+2	–0.10016 (2%)<br>0.64597 (83%)<br>–0.18372 (7%)<br>–0.11202 (3%)	3.817	324.8	0.0264	LLCT (L2 → L1)
S3	H–4 → L+2<br>H–3 → L<br>H–1 → L<br>H → L<br>H → L+2	0.17732 (6%)<br>0.1028 (2%)<br>0.14543 (4%)<br>0.22375 (10%)<br>0.58691 (69%)	3.950	313.9	0.0107	LMCT (L1 →Pt)
S4	H–4 → L<br>H–4 → L+2<br>H–3 → L+2<br>H–1 → L<br>H–1 → L+2<br>H → L+2	–0.10116 (2%)<br>0.18434 (7%)<br>–0.13917 (4%)<br>0.19779 (8%)<br>0.59254 (70%)<br>–0.10968 (2%)	4.042	306.8	0.0084	LMCT (L2 → Pt)
S5	H–3 → L<br>H–1 → L+1<br>H → L+1	–0.2689 (14%)<br>0.21829 (10%)<br>0.58421 (68%)	4.078	304.0	0.0733	LLCT (L1 → L2)
S6	H–3 → L	0.18984 (7%)	4.114	301.4	0.1844	LC (L2)

	H–1 → L+1<br>H → L+1	0.62458 (78%)<br>–0.1632 (5%)				
S7	H–3 → L<br>H–2 → L<br>H–2 → L+2<br>H → L+1<br>H → L+3	0.52127 (54%)<br>0.20156 (8%)<br>–0.10623 (2%)<br>0.326 (21%)<br>0.11458 (3%)	4.120	300.9	0.1161	LC (L1)/ LLCT (L4 → L1)
S8	H–3 → L<br>H–2 → L<br>H–2 → L+2<br>H–1 → L+1	–0.19115 (7%)<br>0.57501 (66%)<br>–0.30389 (18%)<br>0.10499 (2%)	4.141	299.4	0.0141	LLCT (L3 → L1)
S9	H–6 → L+2<br>H–4 → L<br>H–4 → L+2<br>H–3 → L+2<br>H–1 → L+2<br>H → L+2	–0.18381 (7%)<br>0.48787 (48%)<br>–0.28151 (16%)<br>0.10123 (2%)<br>0.26482 (14%)<br>0.17882 (6%)	4.242	292.3	0.0053	LLCT (L2 →L1)
S10	H–2 → L<br>H–2 → L+1<br>H–2 → L+2	0.10166 (2%)<br>0.65932 (87%)<br>0.1752 (6%)	4.276	289.9	0.0135	LLCT (L3 → L1)

Table S13. Selected vertical triplet excitations of **6** from TDDFT calculations at the ground state geometry in MeCN solution.

State	Monoexcitations	Coefficient (Percentage)	ΔE/eV	λ/nm	Main character
T1	H–3 → L<br>H–3 → L+7<br>H–1 → L<br>H → L<br>H → L+3	–0.11564 (3%)<br>0.10092 (2%)<br>–0.29532 (17%)<br>0.54716 (60%)<br>0.14719 (4%)	2.875	431.3	LC (L1)
T2	H–4 → L+6<br>H–1 → L+1<br>H–1 → L+4<br>H → L+1	–0.12881 (3%)<br>0.55025 (61%)<br>–0.15753 (5%)<br>0.30988 (19%)	2.957	419.4	LC (L2)
T3	H–11 → L+3<br>H–3 → L<br>H–3 → L+3<br>H–1 → L<br>H–1 → L+3<br>H → L<br>H → L+2<br>H → L+3<br>H → L+4	–0.12072 (3%)<br>0.37245 (28%)<br>0.17596 (6%)<br>–0.10957 (2%)<br>0.16911 (6%)<br>0.23626 (11%)<br>–0.21226 (9%)<br>–0.28768 (17%)<br>0.10214 (2%)	3.589	345.5	LC (L1) / LMCT (L1 → Pt)
T4	H–6 → L+2<br>H–4 → L<br>H–4 → L+2<br>H–1 → L	0.1561 (5%)<br>–0.14024 (4%)<br>0.26497 (14%)<br>–0.24942 (12%)	3.644	340.3	LMCT (L1/L3 → Pt)

	H–1 → L+3<br>H → L+2<br>H → L+3	0.1108 (2%)<br>0.41282 (34%)<br>–0.2037 (8%)			
T5	H–11 → L+3<br>H–5 → L<br>H–4 → L+2<br>H–3 → L<br>H–1 → L+3<br>H → L+2<br>H → L+3	0.11833 (3%)<br>–0.13711 (4%)<br>0.10653 (2%)<br>0.49137 (48%)<br>–0.17061 (6%)<br>0.11437 (3%)<br>0.2955 (17%)	3.677	337.2	LC (L1)

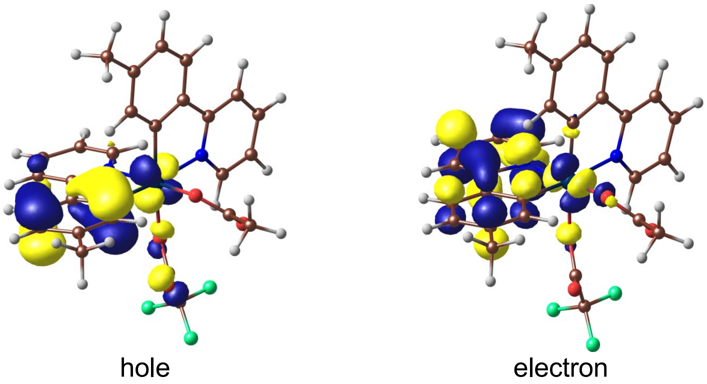

Figure S41. Natural transition orbitals of the T_3 excitation of complex **6** (0.03 e bohr^{-3}).

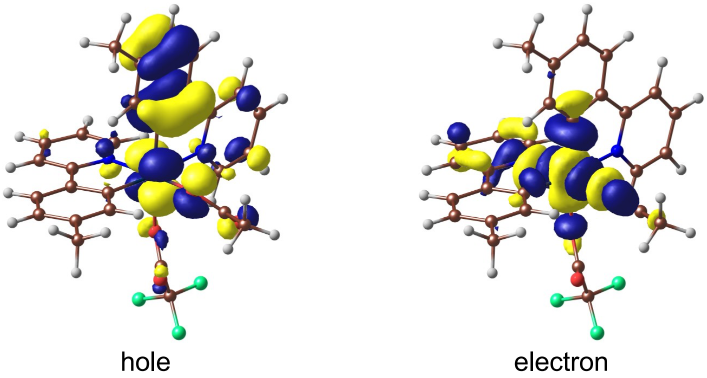

Figure S42. Natural transition orbitals for the T_4 excitation of complex **6** (0.03 e bohr^{-3}).

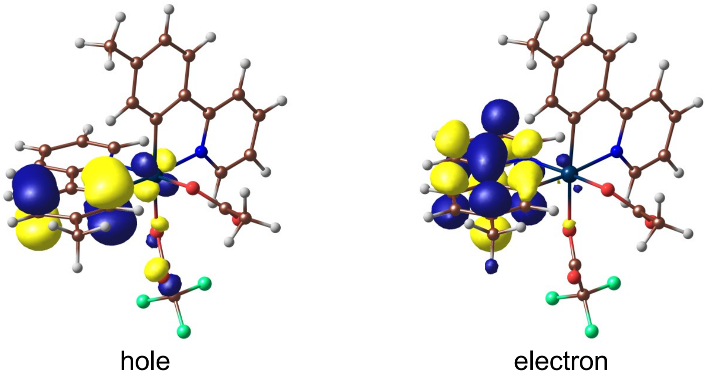

Figure S43. Natural transition orbitals for the T_5 excitation of complex **6** (0.03 e bohr^{-3}).

3.5. Complex 9

Table S14. Fragment contributions (%; from atomic orbital contributions) to the frontier orbitals of **9** in MeCN solution.

energy (a.u.)	number	L1	L2	L3	L4	Pt
–0.018	147 (LUMO+5)	26	29	0	6	36
–0.029	146 (LUMO+4)	10	87	0	0	1
–0.039	145 (LUMO+3)	87	11	0	0	2
–0.051	144 (LUMO+2)	31	13	12	1	43
–0.055	143 (LUMO+1)	2	94	1	0	3
–0.065	142 (LUMO)	91	0	0	1	6
–0.224	141 (HOMO)	9	83	0	5	3
–0.226	140 (HOMO–1)	52	14	0	33	2
–0.229	139 (HOMO–2)	37	2	0	59	2
–0.244	138 (HOMO–3)	9	55	1	24	10
–0.245	137 (HOMO–4)	75	6	0	13	6
–0.251	136 (HOMO–5)	2	34	7	54	3

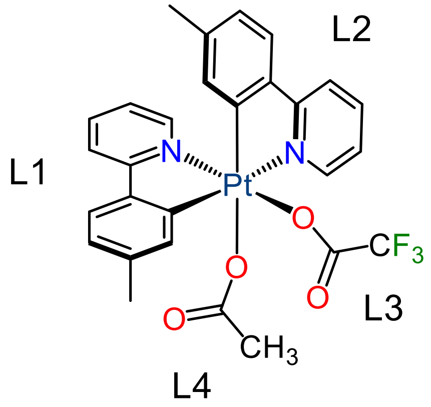

Figure S44. Ligand numbering in complex **9**.

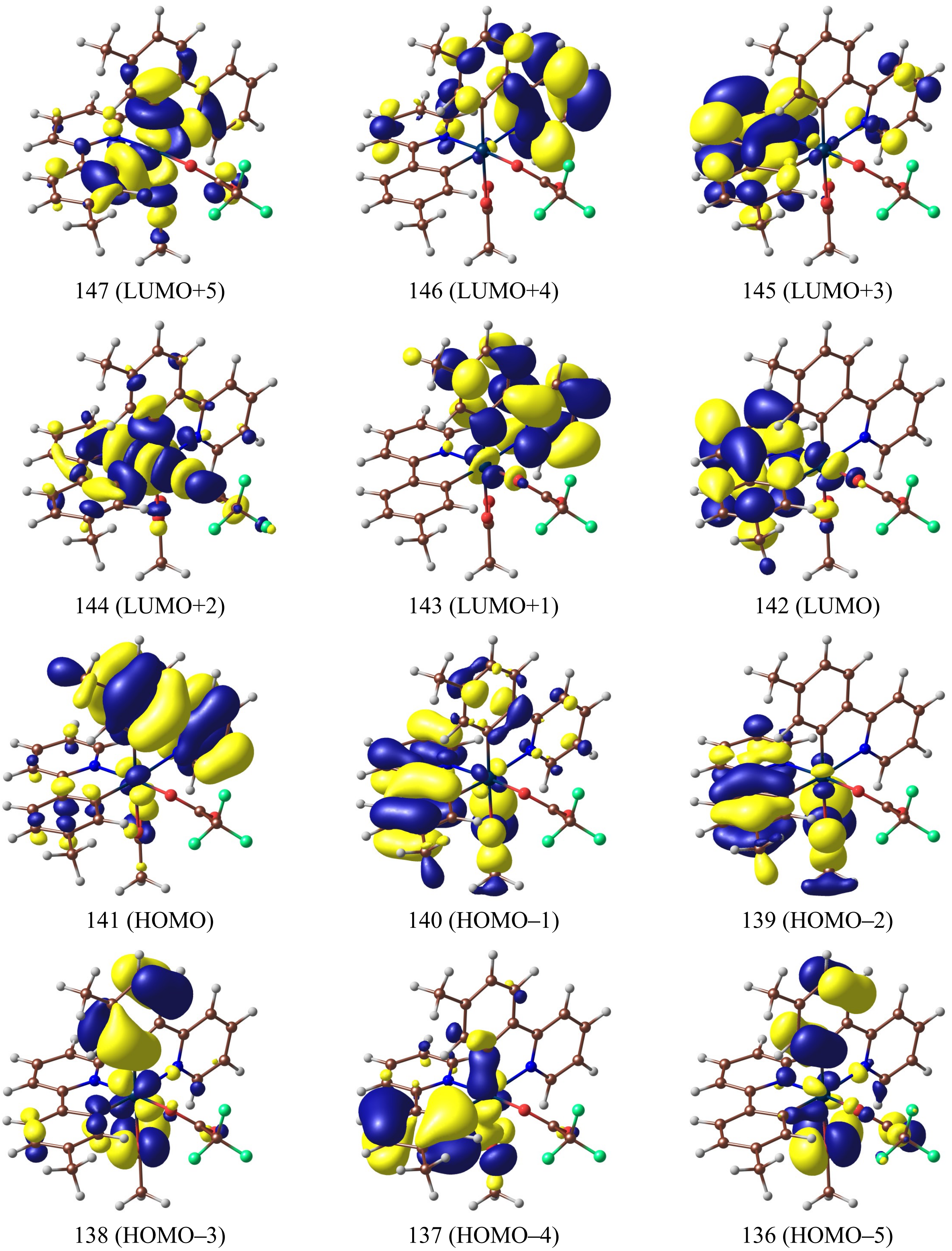

Figure S45. Molecular orbital isosurfaces of **9** (0.03 e bohr^{-3}).

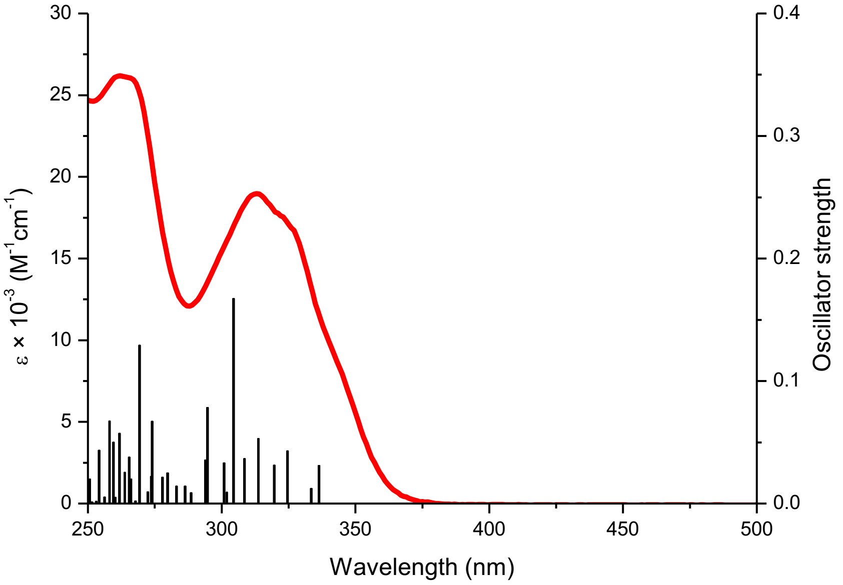

Figure S46. Calculated stick absorption spectrum of **9** compared with the experimental spectrum in MeCN solution (*ca.* 1 × 10^{-5} M) at 298 K.

Table S15. Selected vertical singlet excitations of **9** from TDDFT calculations at the ground state geometry in MeCN solution.

State	Monoexcitations	Coefficient (probability)	ΔE/eV	λ/nm	Oscillator strength	Main character
S1	H–3 → L+2<br>H–1 → L<br>H → L<br>H → L+2	–0.10991 (2%)<br>0.23387 (11%)<br>0.53585 (57%)<br>–0.29975 (18%)	3.686	336.4	0.0311	LLCT (L2 → L1)
S2	H–2 → L<br>H–2 → L+2<br>H–1 → L<br>H–1 → L+2<br>H → L+2	0.35536 (25%)<br>–0.12384 (3%)<br>0.53083 (56%)<br>–0.1344 (4%)<br>0.15797 (5%)	3.718	333.5	0.0125	LC (L1) / LLCT (L4 → L1)
S3	H–2 → L<br>H–2 → L+2<br>H–1 → L<br>H–1 → L+2<br>H → L	0.47674 (45%)<br>–0.19331 (7%)<br>–0.27894 (16%)<br>0.25158 (13%)<br>0.26343 (14%)	3.820	324.6	0.0431	LC (L1) / LLCT (L4 → L1)
S4	H–3 → L+2<br>H–2 → L<br>H–2 → L+2<br>H → L<br>H → L+1<br>H → L+2	0.15462 (5%)<br>–0.2795 (16%)<br>–0.12427 (3%)<br>0.34815 (24%)<br>–0.17336 (6%)<br>0.44691 (40%)	3.879	319.7	0.0313	LMCT (L2 → Pt)
S5	H–3 → L+2	–0.15466 (5%)	3.952	313.7	0.0531	LMCT (L1/L4 → Pt)

	H–2 → L<br>H–1 → L<br>H–1 → L+1<br>H–1 → L+2	–0.11738 (3%)<br>0.25184 (13%)<br>–0.18397 (7%)<br>0.56898 (65%)				
S6	H–2 → L<br>H–2 → L+1<br>H–2 → L+2<br>H → L+1<br>H → L+2	0.16582 (5%)<br>–0.19312 (7%)<br>0.57702 (67%)<br>–0.20661 (9%)<br>0.18562 (7%)	4.019	308.5	0.0367	LMCT (L1/L4 → Pt)
S7	H–2 → L+2<br>H → L+1<br>H → L+2	0.1221 (3%)<br>0.60434 (73%)<br>0.26748 (14%)	4.073	304.4	0.1674	LC (L2)
S8	H–5 → L<br>H–5 → L+2<br>H–3 → L<br>H–3 → L+2<br>H–1 → L+1<br>H–1 → L+2<br>H → L+2	0.17025 (6%)<br>–0.10379 (2%)<br>0.50498 (51%)<br>–0.30476 (19%)<br>0.14874 (4%)<br>–0.13408 (4%)<br>0.19876 (8%)	4.107	301.9	0.0094	LLCT (L2/L4 → L1)
S9	H–2 → L+1<br>H–2 → L+2<br>H–1 → L+1<br>H–1 → L+2	0.30382 (18%)<br>0.12379 (3%)<br>0.57339 (66%)<br>0.19823 (8%)	4.120	300.9	0.0333	LLCT (L1/L4 → L2)
S10	H–4 → L<br>H–2 → L+1<br>H–2 → L+2<br>H–1 → L+1	0.43845 (38%)<br>–0.42145 (36%)<br>–0.13655 (4%)<br>0.23048 (11%)	4.207	294.7	0.0784	LC (L1)

Table S16. Selected vertical triplet excitations of **9** from TDDFT calculations at the ground state geometry in MeCN solution.

**state**	**monoexcitations**	**Coefficient (Percentage)**	**ΔE/eV**	**λ/nm**	**main character**
T1	H–2 → L<br>H–2 → L+3<br>H–1 → L<br>H–1 → L+3<br>H → L	–0.38243 (29%)<br>–0.10295 (2%)<br>0.44596 (40%)<br>0.134475 (4%)<br>0.21 (9%)	2.879	430.7	LC (L1)
T2	H–1 → L+1<br>H → L+1<br>H → L+2<br>H → L+4	–0.22087 (10%)<br>0.58092 (67%)<br>0.11165 (2%)<br>0.16609 (6%)	2.952	420.0	LC (L2)
T3	H–8 → L+2<br>H–4 → L<br>H–4 → L+2<br>H–3 → L<br>H–3 → L+2	–0.10761 (2%)<br>–0.11158 (2%)<br>0.13912 (4%)<br>–0.11948 (3%)<br>0.23403 (11%)	3.530	351.3	LMCT (L2/L4 → Pt)

	H–2 → L<br>H–2 → L+2<br>H–1 → L<br>H → L<br>H → L+2	0.10363 (2%)<br>–0.24586 (12%)<br>0.10297 (2%)<br>–0.24437 (12%)<br>0.4177 (35%)			
T4	H–11 → L+3<br>H–4 → L<br>H–4 → L+3<br>H–3 → L<br>H–2 → L<br>H–2 → L+3<br>H–1 → L<br>H–1 → L+2<br>H–1 → L+3<br>H → L+3	–0.15658 (5%)<br>0.16634 (6%)<br>0.13352 (4%)<br>–0.1316 (3%)<br>0.1839 (7%)<br>0.14663 (4%)<br>0.40176 (32%)<br>–0.16167 (5%)<br>–0.28968 (17%)<br>–0.11225 (3%)	3.588	345.6	LC (L1) /LLCT (L4 → L1)
T5	H–4 → L+2<br>H–3 → L<br>H–3 → L+2<br>H–2 → L<br>H–2 → L+2<br>H–1 → L+2<br>H → L	0.16007 (5%)<br>0.12003 (3%)<br>–0.1883 (7%)<br>0.33862 (23%)<br>–0.23191 (11%)<br>0.30848 (19%)<br>0.30196 (18%)	3.711	334.1	LMCT (L2/L4 → Pt)

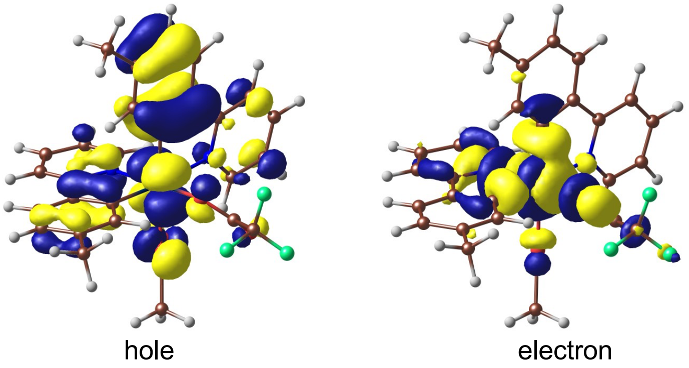

Figure S47. Natural transition orbitals of the T_3 excitation of complex **9** (0.03 e bohr^{-3}).

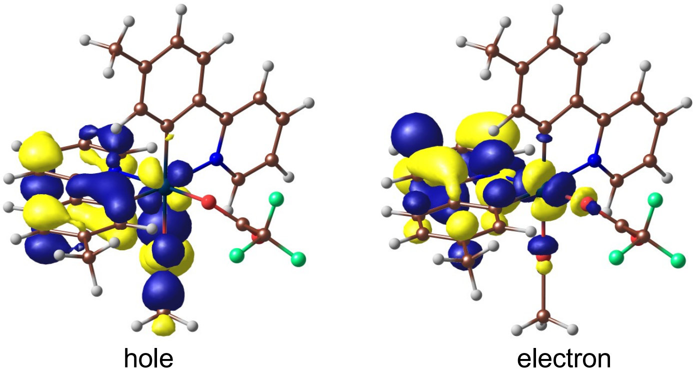

Figure S48. Natural transition orbitals of the T_4 excitation of complex **9** (0.03 e bohr^{-3}).

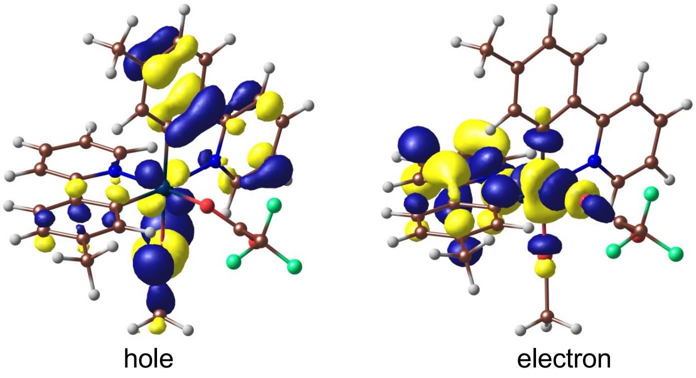

Figure S49. Natural transition orbitals of the T_5 excitation of complex **9** (0.03 e bohr^{-3}).

3.6. Complex 11

Table S17. Fragment contributions (%; from atomic orbital contributions) to the frontier orbitals of **11** in MeCN solution.

energy (a.u.)	number	L1	L2	L3	L4	Pt
–0.012	147 (LUMO+5)	30	27	0	6	36
–0.028	146 (LUMO+4)	14	84	0	0	2
–0.038	145 (LUMO+3)	84	15	0	0	1
–0.042	144 (LUMO+2)	25	10	15	2	47
–0.053	143 (LUMO+1)	1	97	0	0	1
–0.061	142 (LUMO)	95	0	0	1	3
–0.216	141 (HOMO)	19	2	0	76	3
–0.223	140 (HOMO–1)	5	89	3	1	5
–0.224	139 (HOMO–2)	77	5	0	14	3
–0.229	138 (HOMO–3)	0	3	87	8	2
–0.237	137 (HOMO–4)	41	18	4	28	9
–0.243	136 (HOMO–5)	22	33	5	33	6

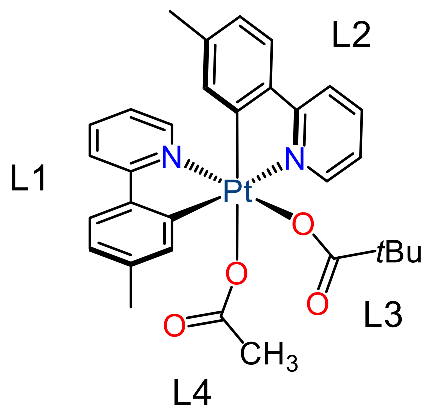

Figure S50. Ligand numbering in complex **11**.

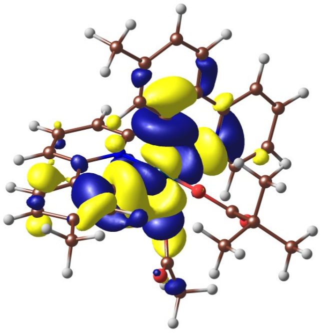

147 (LUMO+5)

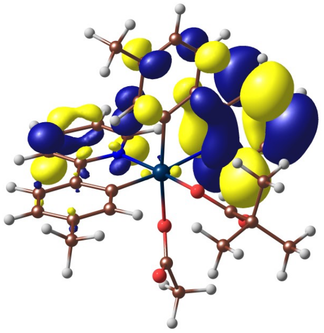

146 (LUMO+4)

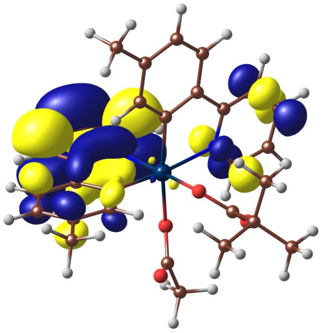

145 (LUMO+3)

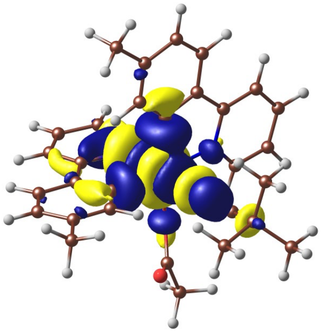

144 (LUMO+2)

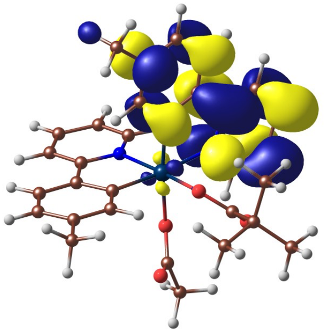

143 (LUMO+1)

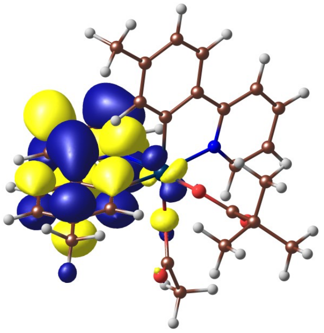

142 (LUMO)

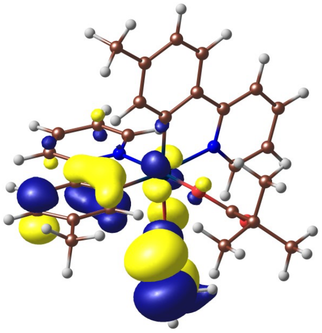

141 (HOMO)

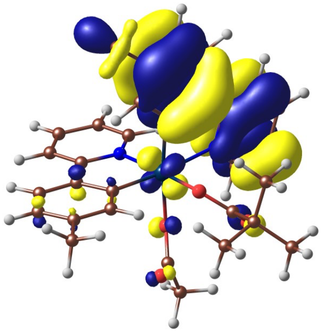

140 (HOMO–1)

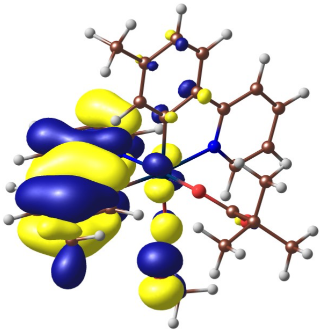

139 (HOMO–2)

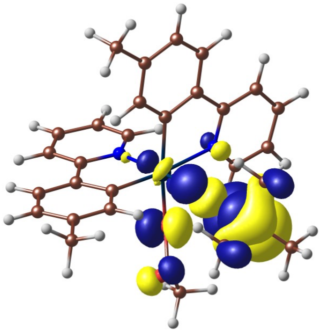

138 (HOMO–3)

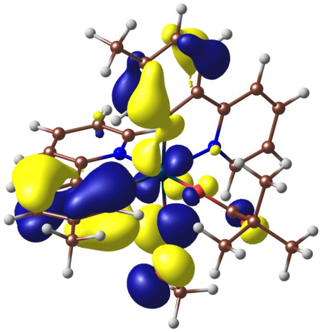

137 (HOMO–4)

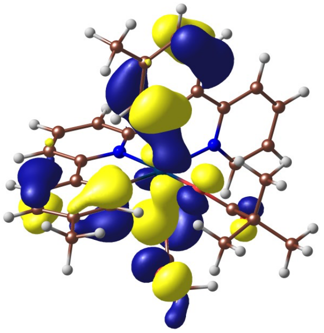

136 (HOMO–5)

Figure S51. Molecular orbital isosurfaces of **11** (0.03 e $bohr^{-3}$).

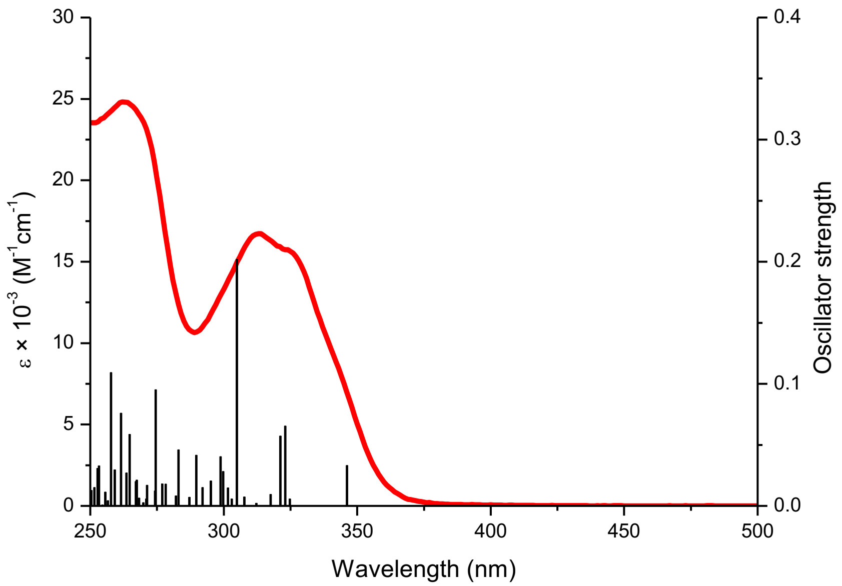

Figure S52. Calculated stick absorption spectrum of **11** compared with the experimental spectrum in MeCN solution (*ca.* 1×10^{-5} M) at 298 K.

Table S18. Selected vertical singlet excitations of **11** from TDDFT calculations at the ground state geometry in MeCN solution.

State	Monoexcitations	Coefficient (percentage)	ΔE/eV	λ/nm	Oscillator strength	Main character
S1	H → L	0.69183 (96%)	3.582	346.2	0.0332	LLCT (L4 → L1)
S2	H–5 → L+2	0.10906 (2%)	3.819	324.7	0.0058	LMCT (L4 → Pt)
	H–2 → L	–0.21731 (9%)				
	H–2 → L+2	0.18803 (7%)				
	H–1 → L	–0.32974 (22%)				
	H–1 → L+2	0.12372 (3%)				
	H → L+2	0.47927 (46%)				
S3	H–4 → L	0.12564 (3%)	3.839	323.0	0.0656	LLCT (L2 → L1)
	H–2 → L	–0.3823 (29%)				
	H–1 → L	0.49685 (49%)				
	H–1 → L+2	–0.11617 (3%)				
	H → L+2	0.1963 (8%)				
S4	H–2 → L	0.50811 (52%)	3.860	321.2	0.0574	LC (L1) / LMCT (L1/L4 → Pt)
	H–1 → L	0.24985 (12%)				
	H → L+2	0.38841 (30%)				
S5	H → L+1	0.69673 (97%)	3.905	317.5	0.0096	LLCT (L1/L4 → L2)
S6	H–5 → L+2	0.15494 (5%)	3.971	312.2	0.0023	LMCT (L2/L3 → Pt)
	H–4 → L	–0.14742 (4%)				
	H–4 → L+2	0.21411 (9%)				
	H–3 → L	–0.31889 (20%)				
	H–3 → L+2	0.25349 (13%)				
	H–1 → L	0.27215 (15%)				
	H–1 → L+2	0.34426 (24%)				

	H → L+2	–0.13053 (3%)				
S7	H–5 → L+2<br>H–4 → L<br>H–4 → L+2<br>H–3 → L<br>H–1 → L+2	0.10617 (2%)<br>0.11762 (3%)<br>0.15095 (5%)<br>0.54175 (59%)<br>0.37422 (28%)	4.031	307.6	0.0074	LLCT (L3 → L1)
S8	H–3 → L+1<br>H–1 → L+1	–0.13533 (4%)<br>0.65145 (85%)	4.067	304.9	0.2021	LC (L2)
S9	H–2 → L+1<br>H–2 → L+2<br>H → L+2	–0.11749 (3%)<br>0.62801 (79%)<br>–0.19915 (8%)	4.093	302.9	0.0059	LMCT (L1 → Pt)
S10	H–3 → L+1<br>H–2 → L+1	0.59158 (70%)<br>0.3311 (22%)	4.111	301.6	0.015	LLCT (L3 → L2)

Table S19. Selected vertical triplet excitations of **11** from TDDFT calculations at the ground state geometry in MeCN solution.

State	Monoexcitations	Coefficient (Percentage)	ΔE/eV	λ/nm	Main character
T1	H–2 → L<br>H–2 → L+3<br>H–1 → L<br>H → L	0.56243 (63%)<br>0.16673 (6%)<br>–0.11636 (3%)<br>0.24628 (12%)	2.885	429.8	LC (L1)
T2	H–2 → L+1<br>H–1 → L+1<br>H–1 → L+4	0.12884 (3%)<br>0.6094 (74%)<br>–0.16961 (6%)	2.956	419.5	LC (L2)
T3	H–5 → L<br>H–4 → L<br>H–2 → L<br>H–2 → L+3<br>H → L	0.1255 (3%)<br>–0.24937 (12%)<br>–0.11071 (2%)<br>–0.13216(3%)<br>0.57495 (66%)	3.449	359.5	LLCT (L4 → L1)
T4	H–8 → L+2<br>H–7 → L+2<br>H–5 → L+2<br>H–3 → L+2<br>H–2 → L+2<br>H–2 → L+3<br>H–1 → L<br>H–1 → L+2<br>H → L+2	–0.12014 (3%)<br>0.12058 (3%)<br>0.22444 (10%)<br>0.11912 (3%)<br>0.27162 (15%)<br>–0.12632 (3%)<br>–0.13013 (3%)<br>0.14006 (4%)<br>0.44611 (40%)	3.603	344.1	LMCT (L3/L4 → Pt)

T5	H–13 → L+3<br>H–12 → L+3<br>H–2 → L<br>H–2 → L+3<br>H–2 → L+4<br>H–1 → L+3<br>H → L<br>H → L+2<br>H → L+3	–0.15315 (5%)<br>–0.14724 (4%)<br>–0.24252 (12%)<br>0.42998 (37%)<br>0.13915 (4%)<br>–0.11124 (2%)<br>0.10679 (2%)<br>0.11805 (3%)<br>0.23224 (11%)	3.627	341.8	LC (L1)

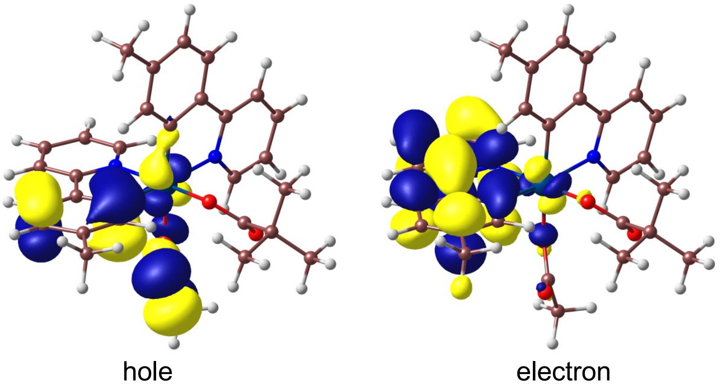

Figure S53. Natural transition orbitals of the T_3 excitation of complex **11** (0.03 e bohr^{-3}).

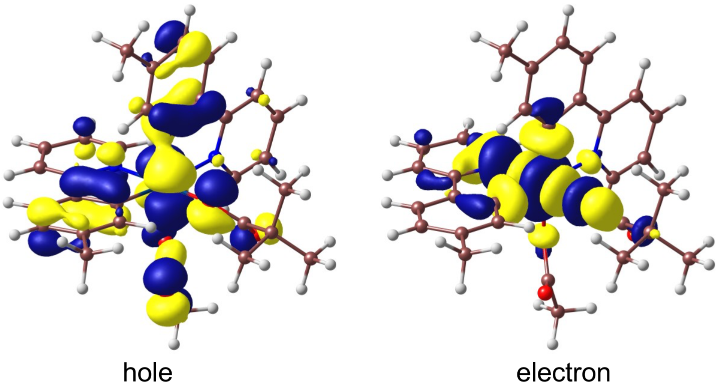

Figure S54. Natural transition orbitals of the T_4 excitation of complex **11** (0.03 e bohr^{-3}).

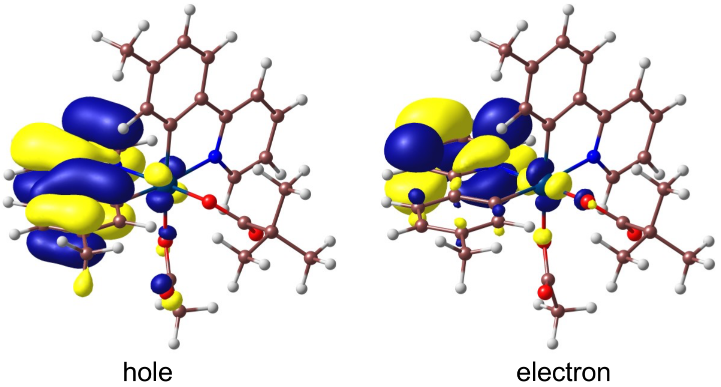

Figure S55. Natural transition orbitals of the T_5 excitation of complex **11** (0.03 e bohr^{-3}).

Table S20. Energies, free energies, enthalpies and entropies of the optimized structures of **2**, **4**, **5**, **6**, **9** and **11**.[a]

	E_0 [b]	ZPE [c]	G [d]	H [e]	S [f]
**2**	-1611.847247	-1611.881376	-1611.950574	-1611.846303	219.457
**4**	-2207.302964	-2207.340865	-2207.417039	-2207.302020	242.078
**5**	-1729.706073	-1729.744162	-1729.818588	-1729.705128	238.795
**6**	-1909.577854	-1909.613784	-1909.686209	-1909.576940	229.975
**9**	-1909.574058	-1909.610072	-1909.682787	-1909.573114	230.825
**11**	-1729.705534	-1729.743429	-1729.817310	-1729.704590	237.240

[a] Thermal corrections from vibrational calculations at 298.15 K. [b] Electronic energy (Hartrees). [c] Sum of electronic and zero-point energies (Hartrees). [d] Free Energy (Hartrees). [e] Enthalpy (Hartrees). [f] Entropy (cal mol^{-1} K^{-1}).

Table S21. Cartesian coordinates (Å) of the optimized structures at the B3LYP(6-31G**+LANL2DZ) level.

2

Pt	-0.513063037	0.024634002	-0.021258002
C	-0.156515011	1.169955085	-1.643324116
C	-0.229999017	0.757713054	-2.967651212
C	0.059287004	1.650007117	-4.015656289
C	0.420692030	2.969115212	-3.697420267
C	0.501016036	3.391580244	-2.373383171
C	0.213934016	2.495172178	-1.332195093
C	0.262940019	2.831250204	0.090160006
C	0.604102043	4.075854291	0.633028046
C	0.606277046	4.252076306	2.011164144
C	0.272259020	3.180358230	2.842442202
C	-0.050397004	1.963044141	2.261323163
H	-0.517040037	-0.264451019	-3.193763232
H	0.641170047	3.670497264	-4.497611321
H	0.783773054	4.417471316	-2.156322157
H	0.863188060	4.894231352	-0.027988002
H	0.866360065	5.216967375	2.435039174
H	0.262696019	3.277006237	3.921903284
H	-0.306173022	1.095586081	2.853098207
N	-0.057789004	1.802599128	0.927170066
C	-2.504364182	0.432243031	0.085851006
C	-3.207263231	1.255544092	-0.793876059
C	-4.590345332	1.464990108	-0.662245049
C	-5.266482379	0.819736057	0.382791028
C	-4.581327331	-0.002942000	1.270005093
C	-3.197526232	-0.209051015	1.137303081
C	-2.428058172	-1.064357077	2.054306150
C	-2.975404214	-1.794094128	3.119157225
C	-2.139423152	-2.550746182	3.932588283
C	-0.767613055	-2.570111185	3.675986266
C	-0.282804020	-1.837691131	2.598445187
H	-2.689509194	1.747522128	-1.609990114
H	-6.337206438	0.963720069	0.501814036
H	-5.131546372	-0.487335035	2.070305147
H	-4.041895289	-1.769440125	3.308011240
H	-2.555125182	-3.117199227	4.760573345
H	-0.082171006	-3.141383225	4.292277308
H	0.769547055	-1.791885131	2.355767168
N	-1.092480080	-1.108276080	1.814651130
C	0.004043000	1.186396088	-5.450044378
H	-0.830591059	0.497416036	-5.615115400
H	0.922417065	0.650023044	-5.722146420
H	-0.100187007	2.029417145	-6.139249431
C	-5.322471385	2.382908171	-1.609558115
H	-5.388831376	3.398463242	-1.197662086
H	-6.346571446	2.039213147	-1.786170128
H	-4.809949344	2.455128176	-2.573454183
O	-1.005554071	-1.579462111	-1.221472089
C	-0.557543039	-2.801503203	-1.010798074
C	-0.960013071	-3.735763271	-2.146013155
O	0.103014007	-3.188840232	-0.054971004
H	-0.411201030	-3.465368249	-3.054948217
H	-0.724598052	-4.768121344	-1.880855135
H	-2.027267145	-3.643393260	-2.367211171
O	1.608869116	-0.470114034	-0.269128019
C	2.463546180	-0.469527034	0.702061049
C	3.870618277	-0.874102060	0.265001019
H	4.176564300	-0.314196022	-0.623994043
H	3.877148277	-1.937125140	-0.002127000
H	4.588279329	-0.707278049	1.071058075
O	2.229433159	-0.198917015	1.889564133

4

Pt	-0.532627982	0.023005735	-0.002094397
C	-0.154534872	1.148487333	-1.637090154
C	-0.201142289	0.712838596	-2.954106698
C	0.106759631	1.594038971	-4.007235787
C	0.457813846	2.918029071	-3.698799182
C	0.515315147	3.359153787	-2.379561840
C	0.210800727	2.475537553	-1.333008206
C	0.241652553	2.820303617	0.086510928
C	0.573520048	4.066745447	0.628763332
C	0.560666645	4.248639680	2.006099632
C	0.219590995	3.180687823	2.839338128
C	-0.096355986	1.960378204	2.262373469
H	-0.472765660	-0.314442831	-3.176355491
H	0.691568073	3.608954473	-4.504096044
H	0.795008032	4.387420388	-2.170503679
H	0.838141360	4.882108864	-0.033676318

H	0.814650118	5.215780409	2.428230737
H	0.198093351	3.282219151	3.918033199
H	-0.359970362	1.096622893	2.856297062
N	-0.085673412	1.794654527	0.927553773
C	-2.515134384	0.442020097	0.104486495
C	-3.204731234	1.270501662	-0.775477915
C	-4.587899850	1.487699390	-0.640733057
C	-5.264867700	0.846619187	0.404529777
C	-4.585308967	0.017399104	1.291514222
C	-3.204284635	-0.198983974	1.158898742
C	-2.438716950	-1.063219674	2.070058201
C	-2.983825970	-1.787083133	3.138906358
C	-2.149844181	-2.559806263	3.939498067
C	-0.781764292	-2.602989375	3.666176219
C	-0.297833995	-1.871686642	2.588293721
H	-2.682400375	1.759692548	-1.589907118
H	-6.334111915	0.997912243	0.525064627
H	-5.138287735	-0.464062657	2.091366740
H	-4.047395605	-1.746938699	3.340752992
H	-2.564729712	-3.122417565	4.770257267
H	-0.098853935	-3.190238620	4.269720615
H	0.752339307	-1.855052469	2.333205309
N	-1.105313147	-1.124787828	1.818036001
C	0.080793131	1.111351864	-5.435628796
H	-0.760989328	0.434168449	-5.612625515
H	0.996591877	0.555470871	-5.674900681
H	0.009067288	1.946431965	-6.138276856
C	-5.309667201	2.409119114	-1.591844023
H	-5.147908627	3.459355208	-1.316710177
H	-6.388195841	2.227874557	-1.580618089
H	-4.947899392	2.286443244	-2.617843761
O	-1.032122224	-1.602689611	-1.186991333
C	-0.538579094	-2.789871760	-1.022149350
C	-1.019154010	-3.700696414	-2.184866523
O	0.177514961	-3.247961047	-0.151567506
O	1.611094666	-0.504001710	-0.217897888
C	2.454962914	-0.436909026	0.738044038
C	3.887874123	-0.767388991	0.235401867
O	2.300056594	-0.160001330	1.925918457
F	4.290968187	0.127880125	-0.696495142
F	4.793954412	-0.748727989	1.228902965
F	3.938853323	-1.991715107	-0.336038051
F	-0.677866409	-3.181389348	-3.385691602
F	-2.363216555	-3.835745719	-2.169446208
F	-0.483073327	-4.927592543	-2.10402458

6

Pt	-0.534588960	-0.151061333	-0.434199522
C	-0.090496565	0.961205524	-2.061472824
C	-0.066573070	0.516600956	-3.379076738
C	0.258245338	1.391869777	-4.427970087
C	0.557204027	2.731413359	-4.121822215
C	0.538054361	3.187643408	-2.808831377
C	0.212454532	2.305758632	-1.764059901
C	0.149977739	2.683687075	-0.353316070
C	0.432847131	3.949216863	0.174727202
C	0.337698929	4.167016429	1.543503856
C	-0.039736750	3.116297763	2.382964688
C	-0.309732431	1.879079534	1.817505840
H	-0.301631374	-0.520210842	-3.595042303
H	0.805185508	3.420498574	-4.924852744
H	0.769055312	4.228201167	-2.600953508
H	0.729271952	4.750918966	-0.490943931
H	0.557058720	5.147636016	1.953930777
H	-0.125715237	3.244314285	3.455718368
H	-0.603585952	1.029527144	2.419488321
N	-0.217149416	1.675490563	0.491286008
C	-2.539046501	0.143969770	-0.493544961
C	-3.232503446	0.845889679	-1.473474237
C	-4.632751310	0.978257002	-1.426989996
C	-5.324679377	0.381313086	-0.365955132
C	-4.643760018	-0.324536215	0.622430769
C	-3.247150744	-0.454944113	0.578173617
C	-2.480122811	-1.194511222	1.594076267
C	-3.017219430	-1.840445684	2.716008978
C	-2.164814896	-2.495222857	3.599260232
C	-0.788740542	-2.506005960	3.356341850
C	-0.313066312	-1.857514238	2.223360876
H	-2.696995798	1.299396788	-2.300671635
H	-6.406507702	0.468232192	-0.313444961
H	-5.209088571	-0.779809871	1.429716502
H	-4.085464219	-1.827269848	2.895927655
H	-2.572188042	-2.995200153	4.472769354
H	-0.098241547	-3.008178939	4.024605512
H	0.736321545	-1.834543024	1.955583133
N	-1.141148274	-1.221048969	1.383042582
C	0.312161617	0.900066306	-5.852951680
H	-0.291223965	-0.002264420	-5.987978346
H	1.342218859	0.652271365	-6.140885806
H	-0.042620889	1.663640728	-6.552670215
C	-5.356942573	1.758632812	-2.495538464
H	-5.148749908	2.832299959	-2.406889876
H	-6.439489150	1.621448225	-2.424713222
H	-5.035852747	1.453101829	-3.497467040
O	-0.876028318	-1.803966406	-1.616048844
C	-0.377623358	-2.993499007	-1.346295027
C	-0.638387818	-3.970195200	-2.484969423
O	0.234077882	-3.320119720	-0.336033774
O	1.588308415	-0.515730427	0.043532287
C	2.435963514	-1.135226955	-0.690881460
O	2.415259962	-1.434742014	-1.877428141
C	3.694883545	-1.508449107	0.143792766
F	3.368951905	-2.241592826	1.236082731
F	4.332073845	-0.398513814	0.587861715
F	4.585711840	-2.225150660	-0.564953526
H	0.050560040	-3.749095267	-3.308117959
H	-1.656896687	-3.869854071	-2.869146148
H	-0.465596530	-4.993419256	-2.146017753

9

Pt	-0.511928246	0.037768397	-0.001088727
C	-0.150263276	1.159864014	-1.639097329
C	-0.203430222	0.723050949	-2.956079074
C	0.085906492	1.605316257	-4.013091335

C	0.426574135	2.933593830	-3.710397863
C	0.491056382	3.376838684	-2.392334051
C	0.204463086	2.491547679	-1.341885509
C	0.242987936	2.838922268	0.076672525
C	0.570504971	4.088713272	0.614774564
C	0.566630748	4.271993204	1.991843449
C	0.239951524	3.201865983	2.828221119
C	-0.070065696	1.978324351	2.254570205
H	-0.465947573	-0.307394103	-3.174278004
H	0.645852731	3.625617461	-4.518901297
H	0.761626748	4.408350671	-2.186972992
H	0.824559380	4.905054233	-0.050653446
H	0.816560850	5.241372982	2.411371817
H	0.226089234	3.304213700	3.907014644
H	-0.320217965	1.112029899	2.849979295
N	-0.070879812	1.812141173	0.920376378
C	-2.504785598	0.448846433	0.107170956
C	-3.203711166	1.280152585	-0.765031258
C	-4.588385804	1.487032997	-0.632545473
C	-5.263930364	0.831677735	0.405046530
C	-4.579627339	-0.000267528	1.285856455
C	-3.196584027	-0.204452766	1.153075156
C	-2.427152639	-1.067519636	2.062872238
C	-2.974319136	-1.809649235	3.118567229
C	-2.138462287	-2.573929998	3.925189416
C	-0.766000197	-2.588218932	3.672149339
C	-0.279940507	-1.841279403	2.605515228
H	-2.684210907	1.781081263	-1.574754625
H	-6.334581824	0.974435136	0.524598582
H	-5.130853620	-0.491618631	2.081110565
H	-4.041200828	-1.789657374	3.305425801
H	-2.555093489	-3.150583008	4.745519310
H	-0.080911731	-3.165312350	4.283203945
H	0.773789570	-1.789748234	2.368604061
N	-1.089956487	-1.105803912	1.826932609
C	0.052201556	1.120221118	-5.440773722
H	-0.777289501	0.425292575	-5.606617290
H	0.976408946	0.583584560	-5.691609717
H	-0.045987601	1.952569443	-6.143584927
C	-5.315224938	2.412493948	-1.576306569
H	-5.171711390	3.460938300	-1.284392941
H	-6.391276446	2.216084536	-1.577723584
H	-4.943119371	2.310700983	-2.600925938
O	-1.001442760	-1.594020910	-1.185964189
C	-0.531553736	-2.788040828	-1.011713080
C	-0.967726645	-3.678094471	-2.208530419
O	0.133334144	-3.271446304	-0.114181456
O	1.595968753	-0.495849024	-0.243946503
C	2.454449327	-0.461194569	0.726351327
C	3.861789394	-0.867749118	0.296304403
O	2.217738045	-0.151526365	1.902702842
F	-0.552623881	-3.154499042	-3.384649737
F	-2.313310822	-3.787279517	-2.267497683
F	-0.461145200	-4.917026329	-2.114168527
H	3.858277885	-1.910576947	-0.039507914
H	4.564741334	-0.759285028	1.124698863
H	4.193763396	-0.256518291	-0.549026121

11

Pt	-0.485474799	-0.138246174	-0.450534542
C	-0.030527077	1.009369433	-2.047240813
C	-0.001474136	0.600037771	-3.376970666
C	0.313185382	1.503094077	-4.403623070
C	0.599840504	2.838738109	-4.066101586
C	0.572836150	3.262266348	-2.743279137
C	0.253753262	2.351388803	-1.720439878
C	0.171413047	2.699695051	-0.304029866
C	0.440457744	3.955450094	0.255097314
C	0.321568780	4.144241354	1.626085077
C	-0.066253910	3.074549726	2.436742288
C	-0.320831276	1.848434028	1.840742785
H	-0.221204607	-0.433759134	-3.618802494
H	0.841664151	3.549117550	-4.852413459
H	0.790538496	4.300182502	-2.509214880
H	0.744837986	4.772130279	-0.388510239
H	0.530290654	5.116721995	2.060839658
H	-0.171688273	3.179781924	3.510291873
H	-0.621610518	0.985317936	2.419510875
N	-0.204799429	1.672683410	0.512602706
C	-2.501019068	0.137274844	-0.558392596
C	-3.185931578	0.847965319	-1.541307469
C	-4.587155552	0.963273258	-1.527153522
C	-5.298254911	0.337161101	-0.494282585
C	-4.631723103	-0.377608961	0.496063688
C	-3.231752141	-0.489052216	0.481914541
C	-2.482993348	-1.239340108	1.504121395
C	-3.042093396	-1.919750669	2.595164632
C	-2.205327738	-2.577394384	3.491301411
C	-0.822011117	-2.556219436	3.293377589
C	-0.325070604	-1.875891315	2.188393103
H	-2.638060015	1.322284800	-2.348867226
H	-6.382375526	0.408633580	-0.467059487
H	-5.209855603	-0.855633795	1.281010228
H	-4.115545638	-1.930852961	2.741612838
H	-2.630552990	-3.103540924	4.340696371
H	-0.143283096	-3.058087921	3.973929583
H	0.730388913	-1.818640380	1.950070911
N	-1.138054344	-1.239043832	1.334926976
C	0.370254825	1.048578472	-5.841149017
H	-0.172023509	0.109890758	-5.986273972
H	1.408321262	0.881845590	-6.157092812
H	-0.052616640	1.801139684	-6.515032324
C	-5.300442054	1.764131229	-2.588091390
H	-5.362583179	2.822723078	-2.303549137
H	-6.323864874	1.406948402	-2.737207760
H	-4.773491255	1.717519503	-3.546166429
O	-0.804235121	-1.791490999	-1.647039861
C	-0.346663107	-2.989816785	-1.359188404
C	-0.744624029	-4.035953099	-2.437961523
O	0.293428030	-3.302671889	-0.360876481
O	1.593109101	-0.491490902	0.103828688
C	2.510756834	-1.015314057	-0.657606175
O	2.457883750	-1.180354831	-1.877329360
C	3.748551860	-1.449996506	0.129942421

H	4.591885793	-1.619043268	-0.543553280	H	-2.596636708	-4.894986948	-3.204739093
H	4.020358145	-0.707691670	0.886071917	H	-2.673700431	-4.464749779	-1.488328454
H	3.527634218	-2.387311715	0.654316598	H	-2.746161878	-3.193505596	-2.721666337
C	-0.234125838	-3.585456892	-3.820690885	C	-0.129003769	-5.396675053	-2.078483761
H	-0.698259662	-2.645650740	-4.130572103	H	-0.467545261	-5.740400918	-1.096850412
H	0.852481413	-3.444210054	-3.816752388	H	-0.418581803	-6.146266110	-2.823921078
H	-0.470073953	-4.345847943	-4.574475618	H	0.963941244	-5.345076356	-2.054048800
C	-2.283686215	-4.150179030	-2.463628864				

4. References

(1) Sheldrick, G. M. A Short History of SHELX. *Acta Crystallogr., Sect. A: Found. Crystallogr.* **2008**, *64*, 112–122.

(2) Sheldrick, G. M. SHELXT – Integrated Space-Group and Crystal-Structure Determination. *Acta Crystallogr., Sect. A: Found. Crystallogr.* **2015**, *71*, 3–8.
